# Association of enteropathogen detection with diarrhoea by age and high versus low child mortality settings: a systematic review and meta-analysis

**DOI:** 10.1016/S2214-109X(21)00316-8

**Published:** 2021-09-14

**Authors:** Julia M Baker, Mateusz Hasso-Agopsowicz, Virginia E Pitzer, James A Platts-Mills, Andre Peralta-Santos, Catherine Troja, Helena Archer, Boya Guo, William Sheahan, Jairam Lingappa, Mark Jit, Benjamin A Lopman

**Affiliations:** a Department of Epidemiology, Rollins School of Public Health, Emory University, Atlanta, GA, USA; b Vaccine Product Delivery Research, Immunization, Vaccines and Biologicals, WHO, Geneva, Switzerland; c Department of Epidemiology of Microbial Diseases, Yale School of Public Health, Yale University, New Haven, CT, USA; d Division of Infectious Diseases & International Health, University of Virginia, Carter-Harrison Research Building, Charlottesville, VA, USA; e Department of Epidemiology, University of Washington, Seattle, WA, USA; f Department of Global Health, University of Washington, Seattle, WA, USA; g Department of Infectious Disease Epidemiology, London School of Hygiene & Tropical Medicine, London, UK

## Abstract

**Background:**

The odds ratio (OR) comparing pathogen presence in diarrhoeal cases versus asymptomatic controls is a measure for diarrhoeal disease cause that has been integrated into burden of disease estimates across diverse populations. This study aimed to estimate the OR describing the association between pathogen detection in stool and diarrhoea for 15 common enteropathogens by age group and child mortality setting.

**Methods:**

We did a systematic review to identify case-control and cohort studies published from Jan 1, 1990, to July 9, 2019, which examined at least one enteropathogen of interest and the outcome diarrhoea. The analytical dataset included data extracted from published articles and supplemented with data from the Global Enteric Multicenter Study and the Malnutrition and Enteric Disease study. Random effects meta-analysis models were fit for each enteropathogen, stratified by age group and child mortality level, and adjusted for pathogen detection method and study design to produce summary ORs describing the association between pathogen detection in stool and diarrhoea.

**Findings:**

1964 records were screened and 130 studies (over 88 079 cases or diarrhoea samples and 135 755 controls or non-diarrhoea samples) were available for analysis. Heterogeneity (*I*^2^) in unadjusted models was substantial, ranging from 27·6% to 86·6% across pathogens. In stratified and adjusted models, summary ORs varied by age group and setting, ranging from 0·4 (95% CI 0·2–0·6) for *Giardia lamblia* to 54·1 (95% CI 7·4–393·5) for *Vibrio cholerae*.

**Interpretation:**

Incorporating effect estimates from diverse data sources into diarrhoeal disease cause and burden of disease models is needed to produce more representative estimates.

**Funding:**

WHO, Bill & Melinda Gates Foundation, and National Institutes of Health.

## Introduction

The development of vaccines against enteropathogens has been identified as a public health priority to reduce the diarrhoeal disease burden.^1^, ^2^ The success of such a strategy will depend on correctly identifying, and then developing vaccines against, the enteropathogens that contribute most to diarrhoeal disease mortality.

A central challenge in accurately estimating burden is determining the proportion of diarrhoeal disease attributable to a particular enteropathogen. Presence of an enteropathogen in stool does not necessarily indicate the cause of diarrhoea, because enteropathogens can often be detected in the stool of healthy, asymptomatic individuals.^3^ An approach that has been adopted for ascribing cause is calculation of the odds ratio (OR) comparing the prevalence of a pathogen in stool of diarrhoeal patients versus asymptomatic controls; this gives an indication of the strength of association of the pathogen and the syndrome of diarrhoea.^4^

The ORs from individual studies have been used to parameterise attribution models that estimate the burden of disease for specific pathogens. The Global Burden of Diseases, Injuries, and Risk Factors Study (GBD) uses a model that apportions diarrhoeal mortality based on a pathogen-specific population attributable fraction.^5^ This attributable fraction is a function of the OR quantifying the relationship between pathogen detection and the odds of having diarrhoea. However, because the studies incorporated into the GBD estimates do not always have controls, the ORs used in the calculations are derived from the Global Enteric Multicenter Study (GEMS), a study among children younger than 5 years in seven low-income and middle-income countries.^6^ This application requires a critical assumption that the ORs from GEMS are generalisable across age groups, settings, and the range of disease outcomes for which GBD produces burden estimates. Limiting ORs to those produced by a single study—even one as rigorously conducted as GEMS—might result in incorrect assumptions about diarrhoeal disease cause, leading to inaccurate attribution of pathogen-specific burden of disease estimates, risking misguided vaccine investment and public health intervention efforts.

**Research in context**

**Evidence before this study**

Enteropathogens can often be detected in the stool of healthy, asymptomatic individuals. An approach that has been adopted to estimate cause of diarrhoeal diseases is the odds ratio (OR) comparing pathogen presence in diarrhoeal cases versus asymptomatic controls. This approach, originally based on evidence from low-income and middle-income settings, is now being incorporated into burden of enteric disease estimates for the global population. Whether the relationship between detection of an enteropathogen in stool and the occurrence of diarrhoea varies by age group and setting has not been systematically evaluated.We did a systematic review and meta-analysis to estimate pathogen-specific ORs describing the relationship between pathogen detection and diarrhoea for 15 enteropathogens stratified by age group and child mortality setting. We searched Embase, Cochrane, MEDLINE, and PubMed to identify case-control and cohort studies that examined at least one enteropathogen of interest and the outcome diarrhoea. From Jan 1, 1990, to July 9, 2019, 128 unique studies were published and met our inclusion criteria. Search terms included (diarrh* OR gastroenteritis OR enteric infection*) AND (aeromonas OR entamoeba OR cryptosporidium OR giardia lamblia OR adenovir* OR astrovir* OR sapovirus OR norovirus OR rotavirus OR Escherichia coli OR etec OR epec OR E. coli OR cholera* OR campylobacter OR shigell* OR salmonell*). The search was restricted to articles describing human studies and those published in English, French, Spanish, Portuguese, Italian, or Chinese. The analytical dataset included effect estimates extracted from these studies and supplemental data from two additional studies—namely, the Global Enteric Multicenter Study and the Malnutrition and Enteric Disease study. Random effects meta-analysis models were fit for each enteropathogen, stratified by age group and child mortality level and adjusted for pathogen detection method and study design.

**Added value of this study**

We provide a comprehensive quantification of pathogen-specific, age-specific, and setting-specific estimates of the association between detection of 15 specific pathogens in stool and diarrhoeal disease. We found that pathogen-specific ORs varied by age group and setting after accounting for pathogen detection method and study design, with variability probably reflecting differences in epidemiology, immunity characteristics, and study characteristics. The wide CIs that accompanied the ORs represented considerable uncertainty and heterogeneity among studies; such heterogeneity might arise from population differences (eg, age composition), our inability to distinguish between many pathogen subtypes, and other study differences.

**Implications of all the available evidence**

The magnitude of the association between pathogen detection in stool and symptomatic diarrhoea varies by pathogen, age group, and setting, emphasising the importance of building burden of disease estimates on diverse data sources. The OR estimates produced in this analysis can be integrated into or adapted for burden of disease models to strengthen their ability to produce reliable pathogen-specific estimates by age and location. More accurate burden of disease estimates can better inform decision making and prioritisation of public health measures.

In 2018, the Product Development for Vaccines Advisory Committee recommended WHO establish the Burden of Enteric Disease Working Group to explore differences in modelling approaches and recent enteric disease burden estimates.^7^ In support of this effort, our study examines the relationship between detection of an enteropathogen in stool and the occurrence of diarrhoea. Specifically, we did a systematic review and meta-analysis to determine pathogen-specific ORs of having diarrhoea when a pathogen is detected in stool for 15 common enteropathogens stratified by age group and child mortality level. A standardised and comprehensive summary of ORs across settings and age groups could inform future modelling efforts and improve diarrhoeal disease burden estimates.

## Methods

### Search strategy and selection criteria

We did a systematic review and meta-analysis. Adhering to the PRISMA guidelines, literature from Jan 1, 1990, to July 9, 2019, were compiled from Embase, Cochrane, MEDLINE, and PubMed databases, using the search terms (diarrh* OR gastroenteritis OR enteric infection*) AND (aeromonas OR entamoeba OR cryptosporidium OR giardia lamblia OR adenovir* OR astrovir* OR sapovirus OR norovirus OR rotavirus OR Escherichia coli OR etec OR epec OR E. coli OR cholera* OR campylobacter OR shigell* OR salmonell*) to identify studies that quantified the association between enteropathogens and diarrhoea. Studies were included if they were case-control or cohort in design and examined at least one enteropathogen of interest and the outcome diarrhoea. Articles were limited to those published in English, French, Spanish, Portuguese, Italian, and Chinese. Studies were excluded if they did not report on non-diarrhoeal controls; included participants with a broad case definition of gastroenteritis which prevented the reviewer from determining if all cases had diarrhoea; were limited to nosocomial infections; or were conducted solely among patients with underlying chronic conditions (except HIV). Additional details on inclusion and exclusion criteria are provided in the appendix (p 1).

Information extracted from each study included, but was not limited to, study design, number of cases or diarrhoea samples and controls or non-diarrhoea samples, pathogens detected, pathogen detection method, and all provided measures of association between pathogen presence in stool and diarrhoea. We considered 15 pathogens of interest, including five viruses (adenovirus 40/41, astrovirus, norovirus, rotavirus, and sapovirus), seven bacteria (*Aeromonas*, *Campylobacter*, *Vibrio cholerae*, enteropathogenic *Escherichia coli* [EPEC], enterotoxigenic *E coli*, *Salmonella enterica*, and *Shigella*), and three parasites (*Cryptosporidium*, *Entamoeba histolytica*, and *Giardia lamblia*). These enteropathogens were selected because they are pathogens for which recent burden of disease modelling groups have produced estimates or are of special interest to the WHO Burden of Enteric Disease Working Group, or both.^7^ When available, strain-specific data were extracted (appendix p 2). WHO data on the year of national-level rotavirus vaccine introduction were used to determine if the study was done before or after rotavirus vaccine introduction in the country. A study was considered after vaccine if it began at least 1 year after the year rotavirus vaccine was introduced to account for possible low vaccine coverage in the year of introduction. The data collection process is detailed in the appendix (p 2).

Data from the systematic review were supplemented with case-control data from GEMS and nested case-control data from the Etiology, Risk Factors and Interactions of Enteric Infections and Malnutrition and the Consequences for Child Health and Development (MAL-ED) study. The nested case-control dataset from MAL-ED was created by identifying an appropriate age-matched control for each case in the MAL-ED cohort study. For each of 6625 episodes of diarrhoea that had completely valid TaqMan Array Card (Thermo Fisher, Carlsbad, CA, USA) results in MAL-ED,^8^ we attempted to identify an appropriate non-diarrhoea sample from the same individual, obtained no more than 60 days before the onset of diarrhoea and more than 7 days after any study-defined day of diarrhoea. In the case that more than one such control was identified, the sample obtained closest to the diarrhoea sample was used. An appropriate matched control was identified for 5646 of 6625 episodes; the median age difference between the case and control was 24 days (IQR 14–31). To calculate ORs, we used the proportion of stool samples positive by pathogen, stool type (case or control), age group, and study site. These data were included because measures of association from these studies were not available for extraction in the literature review and because of the studies’ sizes and influence on the field of childhood gastroenteritis.

The dataset was examined to identify possible overlap in studies by comparing the country, region, study start year and month, and study stop year and month described in each article. Where there appeared to be overlap based on these criteria, the full-text article and detailed data extracted from each were examined further. Duplicate data were excluded from analysis. Studies that differed in the pathogens reported, age groups reported, or study design were retained in the analytical dataset.

### Data analysis

Extracted data included all available pathogen-specific measures of association for each unique combination of pathogen, strain, age group, severity, study presentation, laboratory detection method, and country reported in the study. Multiple effect estimates could be extracted from a single study (based on the unique combinations of pathogen strain, age group, and other factors described above). Each effect estimate was represented by an observation in the dataset. Available measures of association included ORs (adjusted or unadjusted) and relative risk (RR; adjusted or unadjusted). The number of cases or diarrhoea samples, the number of controls or non-diarrhoea samples, and the proportion of each that were positive for a particular pathogen, if provided, were used to calculate unadjusted ORs if an effect estimate was not provided for a particular stratum in the article. A hierarchy was arranged, prioritising the best available measure of association from each observation for use in the meta-analysis (from most to least preferred)—namely, adjusted OR, unadjusted OR, adjusted RR, unadjusted RR, unadjusted OR-calculated. For observations indicating co-infection, the reported effect estimate was used to represent the association between each individual pathogen detected and diarrhoea. For example, if an OR of 1·8 was estimated for the association between rotavirus and *G lamblia* coinfection and diarrhoea, two effect estimates were created and 1·8 was used to represent the association between rotavirus and diarrhoea, as well as *G lamblia* and diarrhoea. These co-infections were included in the primary analysis as described, and excluded from a sensitivity analysis.

The quality of each published study was assessed via a validity score. One point was awarded for each of the following criteria (maximum of five): case or outcome-positive definition provided; control or outcome-negative definition provided; diarrhoea presentation defined; laboratory certification or quality framework described; and, diarrhoea definition provided.

An unadjusted random effects model was fit for each pathogen using the DerSimonian-Laird estimator to produce forest plots and examine heterogeneity using the *I*^2^. Pathogen-specific heterogeneity was first examined using only OR effect estimates in the models and then using both ORs and RRs to determine the impact of including RRs. Including RRs had almost no impact on heterogeneity for each pathogen, so ORs and RRs were used together for the subsequent analysis steps. Influential outliers were identified using studentised residual plots and Cook's distances. We then examined unadjusted ORs stratified by whether or not PCR was used as the pathogen detection method to determine if patterns were apparent by detection method.

Four factors of interest were incorporated into the adjusted models: age group, child mortality level, pathogen detection method, and study design. Five age group categories were created, including 0–1 year, 2–4 years, 0–4 years, and 5 years or older, and a mixed group, which included strata that spanned or were unable to be assigned to the previous four age groups. In the data extracted for some studies, it was unclear whether an age maximum of 2 years or 5 years meant the age group was inclusive of age 2 years or 5 years. After re-examining the full-text articles for several studies to review the specific wording, it was clear that a majority of studies specifically stated or meant younger than age 2 years or 5 years. We, therefore, included studies that indicated an age maximum of 2 years in the 0–1 year group and maximum of 5 years in the 2–4 years group. Child mortality levels were assigned based on country-level UN Inter-agency Group for Child Mortality Estimation estimates of mortality rates for children younger than 5 years for 2003 (the median study start year in the dataset).^9^ Using categorisations proposed by WHO,^10^ countries were divided into child mortality quintiles. The three lowest quintiles were grouped together as very low and low child mortality, and the two highest quintiles were grouped as high child mortality. Pathogen detection method was categorised as conventional (ELISA, culture, isolation, microscopy), PCR, or other or unspecified. Study design was categorised as case-control (standard or nested) or other (prospective cohort, retrospective cohort, cross-sectional, and randomised controlled trial).

The adjusted models were specified with the goal of producing summary ORs (and 95% CIs) specific to age groups and settings that could then be used in future burden of disease models. Random effects meta-analysis models, again using the DerSimonian-Laird estimator, were fit incorporating four factors of interest. The models were stratified by age group. The 0–1 year, 2–4 years, and 0–4 years age groups were further stratified by child mortality level; however, the 5 years or older category was not further stratified due to small sample size. We adjusted for pathogen detection method and study design with conventional detection methods and case-control study design as the reference groups. We did three sensitivity analyses by excluding outliers and influential observations, RRs (ie, included only ORs), and observations that indicated co-infections from the dataset. A fourth sensitivity analysis re-categorised age without making the assumptions previously described regarding the 2-year and 5-year age maximums. Lastly, a subanalysis was done in which our primary model (adjusted for pathogen detection method and study design) for children 0–4 years of age was fit using regional stratifications (African region, and south Asian and southeast Asian region) among high child mortality settings. Analyses were done using R, version 3.6.3, using the *metafor* package.^11^

### Role of the funding source

The funder of the study had no role in study design, data collection, data analysis, data interpretation, or writing of the report.

## Results

**Figure 1:**
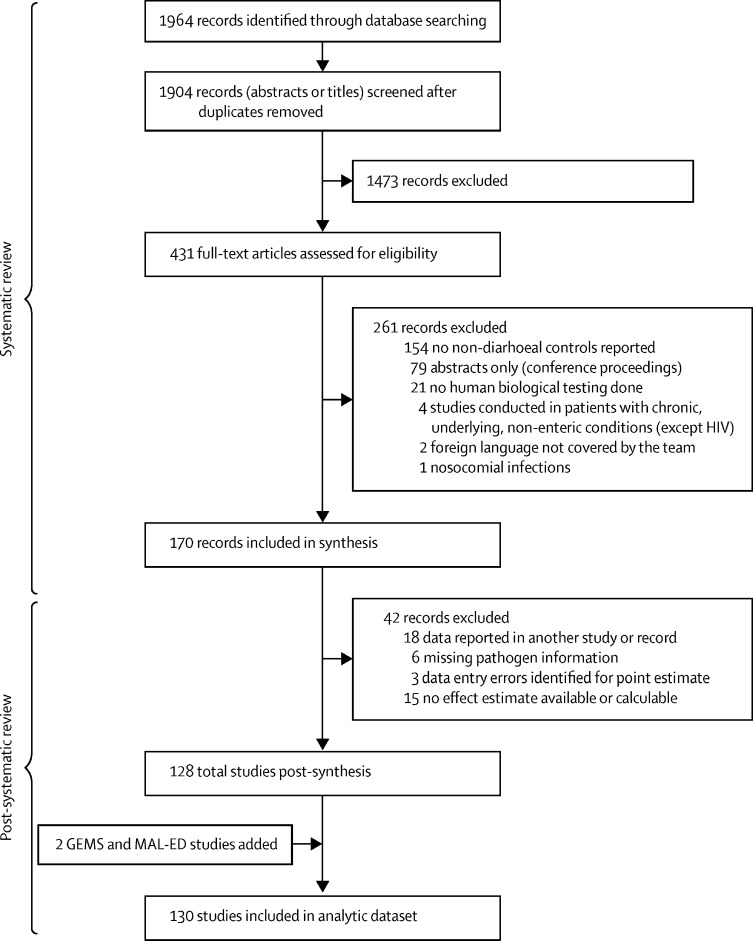
Study selection Detailed search terms and protocols for the literature search are available in the appendix (pp 1–2). Studies identified from the literature review were supplemented with data from the Global Enteric Multicenter Study (GEMS) and Malnutrition and Enteric Disease study (MAL-ED).

The systematic review identified 1964 records. Of those, 170 remained after screening and full-text review (figure 1). After the systematic review was completed, data from two additional studies, GEMS and MAL-ED, were added to the dataset. In preparation for analysis, 18 records were excluded due to duplicate data reported from another study or reference in the dataset, six were missing pathogen information, 15 were excluded because an effect estimate was unavailable from the publication and unable to be calculated, and three were excluded because of data entry errors identified by extreme point estimates or CIs. The final analytic dataset was comprised of 130 studies (figure 1; appendix pp 3–10) and included 1240 stratum-specific observations (a single study could contribute multiple observations, as described earlier). The studies included more than 88 079 cases or diarrhoea samples and 135 755 controls or non-diarrhoea samples. Examination of outliers and influential observations identified 27 observations that were considered both outliers and influential (and were excluded in a sensitivity analysis).

The majority (1186 [95·6%]) of effect estimates from the 1240 observations were ORs and three-quarters of the studies (n=835 [67·2%]) had a validity score of 3 or more (appendix p 32). Heterogeneity by pathogen, assessed in unadjusted models, was considered moderate (*I*^2^ values of 30–60%) or substantial (*I*^2^ values of 50–90%)^12^ for nearly all pathogens (*I*^2^ range 27·6–86·6; appendix p 33). *I*^2^ values were largely unchanged when RRs were excluded from the models (appendix p 33).

The distribution of factors varied by pathogen (Table 1, Table 2). 904 (72·9%) observations were for children younger than 5 years, 510 (41·1%) for 0–1 years, 106 (8·5%) for 2–4 years, and 98 (7·9%) were among older children and adults. More than half of observations were collected from studies in high child mortality settings (n=723 [58·3%]). 47 (36·1%) reported using multiple pathogen detection methods. The most common method of pathogen detection was PCR (n=689 [55·6%]). 999 (80·6%) observations were from case-control studies. 359 (29·0%) observations described cases or diarrhoea samples and controls or non-diarrhoea samples identified in the community setting, while 519 (41·9%) were from a facility setting.

**Table 1:** Distribution of study characteristics by enteropathogen including 1240 observations from 130 studies

		**All pathogens**	**Adenovirus**	**Astrovirus**	**Norovirus**	**Rotavirus**	**Sapovirus**	**Aeromonas**	**Campylobacter**	**Cholera**
Total observations*		1240 (100·0%)	56 (4·5%)	48 (3·9%)	86 (6·9%)	113 (9·1%)	42 (3·4%)	15 (1·2%)	137 (11·0%)	5 (0·4%)
Age group, years										
	0–1	510 (41·1%)	28 (50·0%)	26 (54·2%)	36 (41·9%)	51 (45·1%)	26 (61·9%)	3 (20·0%)	59 (43·1%)	3 (60·0%)
	2–4	106 (8·5%)	7 (12·5%)	7 (14·6%)	7 (8·1%)	7 (6·2%)	7 (16·7%)	2 (13·3%)	10 (7·3%)	0
	0–4	904 (72·9%)	49 (87·5%)	43 (89·6%)	60 (69·8%)	82 (72·6%)	41 (97·6%)	11 (73·3%)	95 (69·3%)	4 (80·0%)
	Mixed	238 (19·2%)	6 (10·7%)	4 (8·3%)	20 (23·3%)	26 (23·0%)	1 (2·4%)	2 (13·3%)	27 (19·7%)	0
	≥5	98 (7·9%)	1 (1·8%)	1 (2·1%)	6 (7·0%)	5 (4·4%)	0	2 (13·3%)	15 (10·9%)	1 (20·0%)
Child mortality status										
	Very low	142 (11·5%)	9 (16·1%)	7 (14·6%)	10 (11·6%)	11 (9·7%)	4 (9·5%)	2 (13·3%)	12 (8·8%)	0
	Low	375 (30·2%)	10 (17·9%)	7 (14·6%)	28 (32·6%)	37 (32·7%)	9 (21·4%)	4 (26·7%)	36 (26·3%)	2 (40·0%)
	High	723 (58·3%)	37 (66·1%)	34 (70·8%)	48 (55·8%)	65 (57·5%)	29 (69·0%)	9 (60·0%)	89 (65·0%)	3 (60·0%)
Pathogen detection method										
	Enzyme immunoassay	176 (14·2%)	18 (32·1%)	11 (22·9%)	9 (10·5%)	58 (51·3%)	0	0	2 (1·5%)	0
	Culture	166 (13·4%)	0	0	0	0	0	5 (33·3%)	58 (42·3%)	3 (60·0%)
	Microscopy	72 (5·8%)	0	0	0	0	0	1 (6·7%)	6 (4·4%)	0
	PCR	689 (55·6%)	38 (67·9%)	37 (77·1%)	77 (89·5%)	48 (42·5%)	42 (100·0%)	2 (13·3%)	65 (47·4%)	1 (20·0%)
	Other or unspecified	137 (11·0%)	0	0	0	7 (6·2%)	0	7 (46·7%)	6 (4·4%)	1 (20·0%)
Study design										
	Case-control	999 (80·6%)	52 (92·9%)	45 (93·8%)	77 (89·5%)	86 (76·1%)	38 (90·5%)	12 (80·0%)	109 (79·6%)	4 (80·0%)
	Other†	241 (19·4%)	4 (7·1%)	3 (6·2%)	9 (10·5%)	27 (23·9%)	4 (9·5%)	3 (20·0%)	28 (20·4%)	1 (20·0%)
Presentation setting										
	Community	359 (29·0)	9 (16·1%)	7 (14·6%)	21 (24·4%)	28 (24·8%)	7 (16·7%)	4 (26·7%)	47 (34·3%)	1 (20·0%)
	Facility	519 (41·9)	17 (30·4%)	11 (22·9%)	35 (40·7%)	56 (49·6%)	5 (11·9%)	10 (66·7%)	58 (42·3%)	3 (60·0%)
	Unknown	362 (29·2)	30 (53·6%)	30 (62·5%)	30 (34·9%)	29 (25·7%)	30 (71·4%)	1 (6·7%)	32 (23·4%)	1 (20·0%)
Presentation of diarrhoea										
	Acute, watery only	204 (16·5%)	8 (14·3%)	4 (8·3%)	8 (9·3%)	23 (20·4%)	1 (2·4%)	1 (6·7%)	25 (18·2%)	0
	Persistent only	54 (4·4%)	1 (1·8%)	0	2 (2·3%)	5 (4·4%)	1 (2·4%)	0	3 (2·2%)	0
	Dysenteric (bloody) only	1 (0·1%)	0	0	0	0	0	0	1 (0·7%)	0
	Acute, watery, or dysenteric (bloody)	19 (1·5%)	1 (1·8%)	0	1 (1·2%)	1 (0·9%)	1 (2·4%)	0	1 (0·7%)	0
	Acute, watery, or persistent	8 (0·6%)	0	0	0	0	0	0	0	0
	Acute, watery, persistent, or dysenteric	5 (0·4%)	0	0	0	0	0	0	1 (0·7%)	0
	Not specified	949 (76·5%)	46 (82·1%)	44 (91·7%)	75 (87·2%)	84 (74·3%)	39 (92·9%)	14 (93·3%)	106 (77·4%)	5 (100·0%)

Data are n (%).

* Row percent.

† Other study designs include prospective cohort, retrospective cohort, cross-sectional, and randomised control trial.

**Table 2:** Distribution of study characteristics by enteropathogen including 1240 observations from 130 studies

		**aEPEC**	**tEPEC**	**EPEC-unknown subgroup**	**ST ETEC**	**LT ETEC**	**ETEC-unknown subgroup**	**Salmonella enterica**	**Shigella**	**Crypto-sporidium**	**Entamoeba histolytica**	**Giardia lamblia**
Total observations*		42 (3·4%)	36 (2·9%)	49 (4·0%)	90 (7·3%)	65 (5·2%)	63 (5·1%)	56 (4·5%)	86 (6·9%)	94 (7·6%)	59 (4·8%)	98 (7·9)%
Age group, years												
	0–1	25 (59·5%)	25 (69·4%)	12 (24·5%)	58 (64·4%)	37 (56·9%)	13 (20·6%)	11 (19·6%)	29 (33·7%)	37 (39·4%)	8 (13·6%)	23 (23·5%)
	2–4	7 (16·7%)	7 (19·4%)	3 (6·1%)	11 (12·2%)	9 (13·8%)	5 (7·9%)	0	9 (10·5%)	7 (7·45%)	0	1 (1·0%)
	0–4	38 (90·5%)	35 (97·2%)	36 (73·5%)	83 (92·2%)	59 (90·8%)	39 (61·9%)	35 (62·5%)	63 (73·3%)	59 (62·8%)	22 (37·3%)	50 (51·0%)
	Mixed	2 (4·8%)	0	9 (18·4%)	4 (4·4%)	4 (6·2%)	15 (23·8%)	15 (26·8%)	16 (18·6%)	25 (26·6%)	29 (49·2%)	33 (33·7%)
	≥5	2 (4·8%)	1 (2·8%)	4 (8·2%)	3 (3·3%)	2 (3·1%)	9 (14·3%)	6 (10·7%)	7 (8·1%)	10 (10·6%)	8 (13·6%)	15 (15·3%)
Child mortality status												
	Very low	6 (14·3%)	2 (5·6%)	4 (8·2%)	4 (4·4%)	2 (3·1%)	9 (14·3%)	8 (14·3%)	5 (5·8%)	16 (17·0%)	3 (5·1%)	28 (28·6%)
	Low	8 (19·0%)	6 (16·7%)	37 (75·5%)	31 (34·4%)	23 (35·4%)	34 (54·0%)	18 (32·1%)	27 (31·4%)	15 (16·0%)	17 (28·8%)	26 (26·5%)
	High	28 (66·7%)	28 (77·8%)	8 (16·3%)	55 (61·1%)	40 (61·5%)	20 (31·7%)	30 (53·6%)	54 (62·8%)	63 (67·0%)	39 (66·1%)	44 (44·9%)
Pathogen detection method												
	Enzyme immunoassay	0	0	3 (6·1%)	18 (20·0%)	11 (16·9%)	12 (19·0%)	0	0	5 (5·3%)	11 (18·6%)	18 (18·4%)
	Culture	0	0	3 (6·1%)	1 (1·1%)	0	2 (3·2%)	30 (53·6%)	46 (53·5%)	5 (5·3%)	4 (6·8%)	9 (9·2%)
	Microscopy	0	0	0	0	0	0	6 (10·7%)	3 (3·5%)	18 (19·1%)	13 (22·0%)	25 (25·5%)
	PCR	42 (100·0%)	36 (100·0%)	26 (53·1%)	53 (58·9%)	46 (70·8%)	36 (57·1%)	15 (26·8%)	35 (40·7%)	51 (54·3%)	22 (37·3%)	17 (17·3%)
	Other or unspecified	0	0	17 (34·7%)	18 (20·0%)	8 (12·3%)	13 (20·6%)	5 (8·9%)	2 (2·3%)	15 (16·0%)	9 (15·3%)	29 (29·6%)
Study design												
	Case-control	41 (97·6%)	36 (100·0%)	32 (65·3%)	50 (55·6%)	43 (66·2%)	48 (76·2%)	50 (89·3%)	78 (90·7%)	72 (76·6%)	53 (89·8%)	73 (74·5%)
	Other†	1 (2·4%)	0	17 (34·7%)	40 (44·4%)	22 (33·8%)	15 (23·8%)	6 (10·7%)	8 (9·3%)	22 (23·4%)	6 (10·2%)	25 (25·5%)
Presentation setting												
	Community	2 (4·8%)	1 (2·8%)	24 (49·0%)	49 (54·4%)	25 (38·5%)	32 (50·8%)	13 (23·2%)	15 (17·4%)	24 (25·5%)	14 (23·7%)	36 (36·7%)
	Facility	11 (26·2%)	6 (16·7%)	25 (51·0%)	10 (11·1%)	9 (13·8%)	31 (49·2%)	43 (76·8%)	41 (47·7%)	42 (44·7%)	45 (76·3%)	61 (62·2%)
	Unknown	29 (69·0%)	29 (80·6%)	0	31 (34·4%)	31 (47·7%)	0	0	30 (34·9)	28 (29·8%)	0	1 (1·0%)
Presentation of diarrhoea												
	Acute, watery only	6 (14·3%)	3 (8·3%)	17 (34·7)	15 (16·7)	10 (15·4)	11 (17·5)	13 (23·2)	15 (17·4)	11 (11·7)	11 (18·6)	22 (22·4)
	Persistent only	0	0	3 (6·1%)	2 (2·2%)	2 (3·1%)	5 (7·9%)	3 (5·4%)	5 (5·8%)	9 (9·6%)	3 (5·1%)	10 (10·2%)
	Dysenteric (bloody) only	0	0	0	0	0	0	0	0	0	0	0
	Acute, watery, or dysenteric (bloody)	0	0	1 (2·0%)	0	0	2 (3·2%)	2 (3·6%)	2 (2·3%)	2 (2·1%)	3 (5·1%)	2 (2·0%)
	Acute, watery, or persistent	0	0	0	0	0	0	0	0	4 (4·3%)	1 (1·7%)	3 (3·1%)
	Acute, watery, persistent, or dysenteric	0	0	0	0	0	0	1 (1·8%)	1 (1·2%)	0	1 (1·7%)	1 (1·0%)
	Not specified	36 (85·7%)	33 (91·7%)	28 (57·1%)	73 (81·1%)	53 (81·5%)	45 (71·4%)	37 (66·1%)	63 (73·3%)	68 (72·3%)	40 (67·8%)	60 (61·2%)

Data are n (%). aEPEC=atypical enteropathogenic *Escherichia coli*. tEPEC=typical enteropathogenic *E coli*. ST ETEC=heat-stable enterotoxigenic *E coli* (ie, enterotoxigenic *E coli* that harbour the ST gene regardless of LT gene status). LT ETEC=heat-labile enterotoxigenic E coli (enterotoxigenic *E coli* that only harbour the LT gene and not the ST gene).

* Row percent.

† Other study designs include prospective cohort, retrospective cohort, cross-sectional, and randomised control trial.

Forest plots from the unadjusted models showed a wide range of effect estimates for each pathogen (appendix pp 11–31). When models were fit stratified by PCR versus non-PCR detection method (and not adjusted further), no clear patterns were apparent (appendix p 34) so detection method was included as a predictor in subsequent models.

**Figure 2:**
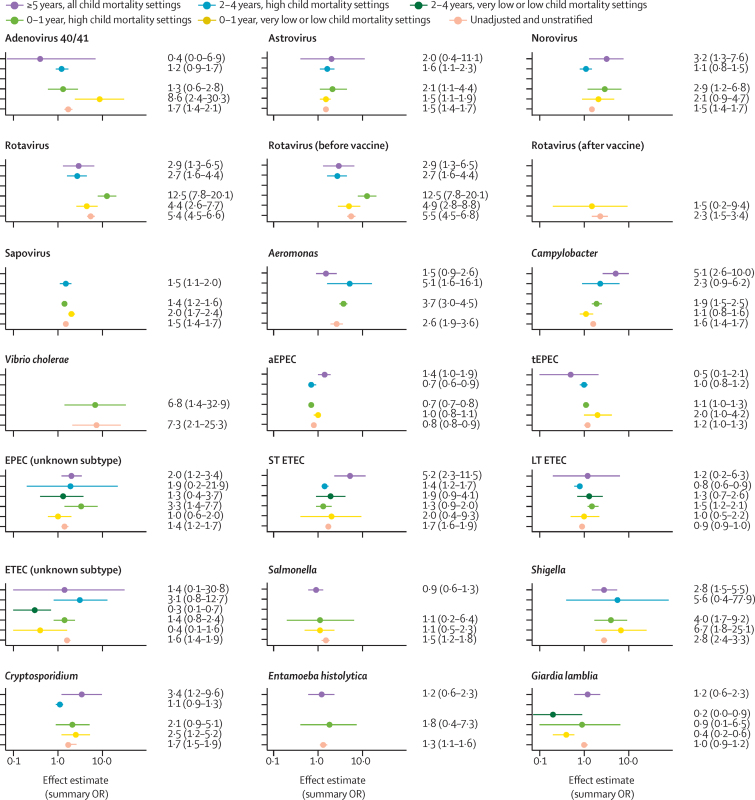
Unadjusted and adjusted random effects meta-analysis model results by pathogen Unadjusted model results (pink) represent the crude summary estimate for a given pathogen. Adjusted models (yellow, light green, dark green, blue, purple) are stratified by age and child mortality setting and adjusted for pathogen detection method and study design with conventional detection methods and case-control study design as the reference groups. aEPEC=atypical enteropathogenic *E coli.* tEPEC=typical enteropathogenic *E coli.* ST ETEC=heat-stable enterotoxigenic *E coli* (ie, enterotoxigenic *E coli* that harbour the ST gene regardless of LT gene status). LT ETEC=heat-labile enterotoxigenic *E coli* (ie, enterotoxigenic *E coli* that only harbour the LT gene and not the ST gene). OR=odds ratio.

In adjusted models, ORs varied by pathogen and pathogen strain, many with wide CIs (figure 2; appendix pp 35–36). Differences in ORs were observed for some pathogens when comparing child mortality settings within the 0–4-years age group. The OR for viral pathogens (except rotavirus) appeared to be higher in very low or low child mortality settings when compared with high child mortality settings; however, the CIs overlapped. For example, the OR for adenovirus 40/41 among children aged 0–4 years was 6·0 (95% CI 2·2–16·4) in very low or low child mortality settings compared with 1·3 (0·7–2·3) in high child mortality settings. This pattern was not consistent across bacterial or parasitic enteropathogens. For some pathogens, differences in ORs were apparent by age group. For rotavirus, we found ORs of 6·8 (3·7–12·5) for the very low or low child mortality setting and 9·9 (6·2–15·9) for the high child mortality setting among children aged 0–4 years, but a lower OR of 2·9 (1·3–6·5) among older children and adults.

When the adjusted models were run excluding outliers (n=27), the main results were similar (appendix p 37). Additional sensitivity analyses, including adjusted models excluding RRs (ie, using only OR effect estimates; appendix p 38) and excluding co-infections (appendix p 39), did not substantially impact the results. When the adjusted models were fit without making assumptions about the 2-year and 5-year age maximums, moderate differences were observed for the summary ORs (appendix p 40). When the models were fit to data from high child mortality settings stratified by African, and south Asian and southeast Asian regions, modest differences were observed across the two regions (appendix p 42).

## Discussion

In this systematic review and meta-analysis, we estimated the strength of association between pathogen detection in stool and diarrhoea for 15 pathogens stratified by age group and child mortality setting. All enteropathogens showed an association with disease in unstratified and unadjusted analyses except for *G lamblia*. Summary ORs were generally accompanied by wide CIs, reflecting the considerable heterogeneity among studies. ORs typically differed by age, child mortality status, or both, although not in a systematic way that was consistent with an overarching explanation. This analysis highlights that enteropathogens that contribute to the syndrome of diarrhoea each have their own epidemiology and immunity characteristics, and emphasises an important limitation of the GBD model that uses effect estimates from a single study among children in high mortality settings to make global attribution estimates.

The ORs estimated by age and setting might relate to epidemiological and natural history or immunity characteristics unique to each pathogen, such as frequency of exposure, asymptomatic infection, and development of immunity. For example, the lower OR among children younger than 5 years for many of the viruses in high child mortality settings compared with very low or low child mortality settings might be reflective of a higher frequency of exposure and asymptomatic infection in settings with poorer sanitation and health infrastructure.^13^, ^14^ Surprisingly, we did not consistently find higher ORs in very low or low, compared with high, child mortality settings for bacterial and parasitic enteropathogens. Some of the clearest differences in ORs occurred across age groups—eg, the strong immunity against symptomatic disease that develops with repeated rotavirus infections and age^13^, ^14^ is consistent with a substantially lower OR among older children and adults when compared with children younger than 5 years. We were unable to further stratify the group aged 5 years or older due to limited observations, potentially hiding even more striking differences between children and adults. Host characteristics might also contribute to these differing ORs, particularly across settings. Applying one OR across settings and age groups is probably not appropriate for most enteropathogens.

We found several similarities to the findings of the GEMS study, but also notable differences in the association between pathogen presence and diarrhoea. Adenovirus 40/41, rotavirus, and *Shigella* had the strongest associations with diarrhoea among children aged 0–4 years in our meta-analysis. GEMS similarly found adenovirus 40/41, rotavirus, and *Shigella* to be strongly associated with diarrhoea; however, that study also found strong associations with *Cryptosporidium* and ETEC. Astrovirus, ETEC, *Salmonella*, and *E histolytica* showed more modest associations with diarrhoea in our meta-analysis and were associated with diarrhoea in GEMS.^15^ The differences we found might be a result of differences in the settings or populations, time of study, and study protocols including diagnostics.

These results should be considered in the context of limitations. First, for all pathogens, our ORs were accompanied by wide CIs. These CIs reflect limited sample size for many stratifications (eg, *V cholerae*, *Aeromonas*, older children, adults, and strain-specific effect estimates), but also the wide range of effect estimates from the individual studies identified in the literature review. Nevertheless, these CIs reflect the wide uncertainty that should be incorporated into burden of disease models. Second, pathogen detection methods vary by study and might contribute to heterogeneity in effect estimates. The number of pathogens screened for, and differences between, these methods (eg, different sensitivities or differences in PCR cycle thresholds used to define infection^8^, ^15^) are not captured in this analysis. Relatedly, we collapsed the pathogen detection methods into categories in an effort to create reasonable sample sizes for stratified analyses. This categorisation might not be applicable across pathogens, because the assays considered to be conventional detection methods might vary. Third, detailed data on pathogen subtypes were not available from many studies. Our inability to distinguish between subtypes (some of which might be more or less pathogenic) could result in underestimation of the OR for a given enteropathogen. For example, our ORs for ST ETEC might be underestimated because the category likely encompasses additional subtypes that differ in their association with diarrhoea.^16^, ^17^, ^18^ Fourth, including results from cohort studies and unmatched case-control studies might have contributed to an age bias in our analysis. Studies that did not control for age (ie, are not age matched) might inappropriately reduce the effect estimate because carriage of pathogens generally increases with age.^15^ Finally, the OR is a simple metric that might not reflect the complex interactions of host immunity, exposure, and infection.^19^, ^20^

This systematic review and meta-analysis demonstrates the variability in associations between pathogen detection and diarrhoeal disease by population, setting, and study method, emphasising the importance of building burden of disease estimates on diverse data sources.^21^ We provide pathogen-specific ORs stratified by age and setting. Integrating these values into burden of disease models will serve to improve age-specific and location-specific burden estimates for individual pathogens. Furthermore, future research refining attributable fraction estimates for enteric disease can build on these results and incorporate pathogen prevalence.^3^, ^19^, ^20^ More representative and accurate burden of disease estimates can then be used to inform decisions and prioritisation of public health measures such as vaccine investment and policy.

## Data sharing

Proposals requesting access to the data from the systematic review of the literature can be submitted to WHO (hassoagopsowiczm@who.int). Model code from the meta-analysis is available at https://github.com/lopmanlab/enteropathogen_odds_ratios.

## Declaration of interests

VEP has received reimbursement from Merck and Pfizer for travel expenses unrelated to the topic of this Article, and is a member of the WHO Immunization and Vaccine-related Implementation Research Advisory Committee. BAL reports grants and personal fees from Takeda Pharmaceuticals. All other authors declare no competing interests.
