# Supplementary material for: Association of enteropathogen detection with diarrhoea by age and high versus low child mortality settings: a systematic review and meta-analysis
Source: Lancet Glob Health. 2021 Sep 14;9(10):e1402–10. doi: 10.1016/S2214-109X(21)00316-8 (PMC8456779; doi:10.1016/S2214-109X(21)00316-8)
Supplement: Supplementary appendix [file mmc1.pdf]

THE LANCET
Global Health

Supplementary appendix

This appendix formed part of the original submission and has been peer reviewed. We post it as supplied by the authors.

Supplement to: Baker JM, Hasso-Agopsowicz M, Pitzer VE, et al. Association of enteropathogen detection with diarrhoea by age and high versus low child mortality settings: a systematic review and meta-analysis. *Lancet Glob Health* 2021; **9:** e1402–10.

Supplemental material

Association of enteropathogen detection with diarrhea by age and high- versus low-child mortality settings: A systematic review and meta-analysis

Systematic review methods

Search string for identifying literature in PubMed:

(diarrh*[tiab] OR gastroenteritis[tiab] OR enteric infection*[tiab])
AND
(aeromonas[tiab] OR entamoeba[tiab] OR cryptosporidium[tiab] OR giardia lamblia[tiab] OR adenovir*[tiab] OR astrovir*[tiab] OR sapovirus[tiab] OR norovirus[tiab] OR rotavirus[tiab] OR Escherichia coli[tiab] OR etec[tiab] OR epec[tiab] OR E. coli[tiab] OR cholera*[tiab] OR campylobacter[tiab] OR shigell*[tiab] OR salmonell*[tiab])
AND
(Case Control[tiab] OR Cohort[tiab])
AND
1990:3000[pdat]
NOT
(animals[mh] NOT humans[mh])

Inclusion criteria:

- Studies published between Jan 1, 1990 and July 9, 2019
- Studies performed in humans
- Studies with at least one of the pathogens of interest
- Either a case-control or a cohort study with a disease outcome of "diarrhea"
- Published in English, French, Spanish, Portuguese, Italian, or Chinese

Exclusion criteria:

- Studies in which non-diarrheal controls were not reported
- Studies which included participants with a broad case definition of gastroenteritis (i.e. diarrhea *or* vomiting) in which it was not possible to ascertain that all cases had diarrhea
- Studies limited to inclusion of participants with nosocomial infections
- Studies conducted in patients with chronic, underlying, non-enteric conditions (except participants a Human Immunodeficiency Virus diagnosis)
- Studies evaluating inflammatory bowel diseases
- Abstracts and conference proceedings
- Studies in which no human biological testing was conducted
- Publication was a systematic review

Table S1. Pathogens of interest and relevant strains

Pathogen	Strains measured
Salmonella (enterica) spp.<br>Or<br>Salmonellosis	All Salmonella (enterica) spp. except Salmonella typhi
Shigella spp.<br>Or<br>Shigellosis	All
Campylobacter spp.<br>Or<br>Campylobacter enteritis	All
Vibrio cholerae<br>Or<br>Cholera	O1<br>O139
Enterotoxigenic *Escherichia coli*<br>(ETEC)	LT-ETEC (heat-labile enterotoxigenic *E. coli)*<br>ST-ETEC (heat-stable enterotoxigenic *E. coli)*
Enteropathogenic *Escherichia coli (EPEC)*<br>Or<br>Typical enteropathogenic *Escherichia coli*<br>(tEPEC)	All
Rotavirus<br>Or<br>Rotavirus gastroenteritis (RVGE)	All
Calicivirus (Norovirus)	Group I<br>Group II
Calicivirus (Sapovirus)	All
Astrovirus	All
Enteric adenovirus	40<br>41
Giardia lamblia	All
Cryptosporidium spp.	All
Entamoeba histolytica (amoebiasis)	All
Aeromonas spp	All

Data collection process:

Titles and abstracts were screened according to the inclusion and exclusion criteria by two independent reviewers. Any conflict between reviewers were resolved by reaching an agreement through discussion; if the difference persisted, the study was passed through to full-text review. At the full-text review stage, two independent reviewers also conducted screening, and conflicts were once again resolved through discussion. If a conflict persisted, the decision of inclusion was made by the senior faculty member. The abstract and full-text review was completed in *Covidence* Software (available at https://www.covidence.org).

Data extraction was done by a single reviewer per study using *REDCap* (available at https://www.project-redcap.org/). A random sample of 20 manuscripts targeted for data extraction were also extracted by a second reviewer to assess for errors. All questions relevant to data extraction were discussed during a weekly team meeting.

Quality control review:

Following the data extraction process, we completed a quality control review of 10% of all studies extracted (n=17). The quality control process assessed 1) the accuracy of data entered into the REDCap database and 2) whether any data entry errors contributed to risk measure calculations. Researchers reviewed studies originally extracted by team members other than themselves. Following quality control review, the team determined data entry errors were below the predetermined established threshold of 5%.

The protocol for this systematic review has not been published.

Table S2. Description of the 130 studies identified in the systematic literature review and supplemental data used in the analysis.

Primary author	Year of publication	Article title	Study design	Validity score[a]	Cases/ diarrhea samples (n)	Controls /non-diarrheal samples (n)	Country	Child mortality status category
Aboderin[1]	2012	Role of Campylobacter jejuni/coli in diarrhoea in Ile-Ife, Nigeria	Case-control	3	303	100	Nigeria	high
Abu-Elyazeed[2]	1999	Epidemiology of Enterotoxigenic Escherichia coli Diarrhea in a Pediatric Cohort in a Periurban Area of Lower Egypt	Nested case-control	4	477	1537	Egypt	high
Acosta[3]	2016	Diarrheagenic Escherichia coli: Prevalence and Pathotype Distribution in Children from Peruvian Rural Communities	Nested case-control	4	46	774	Peru	very low/low
Afset[4]	2004	Association of atypical enteropathogenic Escherichia coli (EPEC) with prolonged diarrhoea	Case-control	3	251	210	Norway	very low/low
Albert[5]	1999	Case-Control Study of Enteropathogens Associated with Childhood Diarrhea in Dhaka, Bangladesh	Case-control	4	814	814	Bangladesh	high
Aminu[6]	2010	Diversity of Rotavirus VP7 and VP4 Genotypes in Northwestern Nigeria	Case-control	2	869	194	Nigeria	high
Apelt[7]	2010	The Prevalence of Norovirus in returning international travelers with diarrhea	Case-control	3	157	48	Germany	very low/low
Arthur[8]	2009	A Novel Bocavirus Associated with Acute Gastroenteritis in Australian Children	Case-control	4	197	197	Australia	very low/low
Ashie[9]	2017	Microbial pathogens associated with acute childhood diarrhoea in Kumasi, Ghana	Case-control	5	240	107	Ghana	high
Ballard[10]	2015	Epidemiology and Genetic Characterization of Noroviruses among Adults in an Endemic Setting, Peruvian Amazon Basin, 2004-2011	Nested case-control	4	200	200	Peru	very low/low
Becker[11]	2015	Combined stool-based multiplex PCR and microscopy for enhanced pathogen detection in patients with persistent diarrhoea and asymptomatic controls from C√¥te d'Ivoire	Case-control	5	68	68	C√¥te d'Ivoire	high
Becker-Dreps[12]	2014	Etiology of Childhood Diarrhea Following Rotavirus Vaccine Introduction: A Prospective, Population-Based Study in Nicaragua	Prospective cohort	5	337	106	Nicaragua	very low/low
Bhandari[13]	1999	Role of protozoa as risk factors for persistent diarrhea	Case-control	4	175	175	India	high
Bodhidatta[14]	2010	Case-Control Study of Diarrheal Disease Etiology in a Remote Rural Area in Western Thailand	Case-control	3	236	236	Thailand	very low/low
Bodhidatta[15]	2019	Epidemiology and etiology of Traveler's diarrhea in Bangkok, Thailand, a case-control study	Case-control	5	389	400	Thailand	very low/low
Bona[16]	2019	Virulence-related genes are associated with clinical and nutritional outcomes of Shigella/Enteroinvasive Escherichia coli pathotype infection in children from Brazilian semiarid region: A community case-control study	Case-control	4	596	604	Brazil	very low/low
Boru[17]	2013	Aetiology and factors associated with bacterial diarrhoeal diseases amongst urban refugee children in Eastleigh, Kenya: A case control study	Case-control	4	41	41	Kenya	high
Bravo[18]	2012	Aeromonas spp asociada a enfermedad diarreica aguda en Cuba: estudios de casos y controles	Case-control	3	2322	2072	Cuba	very low/low
Breurec[19]	2016	Etiology and Epidemiology of Diarrhea in Hospitalized Children from Low Income Country: A Matched Case-Control Study in Central African Republic	Case-control	4	333	333	Central African Republic	high

Brink[20]	2002	Diarrhea, CD4 counts and enteric infections in a community-based cohort of HIV-infected adults in Uganda	Prospective cohort	4	357	127	Uganda	high
Bruijnesteijn van Coppenraet[21]	2015	Case-control comparison of bacterial and protozoan microorganisms associated with gastroenteritis: application of molecular detection	Case-control	3	1515	1195	The Netherlands	very low/low
Brunser[22]	1992	Field trial of an infant formula containing anti-rotavirus and anti-Escherichia coli milk antibodies from hyperimmunized cows	Randomized controlled trial	4	NA	NA	Chile	very low/low
Bueris[23]	2007	Detection of diarrheagenic Escherichia coli from children with and without diarrhea in Salvador, Bahia, Brazil	Nested case-control	5	1020	187	Brazil	very low/low
Carcamo[24]	2005	Etiologies and Manifestations of Persistent Diarrhea in Adults with HIV-1 Infection: A Case-Control Study in Lima, Peru	Case-control	5	147	147	Peru	very low/low
Cardemil[25]	2017	Pathogen-Specific Burden of Outpatient Diarrhea in Infants in Nepal: A Multisite Prospective Case-Control Study	Nested case-control	5	307	358	Nepal	high
Cennimo[26]	2009	The prevalence and virulence characteristics of enteroaggregative Escherichia coli at an urgent care clinic in the USA: a case-control study	Case-control	4	253	751	United States	very low/low
Chang[27]	2017	A Hospital-based Case-control Study of Diarrhea in Children in Shanghai	Case-control	4	680	680	China	very low/low
Chhin[28]	2006	Etiology of Chronic Diarrhea in Antiretroviral-Naive Patients with HIV Infection Admitted to Norodom Sihanouk Hospital, Phnom Penh, Cambodia	Case-control	4	40	40	Cambodia	high
Clemens[29]	2004	Development of Pathogenicity-Driven Definitions of Outcomes for a Field Trial of a Killed Oral Vaccine against Enterotoxigenic Escherichia coli in Egypt: Application of an Evidence-Based Method	Prospective cohort	4	2201	1881	Egypt	high
Cohen[30]	2012	An Inverse and Independent Association Between Helicobacter pylori Infection and the Incidence of Shigellosis and Other Diarrheal Diseases	Nested case-control	3	177	418	Israel	very low/low
Contreras[31]	2010	Allelic variability of critical virulence genes (eae, bfpA and perA) in typical and atypical enteropathogenic Escherichia coli in Peruvian children	Case-control	2	936	424	Peru	very low/low
Cranendonk[32]	2003	Cryptosporidium parvum and Isospora belli infections among patients with and without diarrhoea	Case-control	4	121	122	Malawi	high
da Silva Quetz[33]	2010	Campylobacter jejuni and Campylobacter coli in children from communities in Northeastern Brazil: molecular detection and relation to nutritional status	Case-control	3	83	242	Brazil	very low/low
de Wit[34]	2001	Etiology of Gastroenteritis in Sentinel General Practices in The Netherlands	Case-control	4	857	574	The Netherlands	very low/low
Denno[35]	2012	Diarrhea Etiology in a Pediatric Emergency Department: A Case Control Stud	Case-control	4	254	452	United States	very low/low
Do[36]	2007	Epidemiology and aetiology of diarrhoeal diseases in adults engaged in wastewater-fed agriculture and aquaculture in Hanoi, Vietnam	Nested case-control	5	163	163	Vietnam	very low/low
Dutta[37]	1990	Epidemiology of Rotavirus Diarrhoea in Children under Five Years in Bahrain	Case-control	4	698	532	Bahrain	very low/low
Dwivedi[38]	2007	Enteric Opportunistic Parasites among HIV Infected Individuals: Associated Risk Factors and Immune Status	Case-control	5	50	25	India	high

Eibach[39]	2016	Application of a multiplex PCR assay for the detection of gastrointestinal pathogens in a rural African setting	Case-control	4	443	239	Ghana	high
El-Hakim[40]	1996	Association of parasites and diarrhoea among children less than five years of age in a rural area in Egypt	Case-control	1	196	83	Egypt	high
El-Shabrawi[41]	2015	The burden of different pathogens in acute diarrhoeal episodes among a cohort of Egyptian children less than five years old	Case-control	4	356	356	Egypt	high
Espinoza[42]	1997	Rotavirus infections in young Nicaraguan children	Prospective cohort	4	435	887	Nicaragua	very low/low
Fang[43]	1995	Etiology and Epidemiology of Persistent Diarrhea in Northeastern Brazil: A Hospital-Based, Prospective, Case-Control Study	Case-control	4	52	42	Brazil	very low/low
Farfan-Garcia[44]	2017	Case-Control Pilot Study on Acute Diarrheal Disease in a Geographically Defined Pediatric Population in a Middle Income Country	Case-control	5	45	45	Colombia	very low/low
Fathy[45]	2014	Molecular Copro-prevalence of Cryptosporidium in Egyptian Children and Evaluation of Three Diagnostic Methods	Cross-sectional	3	NA	NA	Egypt	high
Firdu[46]	2014	Intestinal Protozoal Parasites in Diarrheal Children and Associated Risk Factors at Yirgalem Hospital, Ethiopia: A Case-Control Study	Case-control	3	115	115	Ethiopia	high
Francois[47]	2018	The other Campylobacters: Not innocent bystanders in endemic diarrhea and dysentery in children in low-income settings	Case-control	5	297	198	Peru	very low/low
Fraser[48]	1997	Natural history of Giardia lamblia and Cryptosporidium infections in a cohort of Israeli Bedouin infants: a study of a population in transition	Prospective cohort	3	272	781	Israel	very low/low
Fraser[49]	1998	Persistent Diarrhea in a Cohort of Israeli Bedouin Infants: Role of Enteric Pathogens and Family and Environmental Factors	Prospective cohort	4	190	1262	Israel	very low/low
Gascon[50]	1998	Enteroaggregative Escherichia coli Strains as a Cause of Traveler's Diarrhea: A Case-Control Study	Case-control	3	165	165	Spain	very low/low
Gascon[51]	2000	Diarrhea in Children under 5 Years of Age from Ifakara, Tanzania: a Case-Control Study	Case-control	4	103	206	Tanzania	high
Gassama[52]	2001	Ordinary and Opportunistic Enteropathogens Associated with Diarrhea in Senegalese Adults in Relation to Human Immunodeficiency Virus Serostatus	Case-control	2	121	155	Senegal	high
Georges-Courbot[53]	1990	A cohort study of enteric campylobacter infection in children from birth to two years in Bangui (Central African Republic)	Prospective cohort	3	349	5445	Central African Republic	high
Gomez-Duarte[54]	2013	Enterotoxigenic Escherichia coli associated with childhood diarrhoea in Colombia, South America	Nested case-control	4	466	349	Colombia	very low/low
Haque[55]	2009	Prospective Case-Control Study of the Association between Common Enteric Protozoal Parasites and Diarrhea in Bangladesh	Case-control	4	3646	2575	Bangladesh	high
Hien[56]	2007	Diarrhoeagenic Escherichia coli and other causes of childhood diarrhoea: a case-control study in children living in a wastewater-use area in Hanoi, Vietnam	Case-control	4	111	111	Vietnam	very low/low
Hien[57]	2008	Diarrheagenic Escherichia coli and Shigella Strains Isolated from Children in a Hospital Case-Control Study in Hanoi, Vietnam	Case-control	3	249	124	Vietnam	very low/low

Hoge[58]	1996	Epidemiology of Diarrhea Among Expatriate Residents Living in a Highly Endemic Environment	Case-control	4	189	112	Nepal	high
Holtz[59]	2011	Astrovirus MLB1 Is Not Associated with Diarrhea in a Cohort of Indian Children	Nested case-control	3	400	400	India	high
Isenbarger[60]	2001	Prospective study of the incidence of diarrhoea and prevalence of bacterial pathogens in a cohort of Vietnamese children along the Red River	Prospective cohort	4	2160	203	Vietnam	very low/low
Iturriza-Gomara[61]	2019	Etiology of Diarrhea Among Hospitalized Children in Blantyre, Malawi, Following Rotavirus Vaccine Introduction: A Case-Control Study	Case-control	4	684	527	Malawi	high
Jain[62]	2015	Campylobacter species and drug resistance in a north Indian rural community	Case-control	2	348	351	India	high
Krumkamp[63]	2015	Gastrointestinal Infections and Diarrheal Disease in Ghanaian Infants and Children: An Outpatient Case-Control Study	Case-control	3	548	686	Ghana	high
Lee[64]	2013	Symptomatic and Asymptomatic Campylobacter Infections Associated with Reduced Growth in Peruvian Children	Prospective cohort	3	3973	3271	Peru	very low/low
Levine[65]	1993	Epidemiologic Studies of Escherichia coli Diarrheal Infections in a Low Socioeconomic Level Peri-Urban Community In Santiago, Chile	Prospective cohort	4	1081	1047	Chile	very low/low
Li[66]	2015	Aetiology of diarrhoeal disease and evaluation of viral-bacterial coinfection in children under 5 years old in China: a matched case-control study	Case-control	3	461	461	China	very low/low
Lima[67]	2019	Etiology and severity of diarrheal diseases in infants at the semiarid region of Brazil: A case-control study	Case-control	3	596	604	Brazil	very low/low
Liu[68]	2016	Etiological Role and Repeated Infections of Sapovirus among Children Aged Less than 2 Years in a Cohort Study in a Peri-urban Community of Peru	Nested case-control	3	299	399	Peru	very low/low
Lule[69]	2009	Aetiology of diarrhoea among persons with HIV and their family members in rural Uganda: a community-based study	Prospective cohort	2	436	562	Uganda	high
Maldonado[70]	1998	Population-Based Prevalence of Symptomatic and Asymptomatic Astrovirus Infection in Rural Mayan Infants	Prospective cohort	1	305	1949	Mexico	very low/low
Mansour[71]	2012	Burden of Aeromonas hydrophila -associated diarrhea among children younger than 2 years in rural Egyptian community	Prospective cohort	3	4001	9539	Egypt	high
Mansour[72]	2013	Modifiable diarrhoea risk factors in Egyptian children aged <5 years	Case-control	4	400	400	Egypt	high
Mansour[73]	2014	Diarrhea Burden Due to Natural Infection with Enterotoxigenic Escherichia coli in a Birth Cohort in a Rural Egyptian Community	Prospective cohort	3	4001	9539	Egypt	high
Mansour[74]	2014	Pathogenicity and Phenotypic Characterization of Enterotoxigenic Escherichia coli Isolates from a Birth Cohort of Children in Rural Egypt	Nested case-control	3	4001	9539	Egypt	high
Mason[75]	2017	Antibiotic resistance in Campylobacter and other diarrheal pathogens isolated from US military personnel deployed to Thailand in 2002-2004: a case-control study	Case-control	3	155	62	Thailand	very low/low
Megraud[76]	1990	Incidence of Campylobacter infection in infants in western Algeria and the possible protective role of breast feeding	Case-control	4	411	247	Algeria	very low/low

Mercado[77]	2010	Fecal Leukocytes in Children Infected with Diarrheagenic Escherichia coli	Nested case-control	4	935	539	Peru	very low/low
Ming[78]	1991	Diarrhoeal disease in children less than one year of age at a children's hospital in Guangzhou, People's Republic of China	Case-control	3	174	174	People's Republic of China	very low/low
Mitra[79]	2016	Enteric Parasitic Infection Among Antiretroviral Therapy Naive HIV-Seropositive People: Infection Begets Infection-Experience from Eastern India	Case-control	3	194	98	India	high
Mota-Hernandez[80]	2003	Rotavirus Diarrhea Severity Is Related to the VP4 Type in Mexican Children	Case-control	4	520	520	Mexico	very low/low
Moyo[81]	2014	Prevalence and molecular characterisation of human adenovirus in diarrhoeic children in Tanzania; a case control study	Case-control	4	690	545	Tanzania	high
Muhsen[82]	2014	Can Giardia lamblia Infection Lower the Risk of Acute Diarrhea among Preschool Children?	Prospective cohort	3	33	109	Israel	very low/low
Mullick[83]	2014	Community Based Case-Control Study of Rotavirus Gastroenteritis among Young Children during 2008-2010 Reveals Vast Genetic Diversity and Increased Prevalence of G9 Strains in Kolkata	Retrospective cohort	3	1568	2014	India	high
My[84]	2013	Endemic Norovirus Infections in Children, Ho Chi Minh City, Vietnam, 2009-2010	Case-control	3	1419	609	Vietnam	very low/low
Nelson[85]	2018	Genetic Diversity of Noroviruses Circulating in a Pediatric Cohort in Bangladesh	Case-control	4	1641	553	Bangladesh	high
Newman[86]	1999	Longitudinal Study of Cryptosporidium Infection in Children in Northeastern Brazil	Prospective cohort	3	97	299	Brazil	very low/low
Newman[87]	2001	A longitudinal study of Giardia lamblia infection in north-east Brazilian children	Prospective cohort	4	97	299	Brazil	very low/low
Nimri[88]	1994	Cryptosporidium. A cause of gastroenteritis in preschool children in Jordan	Case-control	3	300	300	Jordan	very low/low
Nimri[89]	1996	Rotavirus-associated Diarrhoea in Children in a Refugee Camp in Jordan	Case-control	3	220	200	Jordan	very low/low
O'Ryan[90]	2009	Symptomatic and Asymptomatic Rotavirus and Norovirus Infections During Infancy in a Chilean Birth Cohort	Prospective cohort	3	145	2278	Chile	very low/low
Ochoa[91]	2009	High frequency of antimicrobial resistance of diarrheagenic E. coli in Peruvian infants	Prospective cohort	2	557	195	Peru	very low/low
Oketcho[92]	2012	Influence of enteric bacteria, parasite infections and nutritional status on diarrhoea occurrence among 6-60 months old children admitted at a Regional Hospital in Morogoro, Tanzania	Case-control	2	151	152	Tanzania	high
Olesen[93]	2005	Etiology of Diarrhea in Young Children in Denmark: a Case-Control Study	Case-control	3	424	866	Denmark	very low/low
Pandey[94]	2011	Travelers' Diarrhea in Nepal: An Update on the Pathogens and Antibiotic Resistance	Case-control	2	381	176	Nepal	high
Paniagua[95]	1997	Analysis of Incidence of Infection with Enterotoxigenic Escherichia coli in a Prospective Cohort Study of Infant Diarrhea in Nicaragua	Prospective cohort	4	808	1472	Nicaragua	very low/low
Pavie[96]	2012	Prevalence of opportunistic intestinal parasitic infections among HIV-infected patients with low CD4 cells counts in France in the combination antiretroviral therapy era	Prospective cohort	1	73	58	France	very low/low

Pazzaglia[97]	1991	Campylobacter diarrhoea and an association of recent disease with asymptomatic shedding in Egyptian children	Case-control	2	143	132	Egypt	high
Platts-Mills [b 98]	2007[c]	GEMS	Case-control	NA	291	291	Bangladesh	high
Platts-Mills [b 98]	2007[c]	GEMS	Case-control	NA	275	275	India	high
Platts-Mills [b 98]	2007[c]	GEMS	Case-control	NA	267	267	Kenya	high
Platts-Mills [b 98]	2007[c]	GEMS	Case-control	NA	274	274	Mali	high
Platts-Mills [b 98]	2007[c]	GEMS	Case-control	NA	247	247	Mozambique	high
Platts-Mills [b 98]	2007[c]	GEMS	Case-control	NA	284	284	Pakistan	high
Platts-Mills [b 98]	2007[c]	GEMS	Case-control	NA	258	258	The Gambia	high
Platts-Mills [b 99]	2009[c]	MAL-ED	Nested case-control	NA	1236	1236	Bangladesh	high
Platts-Mills [b 99]	2009[c]	MAL-ED	Nested case-control	NA	75	75	Brazil	very low/low
Platts-Mills [b 99]	2009[c]	MAL-ED	Nested case-control	NA	574	574	India	high
Platts-Mills [b 99]	2009[c]	MAL-ED	Nested case-control	NA	841	841	Nepal	high
Platts-Mills [b 99]	2009[c]	MAL-ED	Nested case-control	NA	1260	1260	Pakistan	high
Platts-Mills [b 99]	2009[c]	MAL-ED	Nested case-control	NA	1428	1428	Peru	very low/low
Platts-Mills [b 99]	2009[c]	MAL-ED	Nested case-control	NA	96	96	South Africa	high
Platts-Mills [b 99]	2009[c]	MAL-ED	Nested case-control	NA	136	136	Tanzania	high
Qadri[100]	2007	Disease Burden Due to Enterotoxigenic Escherichia coli in the First 2 Years of Life in an Urban Community in Bangladesh	Prospective cohort	1	1234	6383	Bangladesh	high
Randremanana[101]	2012	Case-Control Study of the Etiology of Infant Diarrheal Disease in 14 Districts in Madagascar	Case-control	4	2196	496	Madagascar	high
Randremanana[102]	2014	Campylobacter infection in a cohort of rural children in Moramanga, Madagascar	Prospective cohort	3	459	2965	Madagascar	high
Randremanana[103]	2016	Etiologies, Risk Factors and Impact of Severe Diarrhea in the Under-Fives in Moramanga and Antananarivo, Madagascar	Case-control	4	199	199	Madagascar	high
Rao[104]	2001	Pathogenicity and Convalescent Excretion of Campylobacter in Rural Egyptian Children	Nested case-control	3	1087	935	Egypt	high
Reyes[105]	2009	Diversity of intestinal Escherichia coli populations in Nicaraguan children with and without diarrhoea	Case-control	3	381	145	Nicaragua	very low/low
Saito[106]	2014	Multiple norovirus infections in a birth cohort in a Peruvian Periurban community	Prospective cohort	3	1495	3690	Peru	very low/low
Sallon[107]	1990	Cryptosporidiosis in children in Gaza	Case-control	4	145	67	Palestinian territory	very low/low
Scaletsky[108]	1999	Associcao de padroes de adesao de Escherichia coli as celulas Hep-2 com diarreia aguda e persistente	Case-control	3	24	34	Brazil	very low/low
Scaletsky[109]	2002	Diffusely Adherent Escherichia coli as a Cause of Acute Diarrhea in Young Children in Northeast Brazil: a Case-Control Study	Case-control	3	237	231	Brazil	very low/low
Schorling[110]	1990	A prospective study of persistent diarrhea among children in an urban Brazilian slum	Prospective cohort	4	50	38	Brazil	very low/low
Schultsz[111]	2000	Diarrheagenic Escherichia coli and Acute and Persistent Diarrhea in Returned Travelers	Case-control	4	52	109	The Netherlands	very low/low

Serichantalergs[112]	2017	Incidence of Campylobacter concisus and C. ureolyticus in traveler's diarrhea cases and asymptomatic controls in Nepal and Thailand	Case-control	3	83	75	Nepal	high
Serichantalergs[112]	2017	Incidence of Campylobacter concisus and C. ureolyticus in traveler's diarrhea cases and asymptomatic controls in Nepal and Thailand	Case-control	3	173	165	Thailand	very low/low
Shen[113]	2019	A case control study on the prevalence of enterovirus in children samples and its association with diarrhea	Case-control	2	273	361	China	very low/low
Soltan Dallal[114]	2004	Aeromonas spp associated with children's diarrhoea in Tehran: a case-control study	Case-control	2	310	310	Iran	very low/low
Sow[115]	2018	Acquisition of enteric pathogens by pilgrims during the 2016 Hajj pilgrimage: A prospective cohort study	Prospective cohort	4	15	97	Saudi Arabia	very low/low
Steiner[116]	2018	Species of Cryptosporidia Causing Subclinical Infection Associated With Growth Faltering in Rural and Urban Bangladesh: A Birth Cohort Study	Prospective cohort	3	1514	11540	India	high
Steinsland[117]	2002	Enterotoxigenic Escherichia coli Infections and Diarrhea in a Cohort of Young Children in Guinea-Bissau	Prospective cohort	3	1154	10068	Guinea-Bissau	high
Swierczewski[118]	2012	Surveillance for enteric pathogens in a case-control study of acute diarrhea in Western Kenya	Case-control	3	239	239	Kenya	high
Taniuchi[119]	2013	Etiology of Diarrhea in Bangladeshi Infants in the First Year of Life Analyzed Using Molecular Methods	Prospective cohort	5	420	1385	Bangladesh	high
Tellevik[120]	2015	Prevalence of Cryptosporidium parvum/ hominis, Entamoeba histolytica and Giardia lamblia among Young Children with and without Diarrhea in Dar es Salaam, Tanzania	Case-control	4	701	558	Tanzania	high
Tumwine[121]	2003	Cryptosporidium parvum in children with diarrhea in Mulago Hospital, Kampala, Uganda	Case-control	4	1779	667	Uganda	high
Valentiner-Branth[122]	2003	Cohort Study of Guinean Children: Incidence, Pathogenicity, Conferred Protection, and Attributable Risk for Enteropathogens during the First 2 Years of Life	Prospective cohort	4	NA	NA	Guinea-Bissau	high
Vasco[123]	2014	Identifying Etiological Agents Causing Diarrhea in Low Income Ecuadorian Communities	Case-control	3	100	100	Ecuador	very low/low
Vernacchio[124]	2006	Characteristics of Persistent Diarrhea in a Community-Based Cohort of Young US Children	Prospective cohort	4	50	485	United States	very low/low
Vernacchio[125]	2006	Diarrhea in American Infants and Young Children in the Community Setting: Incidence, Clinical Presentation and Microbiology	Prospective cohort	3	447	485	United States	very low/low
Vethanayagam[126]	2004	Possible Role of Neonatal Infection with the Asymptomatic Reassortant Rotavirus (RV) Strain I321 in the Decrease in Hospital Admissions for RV Diarrhea, Bangalore, India, 1988-1999	Prospective cohort	3	13	59	India	high
Viboud[127]	1999	Prospective Cohort Study of Enterotoxigenic Escherichia coli Infections in Argentinean Children	Prospective cohort	3	68	79	Argentina	very low/low
Vieira[128]	2007	HIGH PREVALENCE OF ENTEROINVASIVE ESCHERICHIA COLI ISOLATED IN A REMOTE REGION OF NORTHERN COASTAL ECUADOR	Case-control	3	236	679	Ecuador	very low/low
Yori[129]	2009	Norovirus highly prevalent cause of endemic acute diarrhea in children in the Peruvian Amazon	Nested case-control	1	263	75	Peru	very low/low

Zhang[130]	2016	Case-control study of diarrheal disease etiology in individuals over 5 years in southwest China	Case-control	3	271	149	China	very low/low

[a] Validity score calculated by awarding 1 point for each of the following criteria (maximum score of 5): 1) case/outcome-positive definition provided, 2) control/outcome-negative definition provided, 3) diarrhea presentation defined, 4) lab certification or quality framework described, and 5) diarrhea definition provided.

[b] Data provided by James Platts-Mills.

[c] Year the study started (not publication date)

Figure S1. Adenovirus 40/41 forest plot

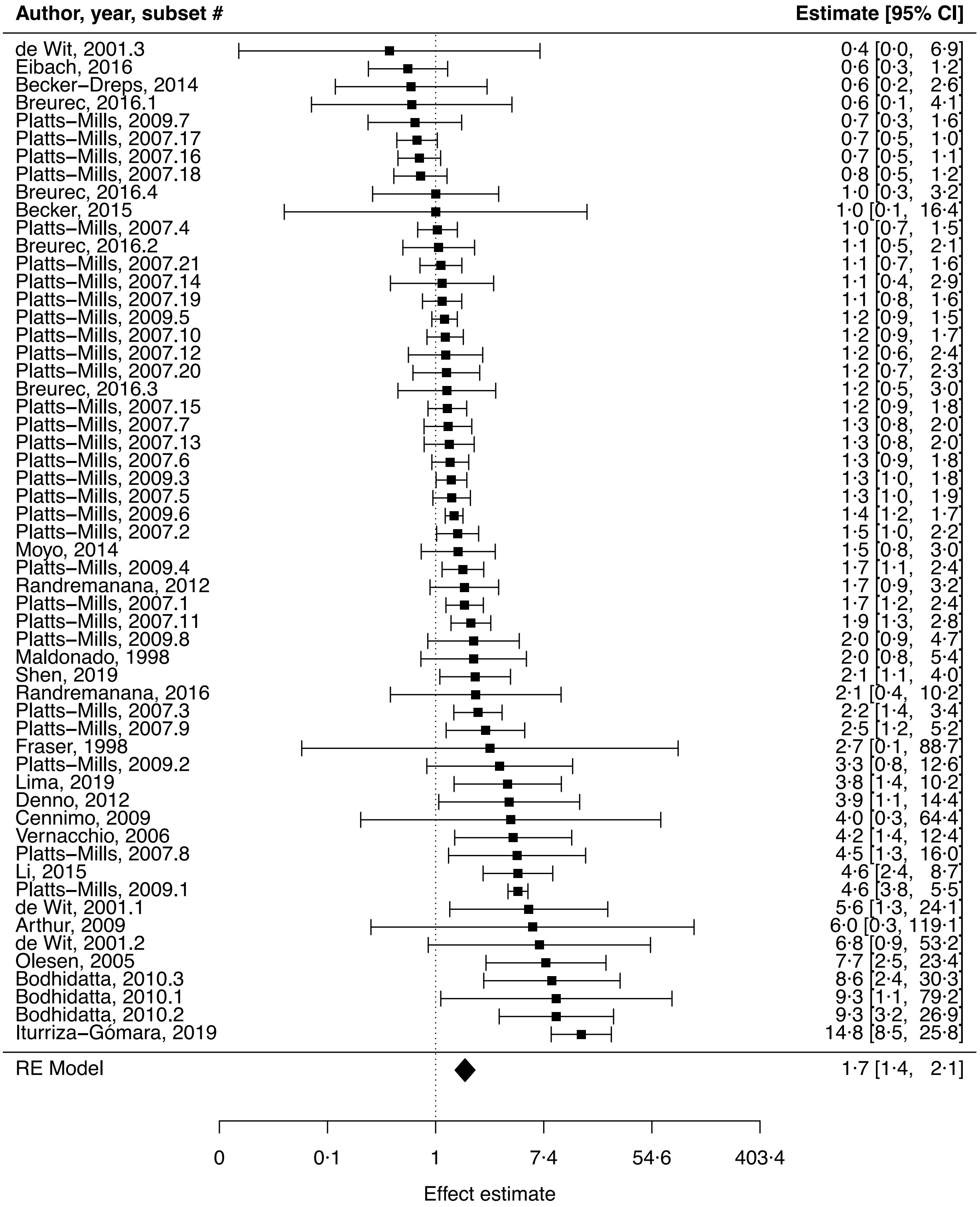

Figure S2. Astrovirus forest plot

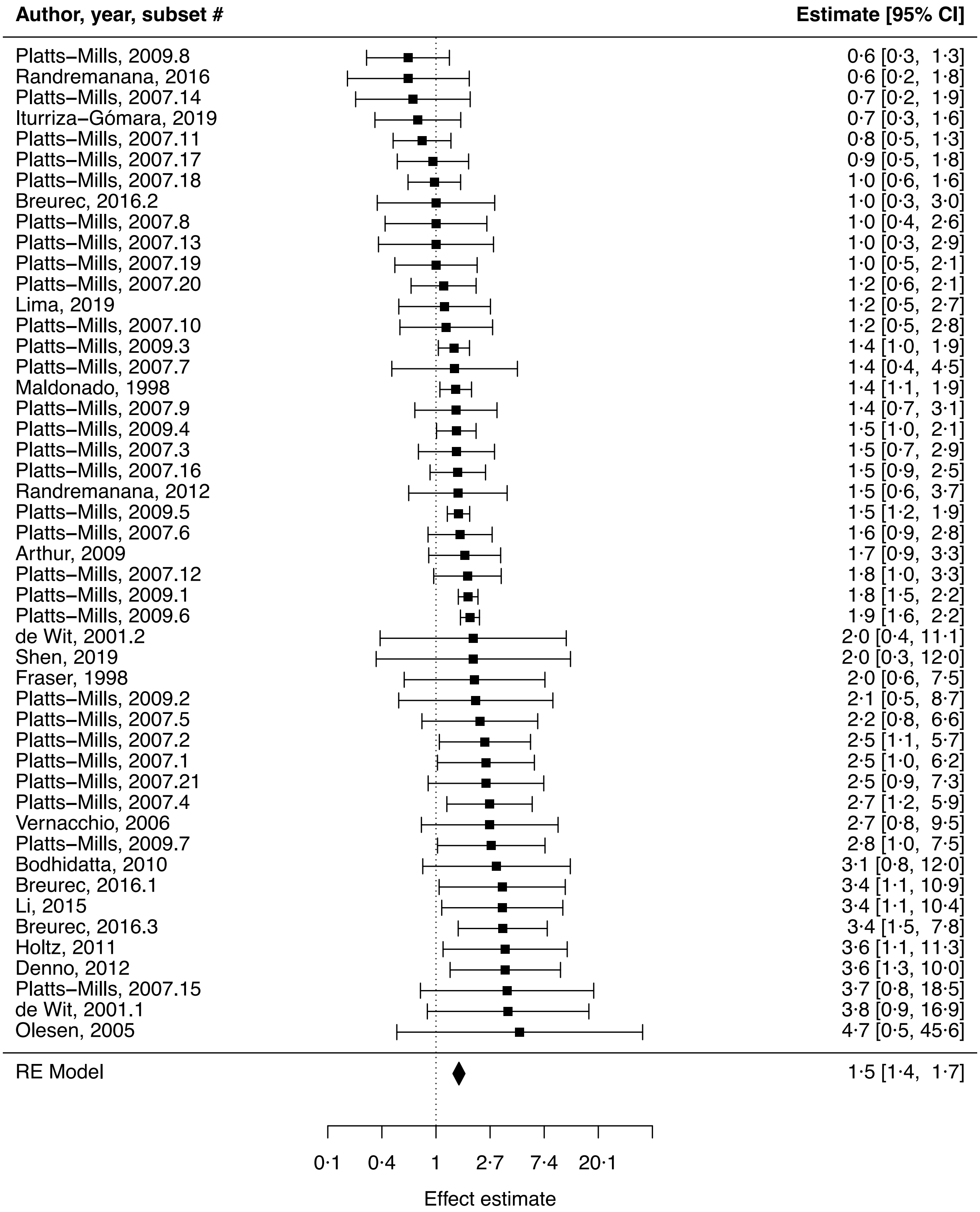

Figure S3. Norovirus forest plot

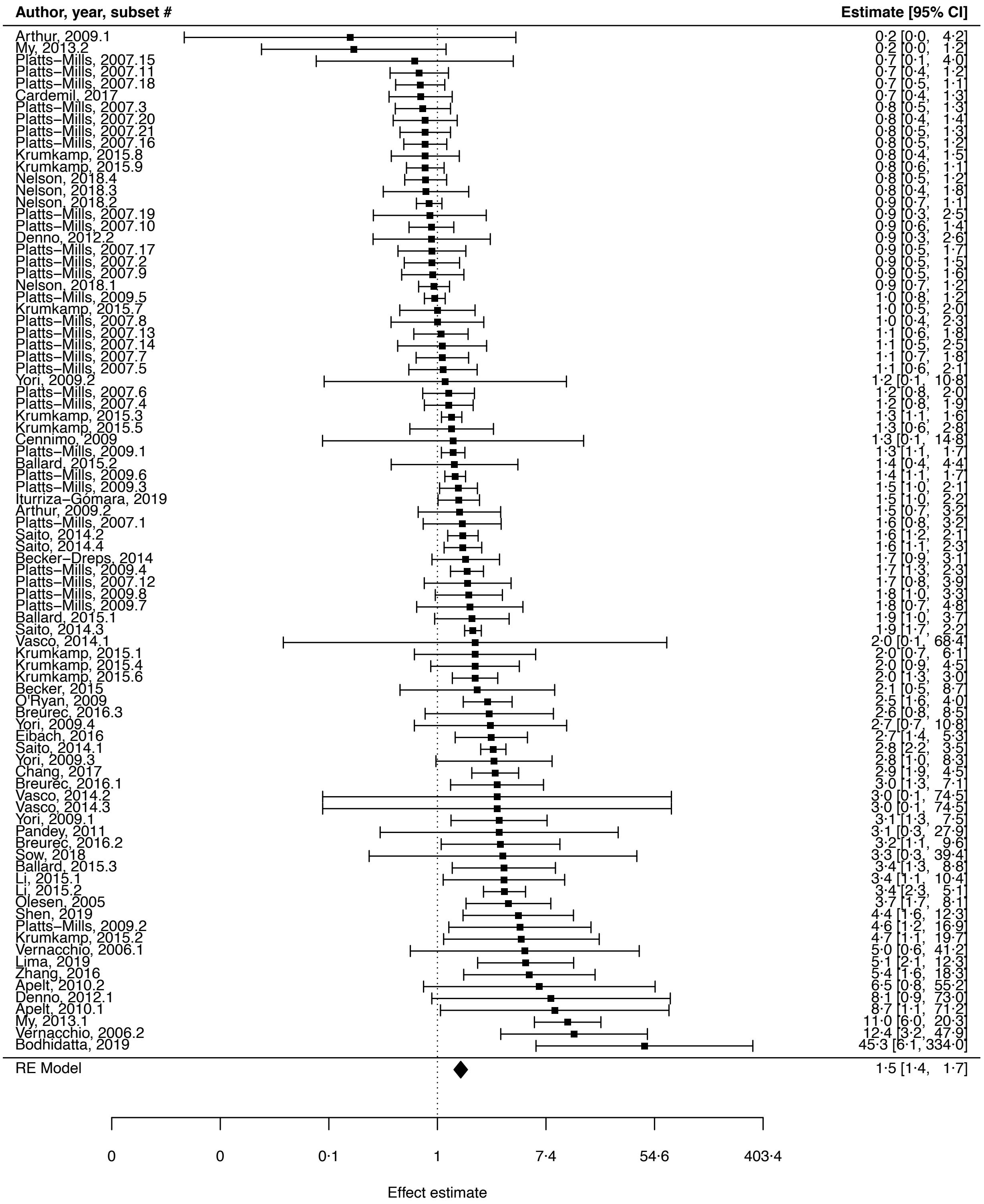

Figure S4. Rotavirus forest plot

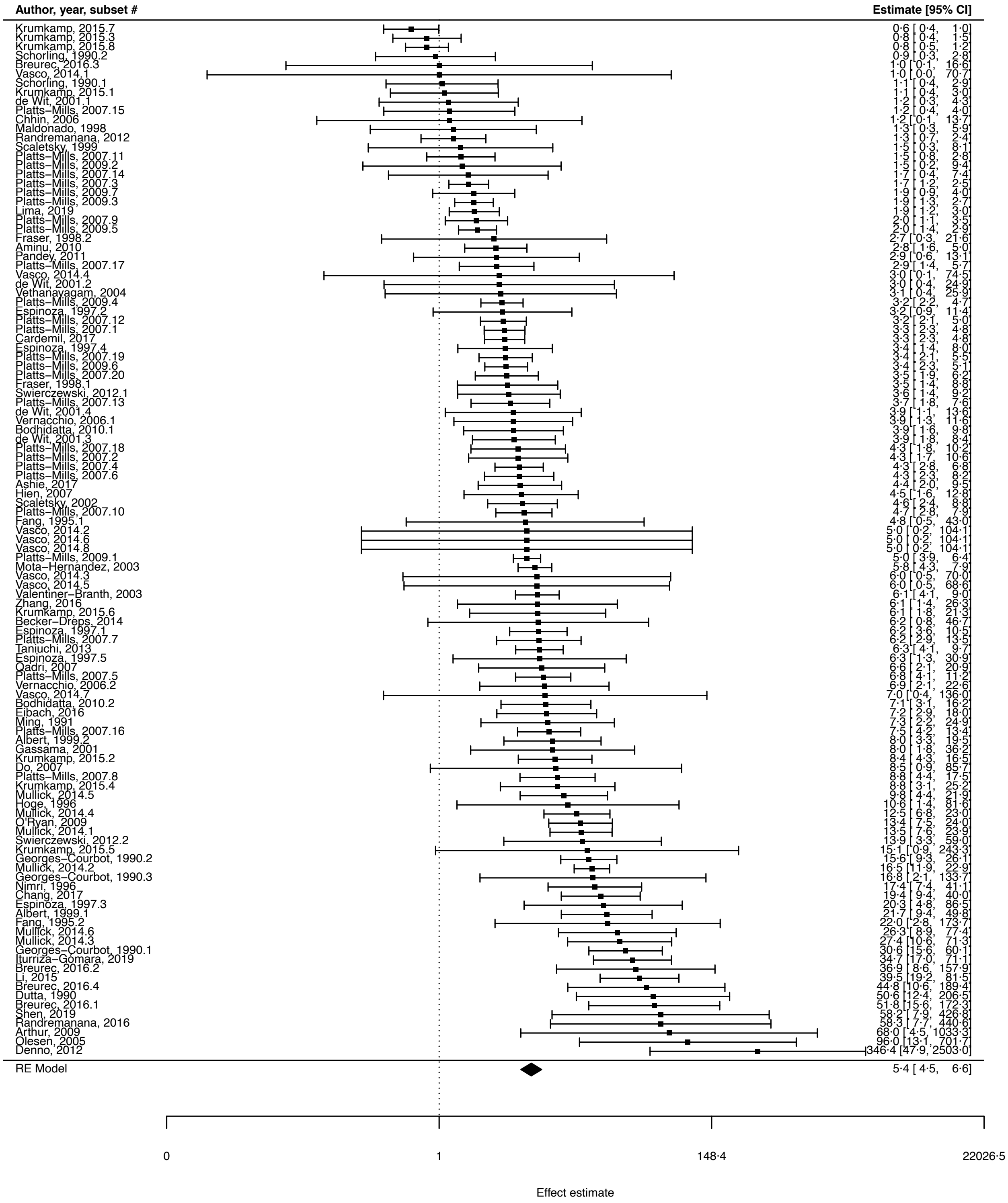

Figure S5. Rotavirus (pre-vaccine introduction) forest plot

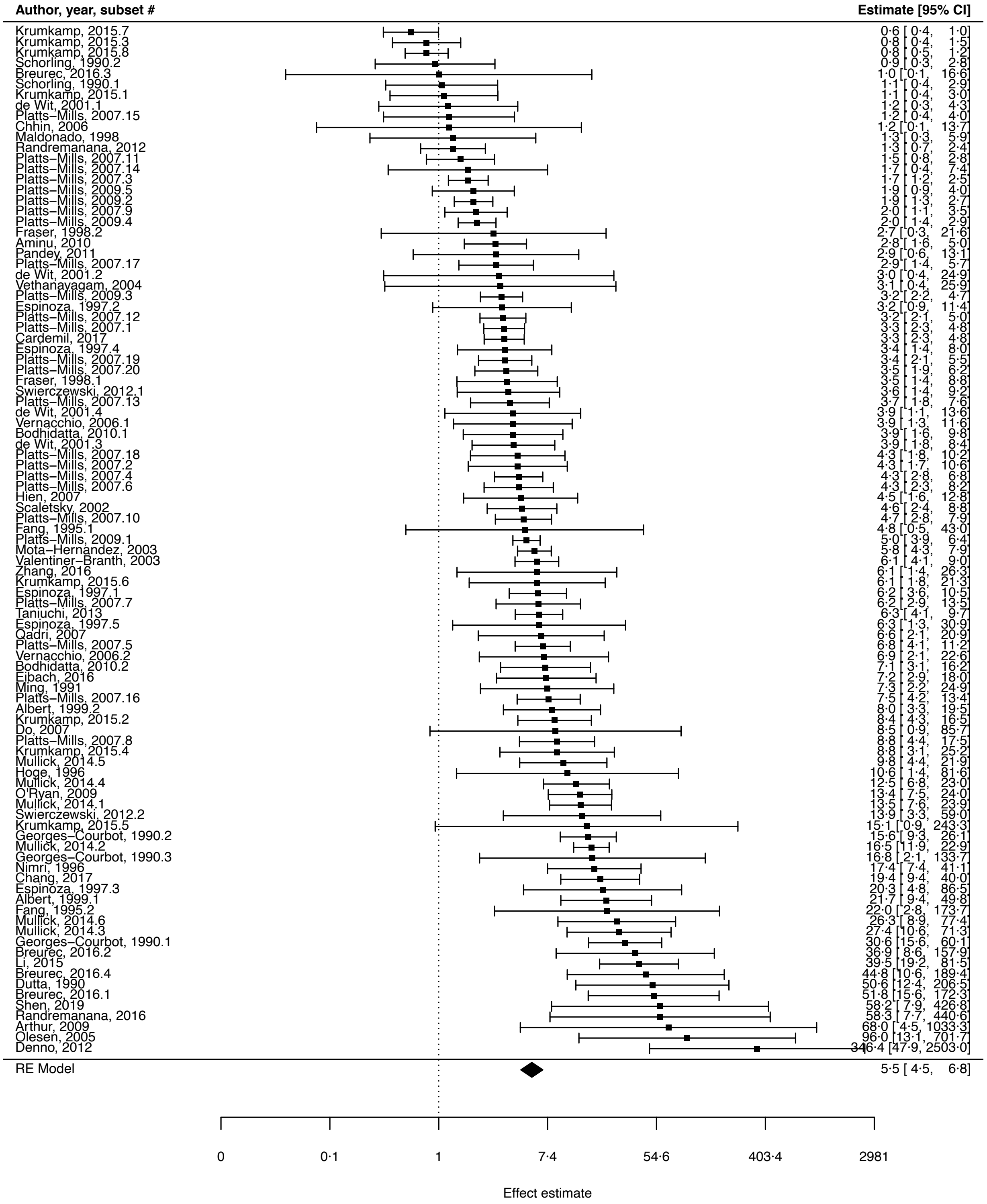

Figure S6. Rotavirus (post-vaccine introduction) forest plot

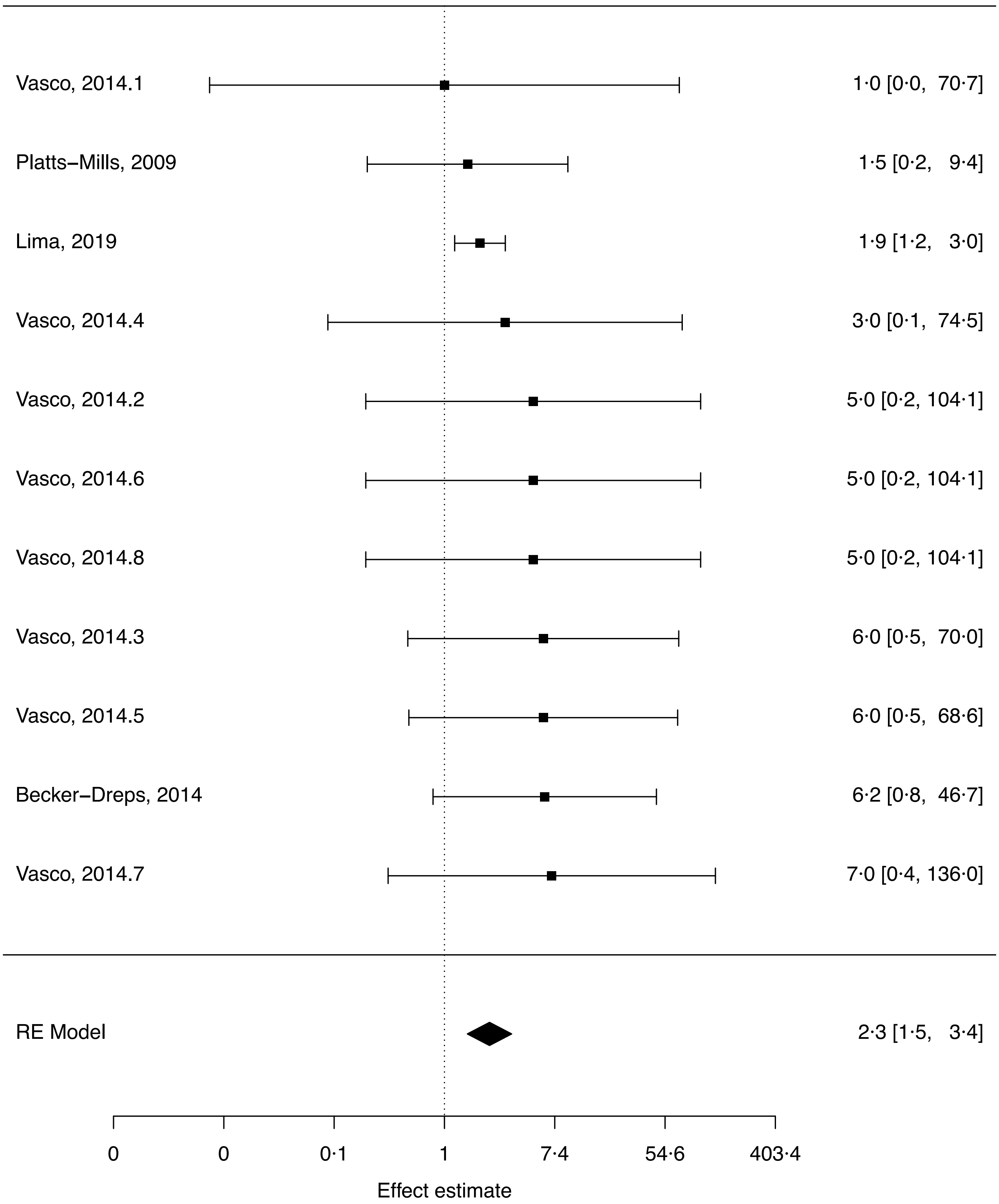

Figure S7. Sapovirus forest plot

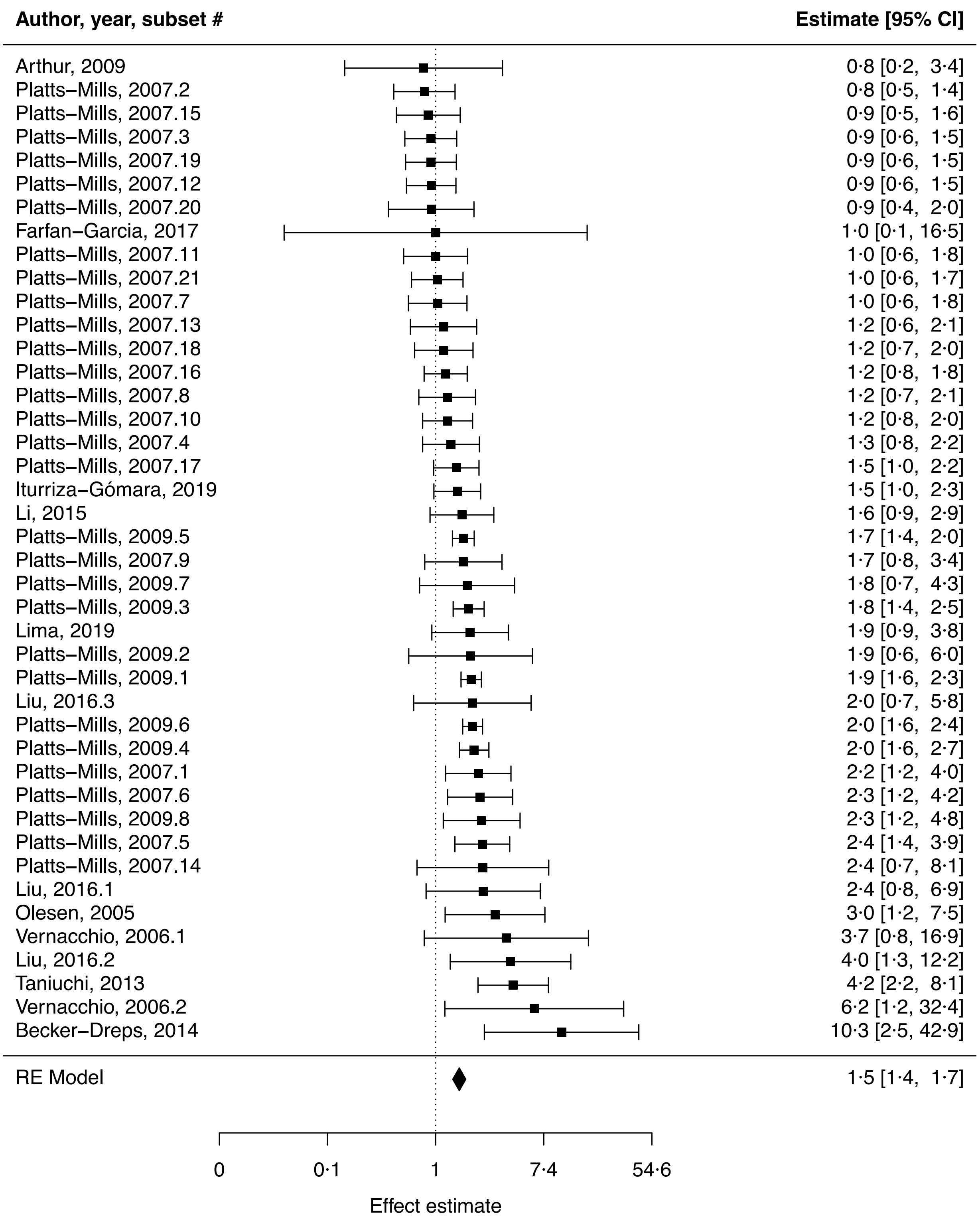

Figure S8. ***Aeromonas*** **forest plot**

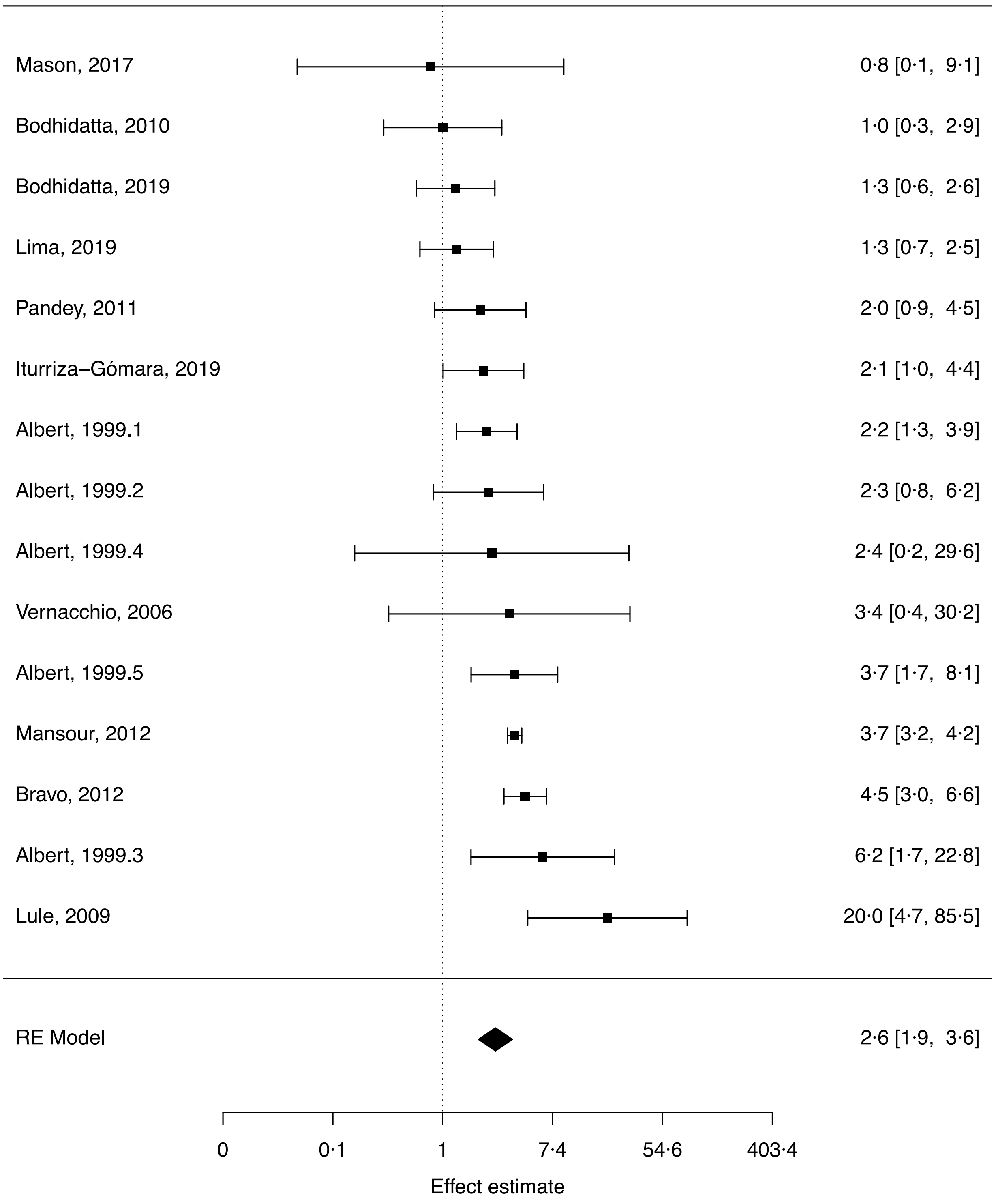

Figure S9. *Campylobacter* forest plot

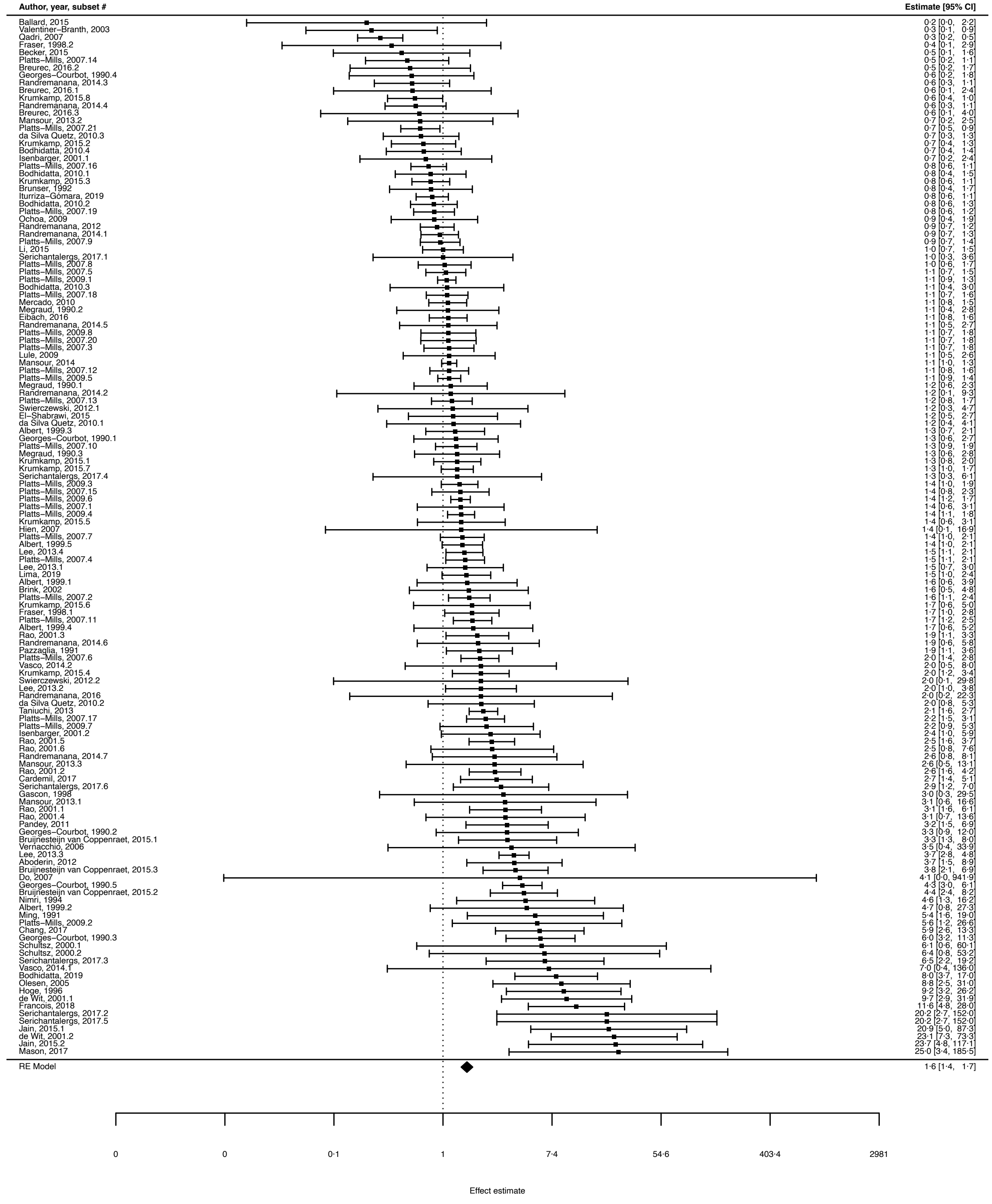

Figure S10. ***V cholerae*** **forest plot**

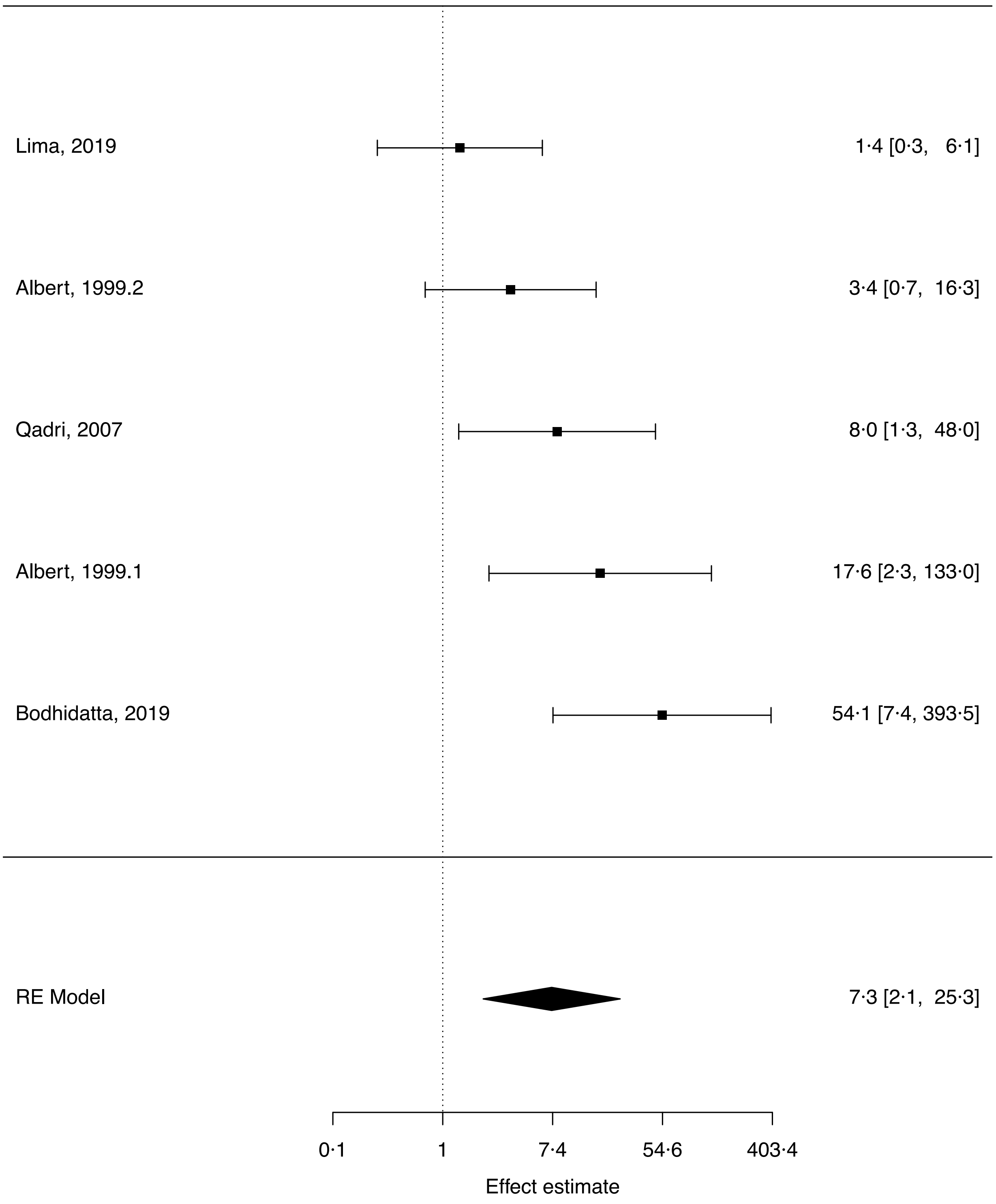

Figure S11. Atypical EPEC forest plot

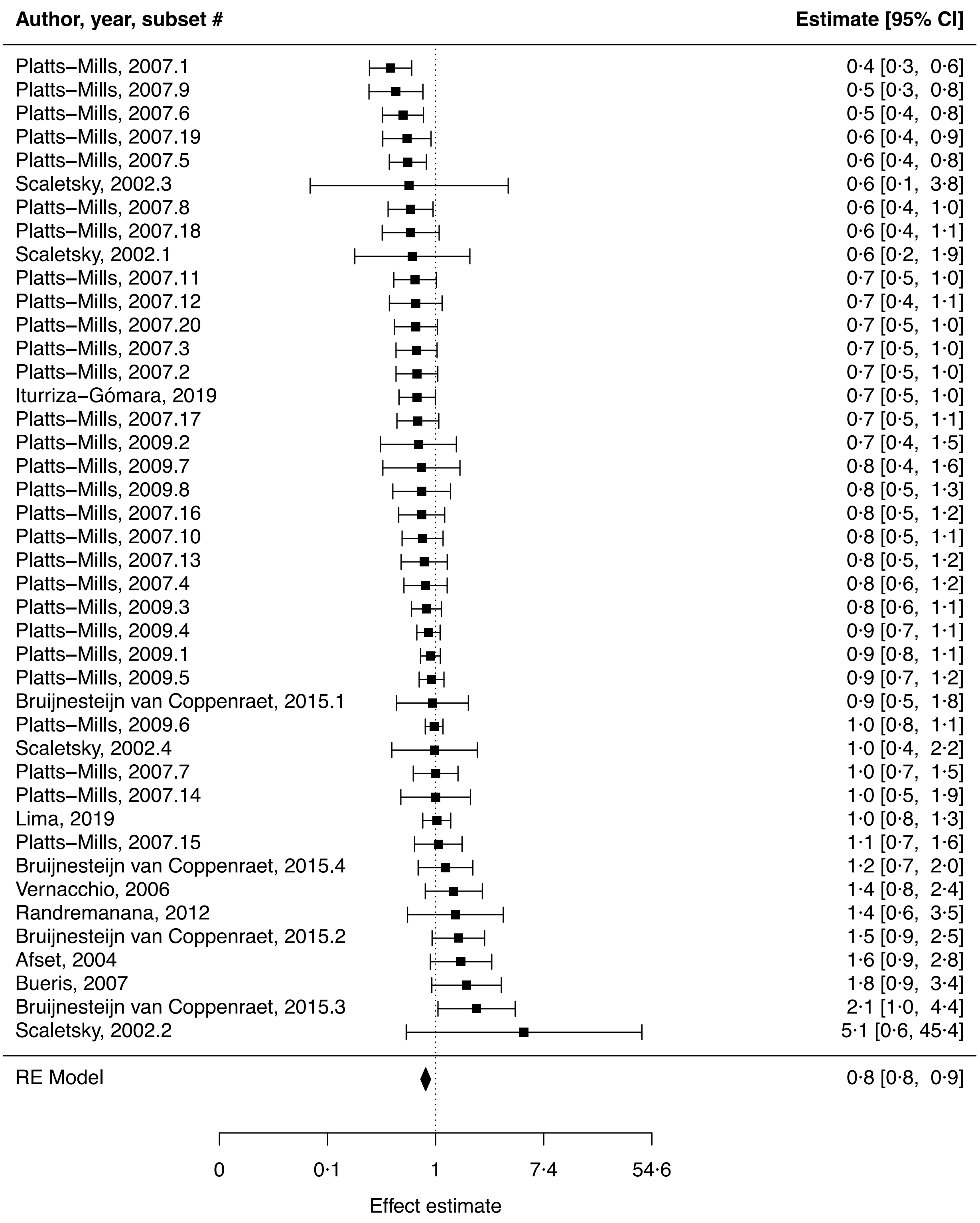

Figure S12. Typical EPEC forest plot

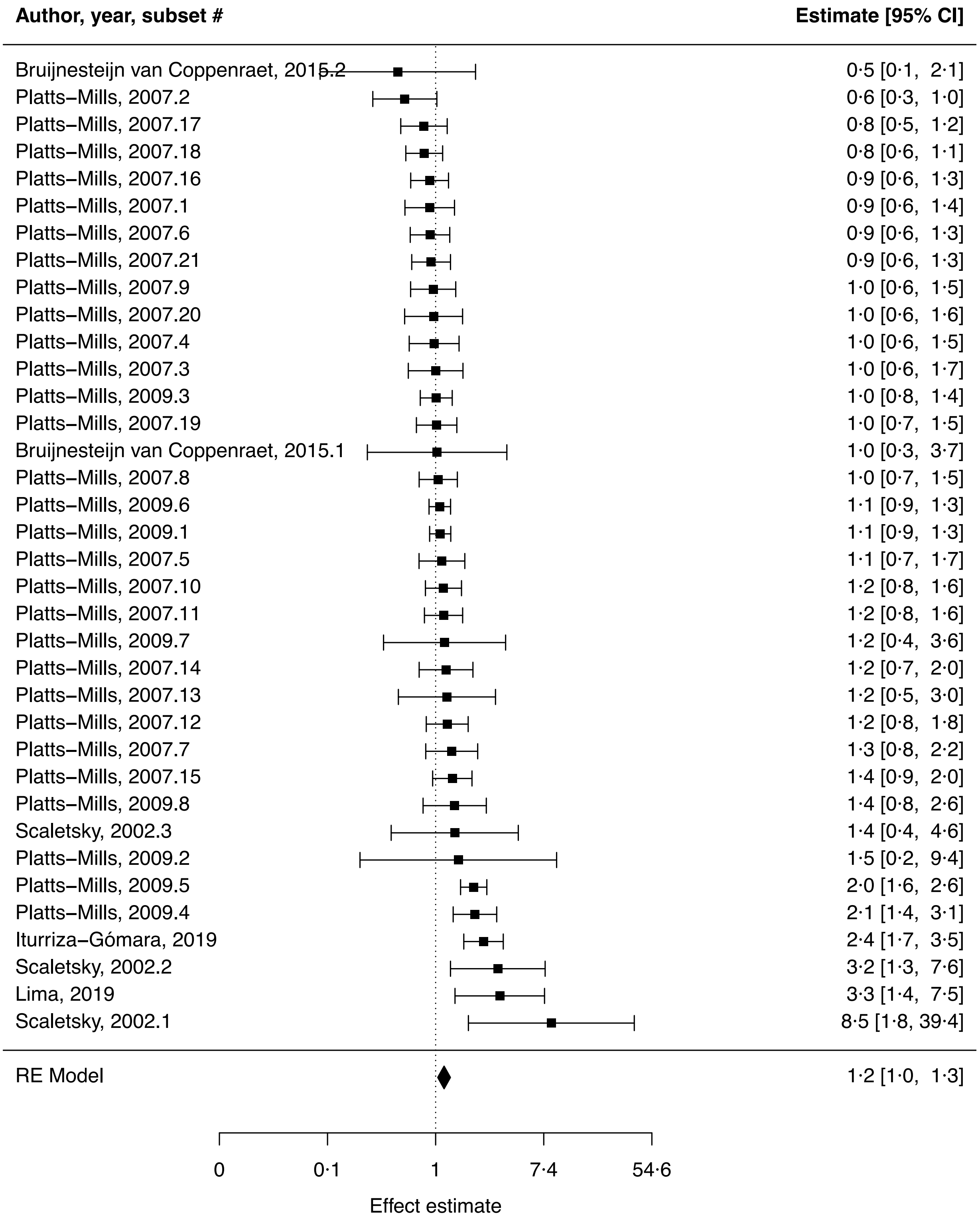

Figure S13. EPEC- unknown subgroup forest plot

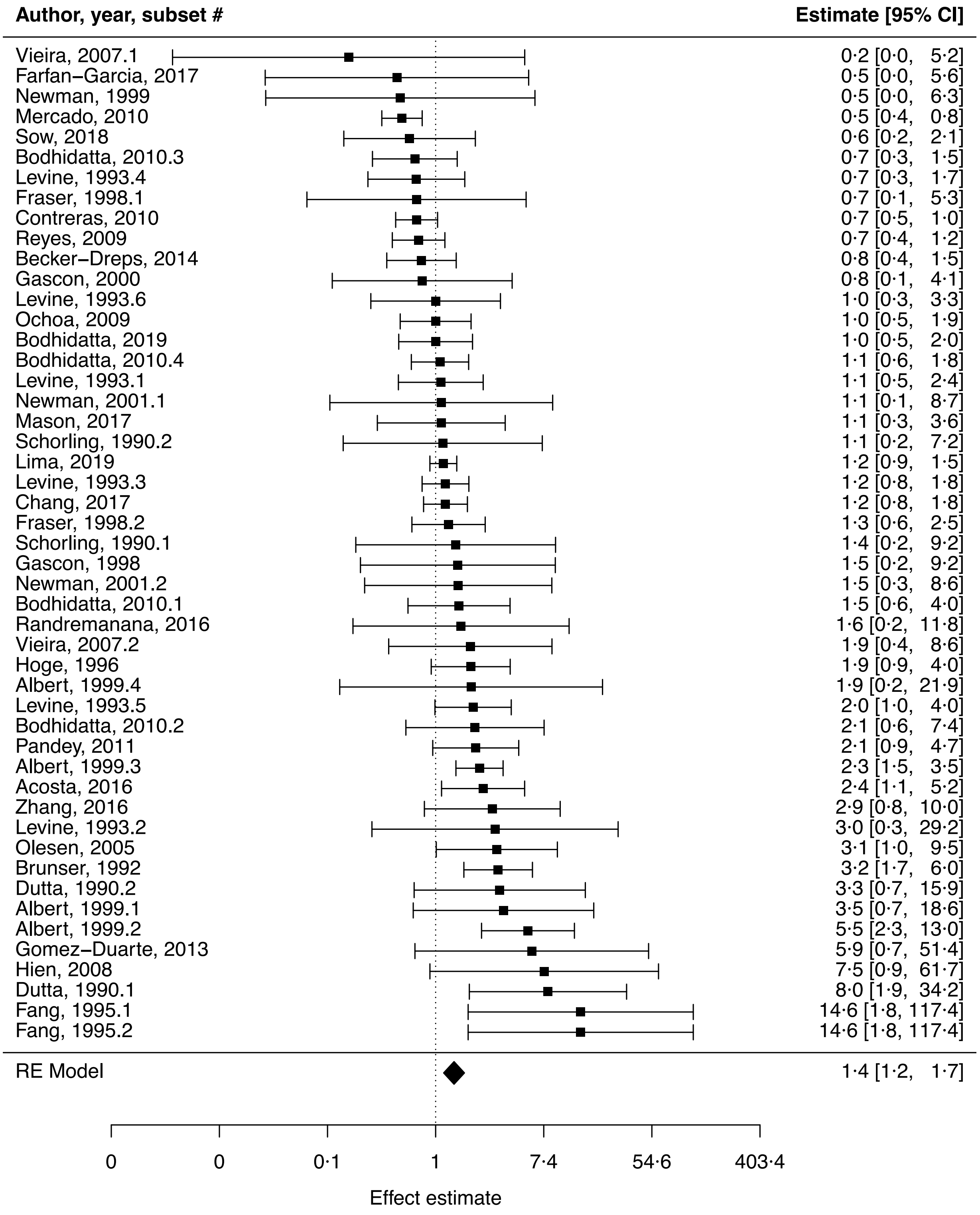

Figure S14. ST ETEC forest plot

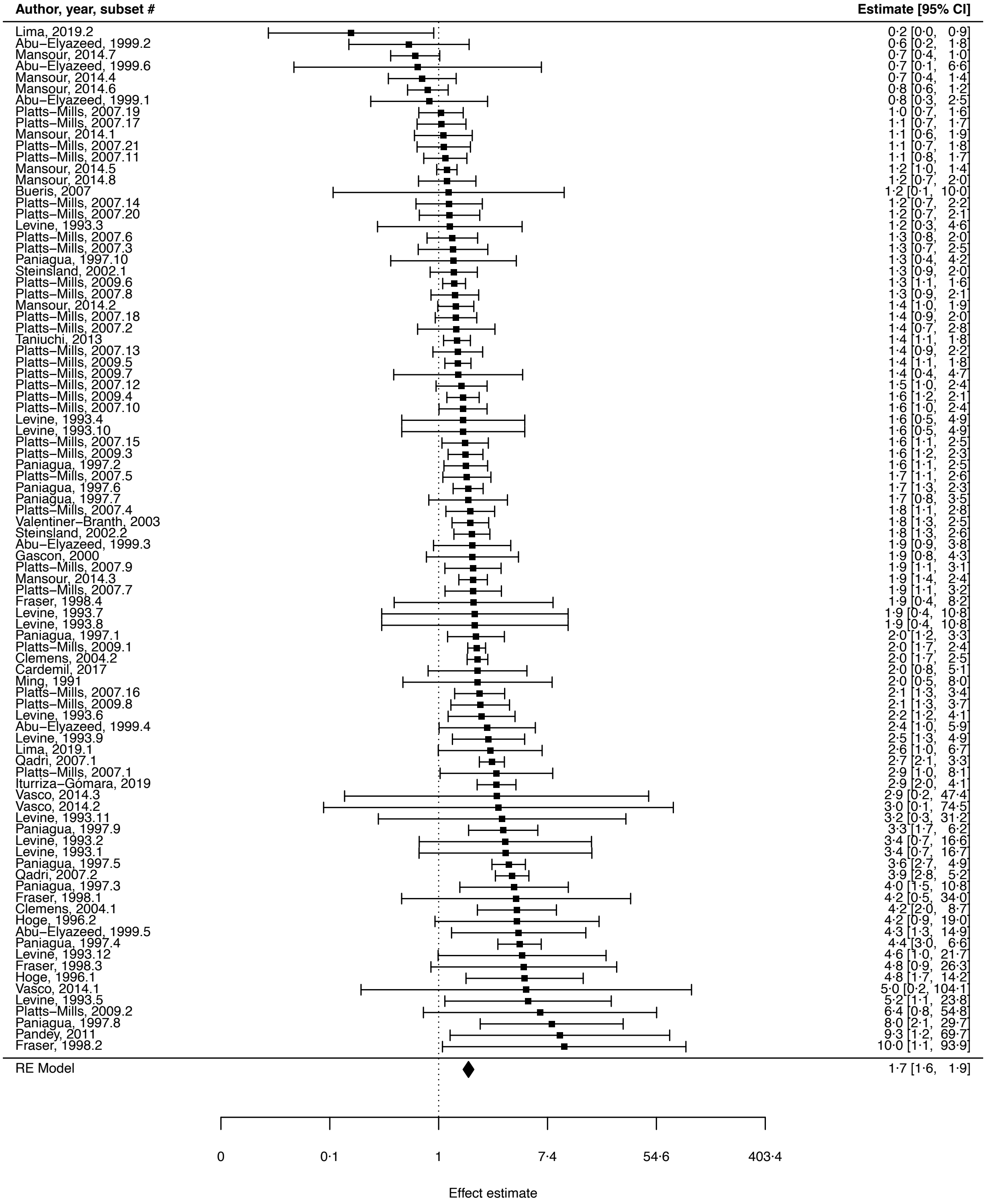

Figure S15. LT ETEC forest plot

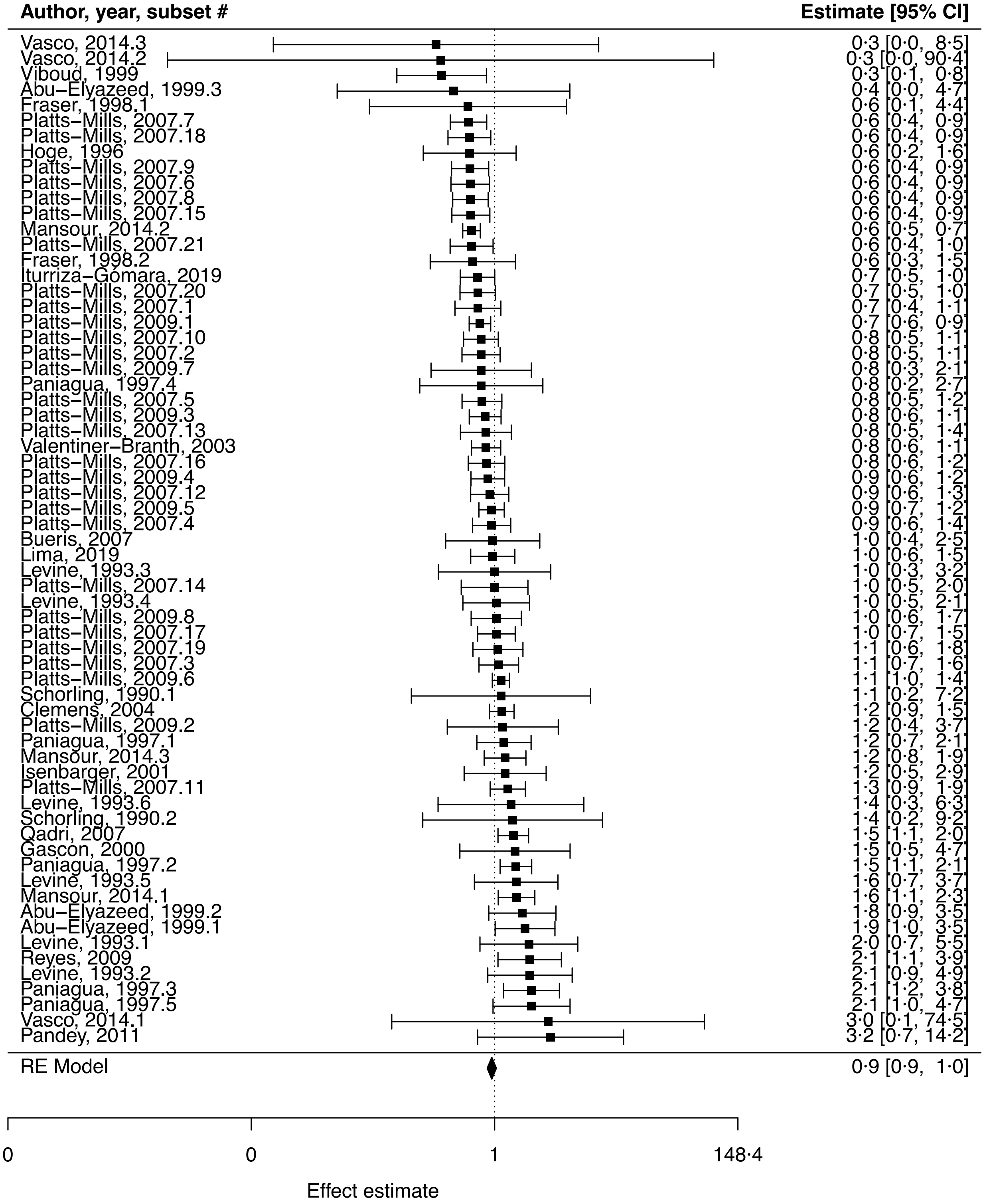

Figure S16. ETEC- unknown subgroup forest plot

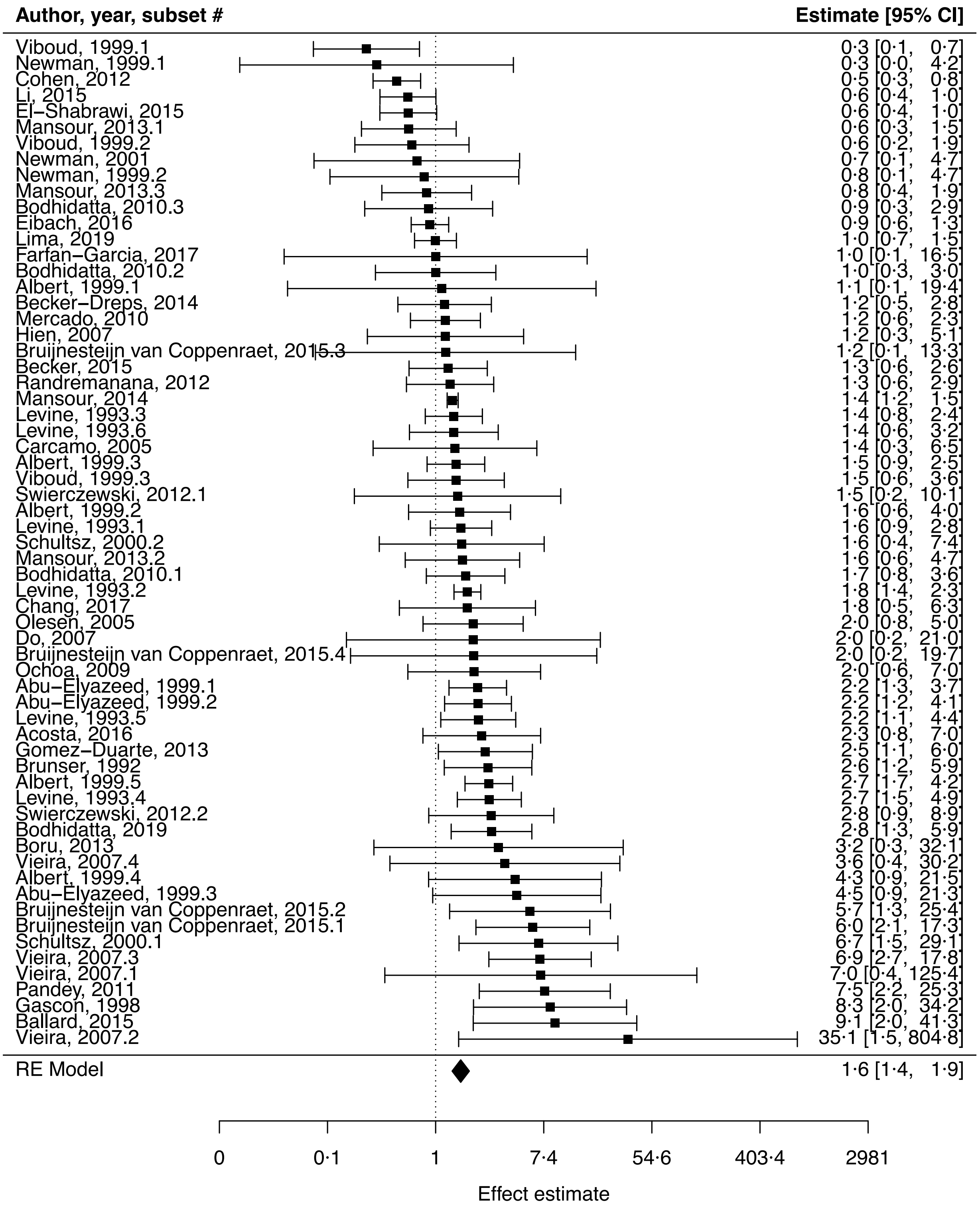

Figure S17. *Salmonella* forest plot

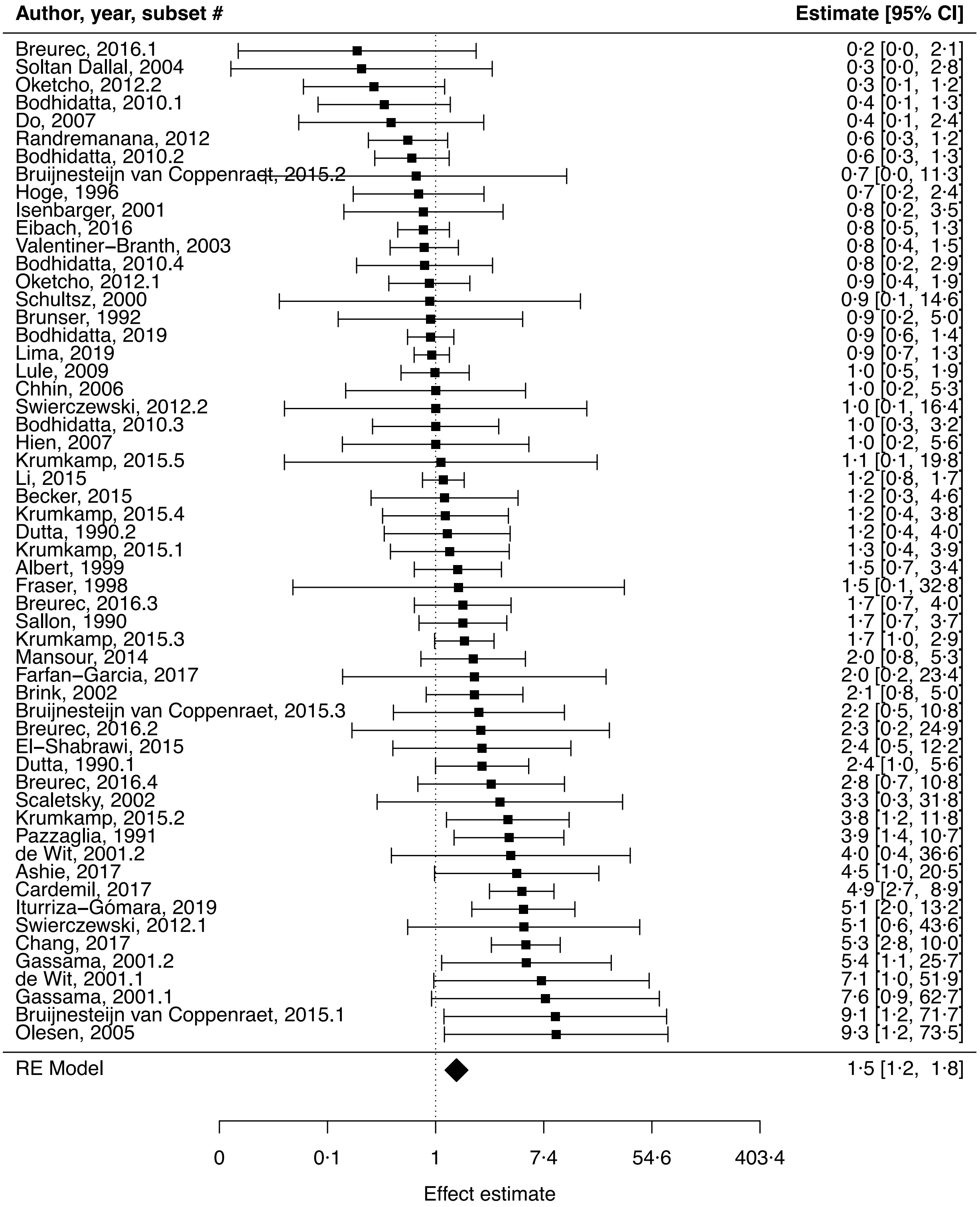

Figure S18. *Shigella* forest plot

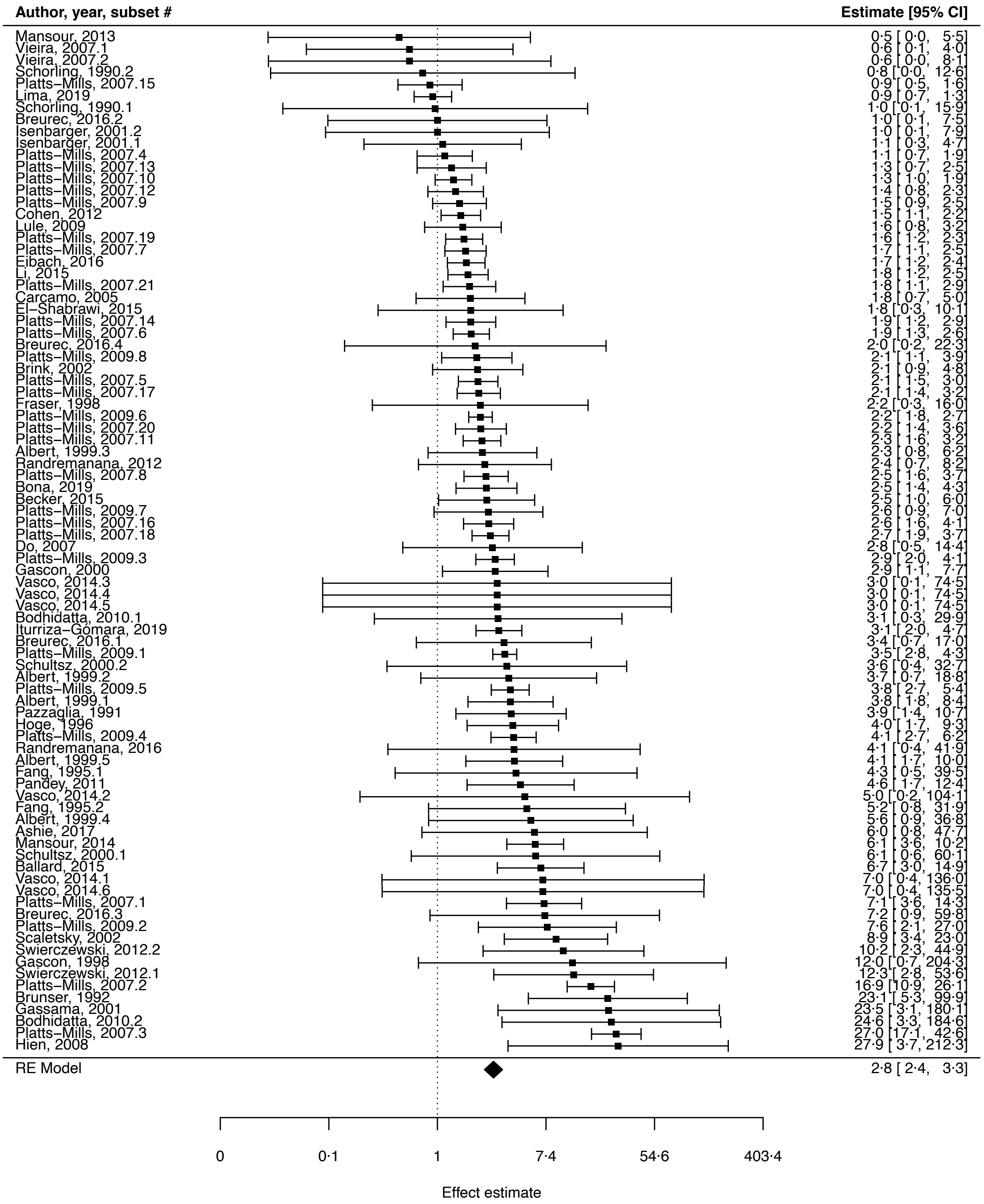

Figure S19. *Cryptosporidium* forest plot

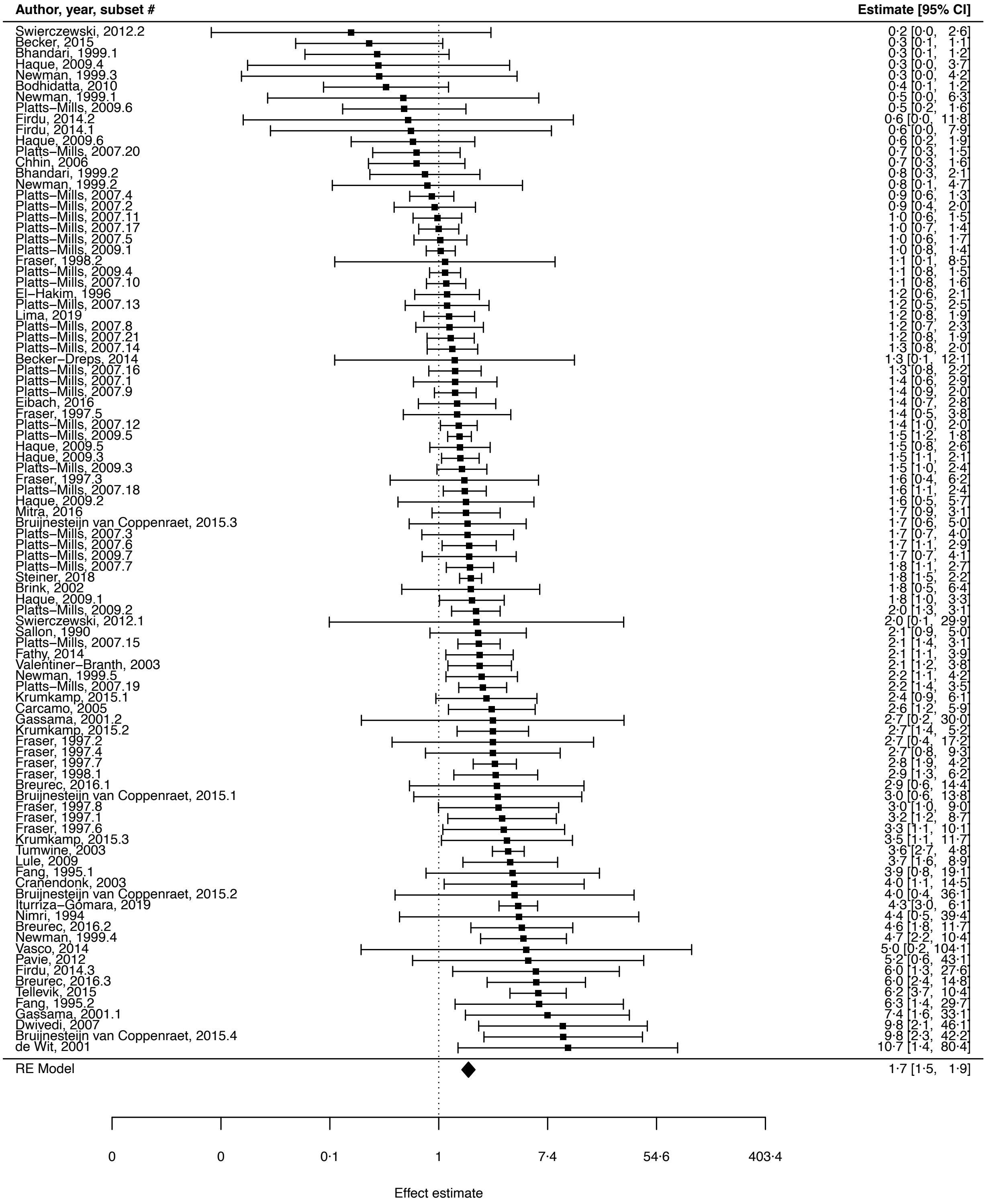

Figure S20. ***E. histolytica*** **forest plot**

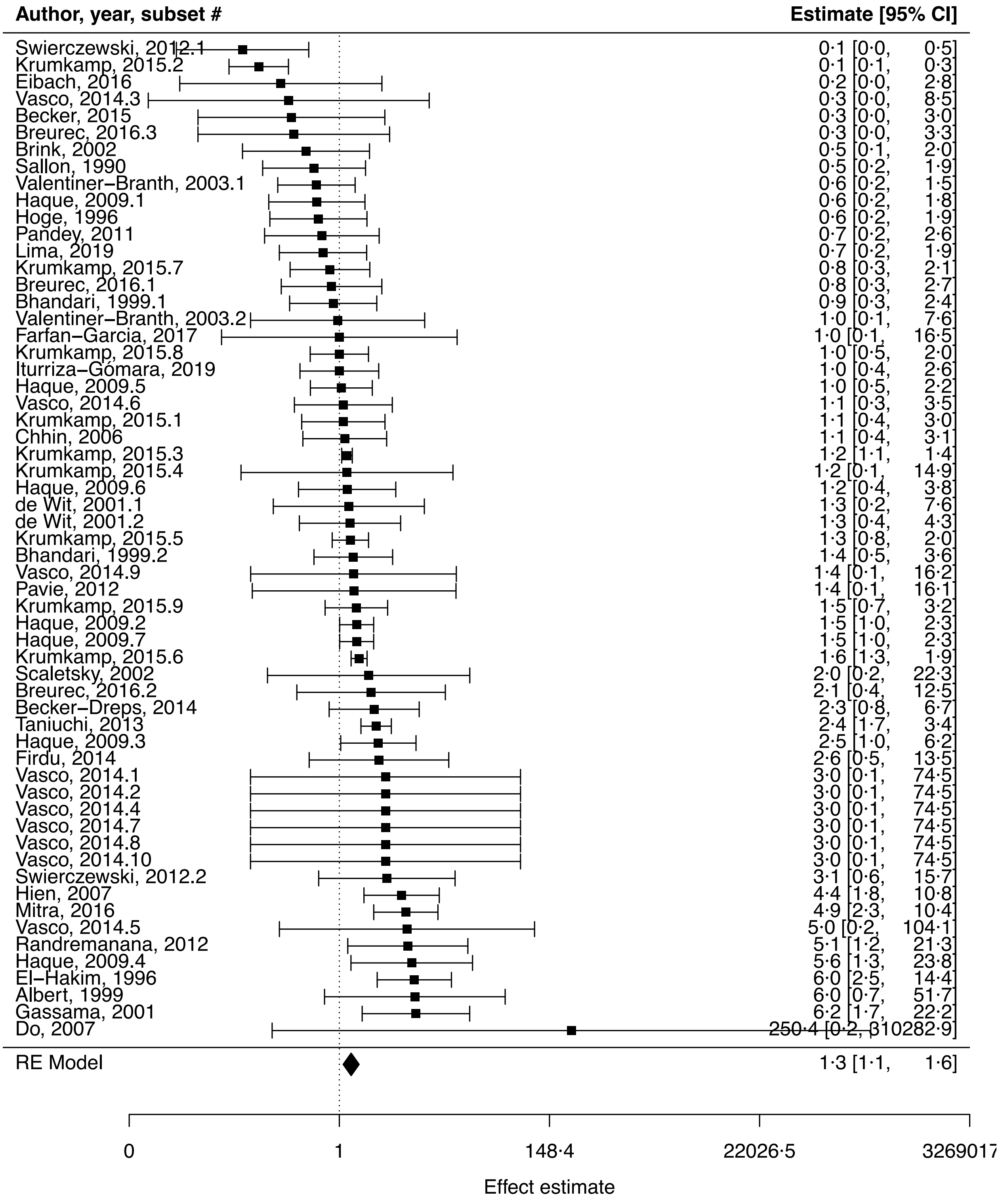

Figure S21. *Giardia lamblia* forest plot

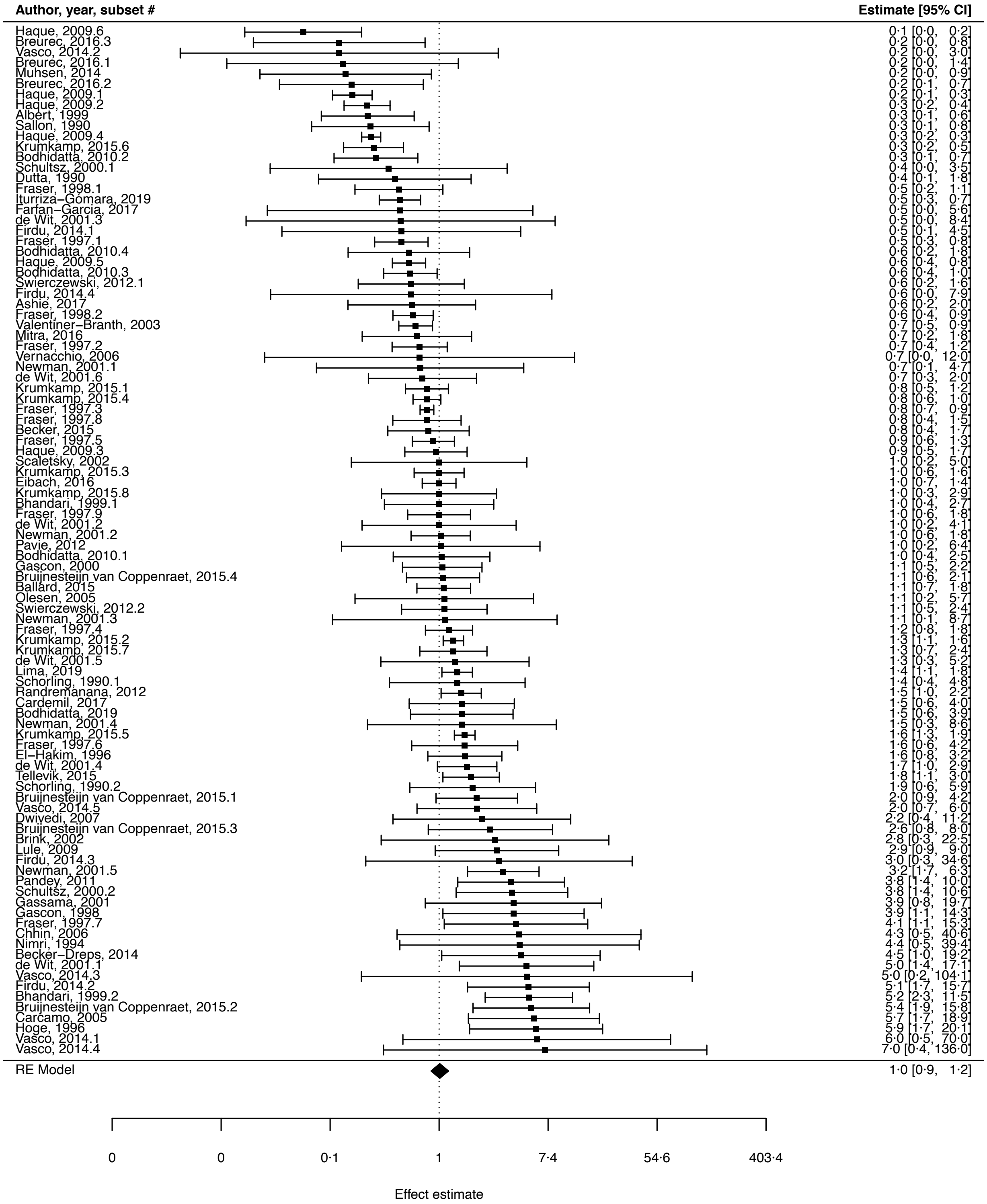

Table S3. Effect estimate type and study characteristics among 1,240 observations from 130 studies

Study Characteristic	All effect measures n (%)	Adjusted OR n (%)	Unadjusted OR n (%)	Adjusted RR n (%)	Unadjusted RR n (%)	Unadjusted OR- calculated n (%)
Total observations [a]	1240 (100·0)	144 (11·6)	174 (14·0)	26 (2·1)	28 (2·3)	868 (70·0)
Study Design						
Prospective cohort	229 (18·5)	28 (19·4)	34 (19·5)	8 (30·8)	26 (92·9)	133 (15·3)
Retrospective cohort	6 (0·5)	0 (0·0)	0 (0·0)	0 (0·0)	0 (0·0)	6 (0·7)
Randomized control trial	5 (0·4)	0 (0·0)	0 (0·0)	0 (0·0)	0 (0·0)	5 (0·6)
Cross-sectional	1 (0·1)	0 (0·0)	0 (0·0)	0 (0·0)	0 (0·0)	1 (0·1)
Case control	835 (67·3)	89 (61·8)	131 (75·3)	18 (69·2)	1 (3·6)	596 (68·7)
Nested case control	164 (13·2)	27 (18·8)	9 (5·2)	0 (0·0)	1 (3·6)	127 (14·6)
Characteristics by which study participants were matched [b]						
Age	357 (28·8)	43 (37·7)	68 (39·1)	0 (0·0)	2 (7·1)	244 (28·1)
Sex	144 (11·6)	27 (18·8)	40 (23·0)	0 (0·0)	1 (3·6)	76 (8·8)
Location	217 (17·5)	50 (34·7)	36 (20·7)	18 (69·2)	0 (0·0)	113 (13·0)
Other	72 (5·8)	14 (9·7)	4 (2·3)	0 (0·0)	1 (3·6)	53 (6·1)
Unmatched/not indicated	445 (35·9)	80 (55·6)	66 (37·9)	8 (30·8)	26 (92·9)	265 (30·5)
Study validity score [c]						
1	19 (1·5)	0 (0·0)	1 (0·6)	0 (0·0)	0 (0·0)	18 (2·1)
2	42 (3·4)	15 (10·4)	6 (3·4)	0 (0·0)	1 (3·6)	20 (2·3)
3	373 (30·1)	27 (18·8)	142 (81·6)	26 (100·0)	9 (32·1)	169 (19·5)
4	399 (32·2)	84 (58·3)	25 (14·4)	0 (0·0)	18 (64·3)	272 (31·3)
5	63 (5·1)	18 (12·5)	0 (0·0)	0 (0·0)	0 (0·0)	45 (5·2)
Not applicable [d]	344 (27·7)	0 (0·0)	0 (0·0)	0 (0·0)	0 (0·0)	344 (39·6)

[a] Row percent
[b] Not exclusive
[c] Validity score calculated by awarding 1 point for each of the following criteria (maximum score of 5): 1) case/outcome-positive definition provided, 2) control/outcome-negative definition provided, 3) diarrhea presentation defined, 4) lab certification or quality framework described, and 5) diarrhea definition provided.
[d] Additional data included in analysis and not assessed during the systematic review (Global Enteric Multicenter Study and the Malnutrition and Enteric Disease study)
OR, odds ratio; RR, relative risk.

Table S4. Comparison of heterogeneity (I^2) by pathogen and effect estimate type using 1,240 observations from 130 studies

Enteropathogen	OR only	OR & RR
Adenovirus 40/41	84·0	83·8
Astrovirus	28·4	27·6
Norovirus	75·9	76·0
Rotavirus	82·0	85·5
Pre-vaccine	83·2	86·6
Post-vaccine	0·0	0·0
Sapovirus	53·3	52·9
Aeromonas	58·0	63·4
Campylobacter	78·1	78·5
V. cholerae	60·5	60·5
aEPEC	45·9	47·1
tEPEC	60·6	60·6
EPEC- unknown subgroup	60·2	56·2
ST ETEC	62·1	66·3
LT ETEC	56·6	57·7
ETEC- unknown subgroup	64·0	62·1
Salmonella	55·0	55·0
Shigella	79·0	79·0
Cryptosporidium	65·3	65·3
E. histolytica	60·5	58·9
Giardia lamblia	82·3	84·0

E. histolytica, *Entamoeba histolytica*; aEPEC, atypical enteropathogenic *E. coli;* tEPEC, typical enteropathogenic *E. coli;* ST ETEC, heat-stable enterotoxigenic *E. coli (*enterotoxigenic *E. coli* that harbor the ST gene regardless of LT gene status)*;* LT ETEC, heat-labile enterotoxigenic *E. coli (*enterotoxigenic *E. coli* that only harbor the LT gene and not the ST gene)*;* OR, odds ratio; RR, relative risk; *V. cholerae*, *Vibrio cholerae*

Table S5. Random effects meta-analysis results from unadjusted model stratified by pathogen detection method [a]

Enteropathogen	PCR<br>OR (95% CI)	Non-PCR<br>OR (95% CI)
Adenovirus 40/41	1·6 (1·3, 2·0)	2·1 (1·4, 3·2)
Astrovirus	1·5 (1·3, 1·7)	1·9 (1·3, 2·6)
Norovirus	1·5 (1·3, 1·7)	2·5 (1·8, 3·4)
Rotavirus	5·3 (4·2, 6·7)	5·6 (4·1, 7·7)
Pre-vaccine [b]	5·3 (4·2, 6·8)	5·8 (4·1, 8·3)
Post-vaccine [b]	1·9 (1·2, 2·9)	5·1 (2·0, 12·7)
Sapovirus	1·5 (1·4, 1·7)	
Aeromonas	1·6 (1·0, 2·6)	2·9 (2·1, 4·0)
Campylobacter	1·3 (1·2, 1·5)	1·9 (1·5, 2·3)
V. cholerae	1·4 (0·3, 6·1)	11·4 (3·5, 36·6)
aEPEC	0·8 (0·8, 0·9)	
tEPEC	1·2 (1·0, 1·3)	
EPEC- unknown subgroup	1·3 (1·0, 1·7)	1·6 (1·2, 2·1)
ST ETEC	1·7 (1·5, 1·8)	1·8 (1·5, 2·2)
LT ETEC	0·9 (0·8, 1·0)	1·2 (0·9, 1·6)
ETEC- unknown subgroup	1·9 (1·5, 2·6)	1·4 (1·2, 1·7)
Salmonella	1·9 (1·3, 3·0)	1·3 (1·0, 1·6)
Shigella	2·4 (2·0, 3·0)	3·6 (2·8, 4·5)
Cryptosporidium	1·6 (1·4, 1·8)	2·1 (1·7, 2·6)
E. histolytica	1·1 (0·9, 1·5)	1·5 (1·1, 2·0)
Giardia lamblia	1·1 (0·9, 1·4)	1·0 (0·8, 1·2)

[a] Unadjusted model stratified by whether or not PCR was used for pathogen detection in a given study.
[b] Pre-vaccine defined as a study that began prior to nationwide introduction of the rotavirus vaccine, post-vaccine defined as a study that began 1 or more years after nationwide introduction of the rotavirus vaccine. Rotavirus vaccine introduction status determined according to the WHO.
Blanks represent pathogens for which data were available in the analytic dataset but with too few effect estimates available to produce a summary OR.
CI, confidence interval; *E. histolytica*, *Entamoeba histolytica;* aEPEC, atypical enteropathogenic *E. coli;* tEPEC, typical enteropathogenic *E. coli;* ST ETEC, heat-stable enterotoxigenic *E. coli (*enterotoxigenic *E. coli* that harbor the ST gene regardless of LT gene status)*;* LT ETEC, heat-labile enterotoxigenic *E. coli (*enterotoxigenic *E. coli* that only harbor the LT gene and not the ST gene); OR, odds ratio; PCR, polymerase chain reaction; *V. cholerae*, *Vibrio cholerae.*

Table S6. Random effects meta-analysis results from unadjusted model, unadjusted model stratified by pathogen detection method, and adjusted model stratified by age group and child mortality setting

	Unadjusted model [a]	Stratified and adjusted models [b]						
		0-1 years of age		2-4 years of age		0-4 years of age		≥5 years of age
		Very low/low child mortality	High child mortality	Very low/low child mortality	High child mortality	Very low/low child mortality	High child mortality	All child mortality levels
**Enteropathogen**	**OR (95% CI)**	**OR (95% CI)**	**OR (95% CI)**	**OR (95% CI)**	**OR (95% CI)**	**OR (95% CI)**	**OR (95% CI)**	**OR (95% CI)**
Adenovirus 40/41	1·7 (1·4, 2·1)	8·6 (2·4, 30·3)	1·3 (0·6, 2·8)		1·2 (0·9, 1·7)	6·0 (2·2, 16·4)	1·3 (0·7, 2·3)	0·4 (0·0, 6·9)
Astrovirus	1·5 (1·4, 1·7)	1·5 (1·1, 1·9)	2·1 (1·1, 4·4)		1·6 (1·1, 2·3)	1·7 (0·7, 4·4)	1·7 (1·1, 2·8)	2·0 (0·4, 11·1)
Norovirus	1·5 (1·4, 1·7)	2·1 (0·9, 4·7)	2·9 (1·2, 6·8)		1·1 (0·8, 1·5)	3·5 (1·1, 11·3)	2·9 (1·6, 5·5)	3·2 (1·3, 7·6)
Rotavirus	5·4 (4·5, 6·6)	4·4 (2·6, 7·7)	12·5 (7·8, 20·1)		2·7 (1·6, 4·4)	6·8 (3·7, 12·5)	9·9 (6·2, 15·9)	2·9 (1·3, 6·5)
Pre-vaccine [c]	5·5 (4·5, 6·8)	4·9 (2·8, 8·8)	12·5 (7·8, 20·1)		2·7 (1·6, 4·4)	9·0 (5·2, 15·6)	9·4 (6·1, 14·7)	2·9 (1·3, 6·5)
Post-vaccine [c]	2·3 (1·5, 3·4)	1·5 (0·2, 9·4)				6·2 (0·8, 46·7)		
Sapovirus	1·5 (1·4, 1·7)	2·0 (1·7, 2·4)	1·4 (1·2, 1·6)		1·5 (1·1, 2·0)	2·0 (1·7, 2·4)	1·4 (1·2, 1·6)	
Aeromonas	2·6 (1·9, 3·6)		3·7 (3·0, 4·5)		5·1 (1·6, 16·1)		3·7 (3·2, 4·2)	1·5 (0·9, 2·6)
Campylobacter	1·6 (1·4, 1·7)	1·1 (0·8, 1·6)	1·9 (1·5, 2·5)		2·3 (0·9, 6·2)	1·2 (0·8, 1·6)	1·7 (1·4, 2·1)	5·1 (2·6, 10·0)
V. cholerae	7·3 (2·1, 25·3)		6·8 (1·4, 32·9)			1·4 (0·3, 6·1)	6·8 (1·4, 32·9)	54·1 (7·4, 393·5)
aEPEC	0·8 (0·8, 0·9)	1·0 (0·8, 1·1)	0·7 (0·7, 0·8)		0·7 (0·6, 0·9)	1·1 (0·9, 1·3)	0·8 (0·7, 0·8)	1·4 (1·0, 1·9)
tEPEC	1·2 (1·0, 1·3)	2·0 (1·0, 4·2)	1·1 (1·0, 1·3)		1·0 (0·8, 1·2)	2·0 (1·1, 3·6)	1·1 (1·0, 1·3)	0·5 (0·1, 2·1)
EPEC- unknown subgroup	1·4 (1·2, 1·7)	1·0 (0·6, 2·0)	3·3 (1·4, 7·7)	1·3 (0·4, 3·7)	1·9 (0·2, 21·9)	1·7 (0·4, 7·6)	2·6 (1·6, 4·1)	2·0 (1·2, 3·4)
ST ETEC	1·7 (1·6, 1·9)	2·0 (0·4, 9·3)	1·3 (0·9, 2·0)	1·9 (0·9, 4·1)	1·4 (1·2, 1·7)	2·0 (0·5, 9·2)	1·5 (1·1, 2·0)	5·2 (2·3, 11·5)
LT ETEC	0·9 (0·9, 1·0)	1·0 (0·5, 2·2)	1·5 (1·2, 2·1)	1·3 (0·7, 2·6)	0·8 (0·6, 0·9)	0·4 (0·2, 1·0)	1·0 (0·8, 1·3)	1·2 (0·2, 6·3)
ETEC- unknown subgroup	1·6 (1·4, 1·9)	0·4 (0·1, 1·6)	1·4 (0·8, 2·4)	0·3 (0·1, 0·7)	3·1 (0·8, 12·7)	0·5 (0·2, 1·1)	1·3 (0·9, 1·9)	1·4 (0·1, 30·8)
Salmonella	1·5 (1·2, 1·8)	1·1 (0·5, 2·3)	1·1 (0·2, 6·4)			1·0 (0·6, 1·5)	1·4 (0·8, 2·4)	0·9 (0·6, 1·3)
Shigella	2·8 (2·4, 3·3)	6·7 (1·8, 25·1)	4·0 (1·7, 9·2)		5·6 (0·4, 77·9)	7·3 (3·5, 15·3)	3·3 (2·1, 5·2)	2·8 (1·5, 5·5)
Cryptosporidium	1·7 (1·5, 1·9)	2·5 (1·2, 5·2)	2·1 (0·9, 5·1)		1·1 (0·9, 1·3)	1·5 (0·8, 2·8)	0·9 (0·5, 1·7)	3·4 (1·2, 9·6)
E. histolytica	1·3 (1·1, 1·6)		1·8 (0·4, 7·3)			0·6 (0·2, 1·9)	1·7 (1·0, 2·9)	1·2 (0·6, 2·3)
Giardia lamblia	1·0 (0·9, 1·2)	0·4 (0·2, 0·6)	0·9 (0·1, 6·5)	0·2 (0·0, 0·9)		0·6 (0·4, 0·9)	0·9 (0·5, 1·7)	1·2 (0·6, 2·3)

[a] Unadjusted model results represent the crude summary estimate for a given pathogen.
[b] Adjusted models stratified by age and child mortality setting and adjusted for pathogen detection method (reference: conventional detection methods) and study design (reference: case-control study design).
[c] Pre-vaccine defined as a study that began prior to nationwide introduction of the rotavirus vaccine, post-vaccine defined as a study that began 1 or more years after nationwide introduction of the rotavirus vaccine. Rotavirus vaccine introduction status determined according to the WHO.
Blanks represent pathogens for which data were available in the analytic dataset but with too few effect estimates available to produce a summary OR.
CI, confidence interval; *E. histolytica*, *Entamoeba histolytica;* aEPEC, atypical enteropathogenic *E. coli;* tEPEC, typical enteropathogenic *E. coli;* ST ETEC, heat-stable enterotoxigenic *E. coli (*enterotoxigenic *E. coli* that harbor the ST gene regardless of LT gene status)*;* LT ETEC, heat-labile enterotoxigenic *E. coli (*enterotoxigenic *E. coli* that only harbor the LT gene and not the ST gene); OR, odds ratio; PCR, polymerase chain reaction; *V. cholerae*, *Vibrio cholerae.*

Table S7. Strain-specific random effects meta-analysis results from unadjusted mode and adjusted model stratified by age group and child mortality setting

	Unadjusted model [a]	Stratified and adjusted models [b]		
		0-4 years of age		≥5 years of age
		Very low/low child mortality	High child mortality	All child mortality levels
Enteropathogen	OR (95% CI)	OR (95% CI)	OR (95% CI)	OR (95% CI)
Norovirus				
Norovirus GI	1·3 (0·6, 2·8)	1·4 (0·3, 6·6)	0·8 (0·5, 1·2)	1·4 (0·4, 4·4)
Norovirus GII	1·4 (1·2, 1·6)	3·8 (1·8, 8·0)	1·1 (1·0, 1·2)	3·4 (1·3, 8·8)
Rotavirus				
Rotavirus A	6·1 (1·4, 26·3)			
Aeromonas				
Aeromonas hydrophila	3·7 (3·2, 4·2)		3·7 (3·2, 4·2)	
Campylobacter				
C. coli	1·5 (1·1, 2·0)	0·7 (0·2, 2·4)	2·5 (0·8, 7·6)	1·1 (0·4, 3·0)
C. jejuni	2·0 (1·5, 2·7)	5·4 (1·6, 19·0)	1·7 (1·2, 2·6)	4·1 (0·0, 1068·2)
C. lari	1·5 (0·7, 3·0)			
V. cholerae				
V. cholerae O1	17·6 (2·3, 133·0)		17·6 (2·3, 133·0)	
V. cholerae O139	3·4 (0·7, 16·3)		3·4 (0·7, 16·3)	
EPEC				
aEPEC	0·8 (0·8, 0·9)	1·1 (0·9, 1·3)	0·8 (0·7, 0·8)	1·4 (1·0, 1·9)
tEPEC	1·2 (1·0, 1·3)	2·0 (1·1, 3·6)	1·1 (1·0, 1·3)	0·5 (0·1, 2·1)
EPEC- unknown subgroup	1·4 (1·2, 1·7)	1·7 (0·4, 7·6)	2·6 (1·6, 4·1)	2·0 (1·2, 3·4)
ETEC				
ST ETEC	1·7 (1·6, 1·9)	2·0 (0·5, 9·2)	1·5 (1·1, 2·0)	5·2 (2·3, 11·5)
LT ETEC	0·9 (0·9, 1·0)	0·4 (0·2, 1·0)	1·0 (0·8, 1·3)	1·2 (0·2, 6·3)
ETEC- unknown subgroup	1·6 (1·4, 1·9)	0·5 (0·2, 1·1)	1·3 (0·9, 1·9)	1·4 (0·1, 30·8)
Salmonella				
Non-typhoidal salmonella	1·1 (0·5, 2·5)	0·8 (0·2, 3·5)	0·3 (0·1, 1·2)	0·6 (0·2, 1·7)
S. paratyphi	0·8 (0·4, 1·6)	0·3 (0·0, 2·8)	0·9 (0·4, 1·9)	
Shigella				
S. dysenteriae [Group A]	1·0 (0·1, 7·9)	1·0 (0·1, 7·9)		
S. boydii [Group C]	1·1 (0·3, 4·7)	1·1 (0·3, 4·7)		
S. sonnei [Group D]	1·5 (1·1, 2·2)			1·5 (1·1, 2·2)
Cryptosporidium				
C. parvum	3·0 (2·3, 4·0)	2·5 (1·2, 5·2)		5·8 (2·1, 15·5)
E. histolytica				
E. coli	0·3 (0·1, 1·1)		0·6 (0·2, 1·5)	
E. dispar	1·3 (1·2, 1·5)			
E. histolytica	1·5 (1·2, 2·0)	0·5 (0·2, 1·9)	1·6 (0·7, 3·3)	1·1 (0·5, 2·3)
Giardia lamblia				
Giardia lamblia, intestinalis, or duodenalis	0·9 (0·7, 1·1)	0·6 (0·4, 0·8)	0·7 (0·3, 1·4)	0·5 (0·2, 1·2)

[a] Unadjusted model results represent the crude summary estimate for a given strain-specific pathogen.
[b] Adjusted models stratified by age and child mortality setting and adjusted for pathogen detection method (reference: conventional detection methods) and study design (reference: case-control study design).
Blanks represent pathogens for which data were available in the analytic dataset but with too few effect estimates available to produce a summary OR.
CI, confidence interval; *E. histolytica*, *Entamoeba histolytica;* aEPEC, atypical enteropathogenic *E. coli;* tEPEC, typical enteropathogenic *E. coli;* ST ETEC, heat-stable enterotoxigenic *E. coli (*enterotoxigenic *E. coli* that harbor the ST gene regardless of LT gene status)*;* LT ETEC, heat-labile enterotoxigenic *E. coli (*enterotoxigenic *E. coli* that only harbor the LT gene and not the ST gene); OR, odds ratio; *V. cholerae*, *Vibrio cholera.*

Table S8. Random effects meta-analysis results from unadjusted model and adjusted model stratified by age group and child mortality setting, excluding influential outliers [a]

	Unadjusted model [b]	Stratified and adjusted models [c]						
		0-1 years of age		2-4 years of age		0-4 years of age		≥5 years of age
		Very low/low child mortality	High child mortality	Very low/low child mortality	High child mortality	Very low/low child mortality	High child mortality	All child mortality levels
**Enteropathogen**	**OR (95% CI)**	**OR (95% CI)**	**OR (95% CI)**	**OR (95% CI)**	**OR (95% CI)**	**OR (95% CI)**	**OR (95% CI)**	**OR (95% CI)**
Adenovirus 40/41	1·5 (1·3, 1·7)	8·6 (2·4, 30·3)	1·3 (0·8, 2·3)		1·2 (0·9, 1·7)	6·0 (2·2, 16·4)	1·3 (0·9, 1·9)	0·4 (0·0, 6·9)
Astrovirus	1·5 (1·3, 1·7)	1·5 (1·1, 1·9)	2·1 (1·1, 4·4)		1·6 (1·1, 2·3)	1·8 (0·7, 4·9)	1·7 (1·0, 2·8)	2·0 (0·4, 11·1)
Norovirus	1·5 (1·3, 1·7)	2·1 (0·9, 4·7)	2·9 (1·2, 6·8)		1·1 (0·8, 1·5)	3·0 (1·1, 8·1)	2·9 (1·6, 5·5)	3·2 (1·3, 7·6)
Rotavirus	5·4 (4·5, 6·6)	4·4 (2·6, 7·7)	12·5 (7·8, 20·1)		2·7 (1·6, 4·4)	6·8 (3·7, 12·5)	9·9 (6·2, 15·9)	2·9 (1·3, 6·5)
Pre-vaccine [d]	5·5 (4·5, 6·8)	4·9 (2·8, 8·8)	12·5 (7·8, 20·1)		2·7 (1·6, 4·4)	9·0 (5·2, 15·6)	9·4 (6·1, 14·7)	2·9 (1·3, 6·5)
Post-vaccine [d]	2·3 (1·5, 3·4)	1·5 (0·2, 9·4)				6·2 (0·8, 46·7)		
Sapovirus	1·5 (1·4, 1·7)	2·0 (1·7, 2·4)	1·4 (1·2, 1·6)		1·5 (1·1, 2·0)	2·0 (1·7, 2·4)	1·4 (1·2, 1·6)	
Aeromonas	2·6 (1·9, 3·6)		3·7 (3·0, 4·5)		5·1 (1·6, 16·1)		3·7 (3·2, 4·2)	1·5 (0·9, 2·6)
Campylobacter	1·4 (1·3, 1·5)	1·1 (0·8, 1·6)	1·7 (1·3, 2·2)		2·3 (0·9, 6·2)	1·1 (0·8, 1·5)	1·5 (1·3, 1·9)	4·3 (1·9, 9·7)
V. cholerae	6·8 (2·5, 18·8)		6·8 (1·4, 32·9)				6·8 (1·4, 32·9)	
aEPEC	0·8 (0·8, 0·9)	1·0 (0·8, 1·1)	0·7 (0·7, 0·8)		0·7 (0·6, 0·9)	1·1 (0·9, 1·3)	0·8 (0·7, 0·8)	1·4 (1·0, 1·9)
tEPEC	1·1 (1·0, 1·2)	2·0 (1·0, 4·2)	1·1 (1·0, 1·2)		1·0 (0·8, 1·2)	2·0 (1·1, 3·6)	1·1 (1·0, 1·1)	0·5 (0·1, 2·1)
EPEC- unknown subgroup	1·3 (1·1, 1·6)	1·0 (0·6, 2·0)	2·3 (1·5, 3·5)	1·3 (0·4, 3·7)	1·9 (0·2, 21·9)	1·7 (0·4, 7·6)	2·1 (1·4, 3·2)	2·0 (1·2, 3·4)
ST ETEC	1·6 (1·5, 1·8)	2·0 (0·5, 8·3)	1·3 (0·9, 1·8)	1·9 (0·9, 4·1)	1·4 (1·2, 1·7)	2·0 (0·5, 8·4)	1·5 (1·1, 1·9)	5·2 (2·3, 11·5)
LT ETEC	0·9 (0·9, 1·0)	1·0 (0·5, 2·2)	1·5 (1·2, 2·1)	1·3 (0·7, 2·6)	0·8 (0·6, 0·9)	0·4 (0·2, 1·0)	1·0 (0·8, 1·3)	1·2 (0·2, 6·3)
ETEC- unknown subgroup	1·6 (1·4, 1·9)	0·4 (0·1, 1·6)	1·4 (0·8, 2·4)	0·3 (0·1, 0·7)	3·1 (0·8, 12·7)	0·5 (0·2, 1·1)	1·3 (0·9, 1·9)	1·4 (0·1, 30·8)
Salmonella	1·3 (1·1, 1·6)	1·1 (0·5, 2·3)	1·1 (0·2, 6·1)			1·0 (0·6, 1·5)	1·4 (0·9, 2·3)	0·9 (0·6, 1·3)
Shigella	2·5 (2·2, 2·8)	6·7 (1·8, 25·1)	4·0 (2·0, 8·0)		5·6 (0·8, 37·7)	7·3 (3·5, 15·3)	3·5 (2·5, 5·0)	2·8 (1·5, 5·5)
Cryptosporidium	1·6 (1·5, 1·8)	2·5 (1·2, 5·2)	2·1 (1·0, 4·3)		1·1 (0·9, 1·3)	1·5 (0·8, 2·8)	1·0 (0·6, 1·6)	3·4 (1·2, 9·6)
E. histolytica	1·4 (1·2, 1·7)		1·8 (0·4, 7·3)			0·6 (0·2, 1·9)	1·7 (1·0, 2·9)	1·2 (0·6, 2·3)
Giardia lamblia	1·0 (0·9, 1·2)	0·4 (0·2, 0·6)	0·9 (0·1, 6·5)	0·2 (0·0, 0·9)		0·6 (0·4, 0·9)	0·9 (0·5, 1·7)	1·2 (0·6, 2·3)

[a] Influential outliers identified for each pathogen based on Studentized Residual Plots and Cook's distances.
[b] Unadjusted model results represent the crude summary estimate for a given pathogen.
[c] Adjusted models stratified by age and child mortality setting and adjusted for pathogen detection method (reference: conventional detection methods) and study design (reference: case-control study design).
[d] Pre-vaccine defined as a study that began prior to nationwide introduction of the rotavirus vaccine, post-vaccine defined as a study that began 1 or more years after nationwide introduction of the rotavirus vaccine. Rotavirus vaccine introduction status determined according to the WHO.
Blanks represent pathogens for which data were available in the analytic dataset but with too few effect estimates available to produce a summary OR.
CI, confidence interval; *E. histolytica*, *Entamoeba histolytica;* aEPEC, atypical enteropathogenic *E. coli;* tEPEC, typical enteropathogenic *E. coli;* ST ETEC, heat-stable enterotoxigenic *E. coli (*enterotoxigenic *E. coli* that harbor the ST gene regardless of LT gene status)*;* LT ETEC, heat-labile enterotoxigenic *E. coli (*enterotoxigenic *E. coli* that only harbor the LT gene and not the ST gene); OR, odds ratio*; V. cholerae*, *Vibrio cholerae.*

Table S9. Random effects meta-analysis results from unadjusted model and adjusted model stratified by age group and child mortality setting, excluding relative risk effect estimates

	Unadjusted model [a]	Stratified and adjusted models [b]						
		0-1 years of age		2-4 years of age		0-4 years of age		≥5 years of age
		Very low/low child mortality	High child mortality	Very low/low child mortality	High child mortality	Very low/low child mortality	High child mortality	All child mortality levels
**Enteropathogen**	**OR (95% CI)**	**OR (95% CI)**	**OR (95% CI)**	**OR (95% CI)**	**OR (95% CI)**	**OR (95% CI)**	**OR (95% CI)**	**OR (95% CI)**
Adenovirus 40/41	1·7 (1·4, 2·0)	8·6 (2·4, 30·3)	1·3 (0·6, 2·8)		1·2 (0·9, 1·7)	8·6 (3·2, 23·2)	1·3 (0·7, 2·3)	0·4 (0·0, 6·9)
Astrovirus	1·5 (1·4, 1·7)	1·5 (1·1, 1·9)	2·1 (1·1, 4·4)		1·6 (1·1, 2·3)	3·1 (0·8, 12·0)	1·7 (1·1, 2·8)	2·0 (0·4, 11·1)
Norovirus	1·6 (1·4, 1·8)	2·1 (0·9, 4·7)	2·9 (1·2, 6·8)		1·1 (0·8, 1·5)	4·1 (1·3, 12·9)	2·9 (1·6, 5·5)	3·2 (1·3, 7·6)
Rotavirus	5·9 (4·9, 7·1)	4·4 (2·6, 7·7)	12·5 (7·8, 20·1)		2·7 (1·6, 4·4)	7·1 (3·7, 13·7)	9·9 (6·2, 15·9)	2·9 (1·3, 6·5)
Pre-vaccine [c]	6·1 (5·0, 7·3)	4·9 (2·8, 8·8)	12·5 (7·8, 20·1)		2·7 (1·6, 4·4)	7·8 (4·5, 13·6)	9·4 (6·1, 14·7)	2·9 (1·3, 6·5)
Post-vaccine [c]	2·3 (1·5, 3·4)	1·5 (0·2, 9·4)				6·2 (0·8, 46·7)		
Sapovirus	1·5 (1·4, 1·7)	2·0 (1·7, 2·4)	1·4 (1·2, 1·6)		1·5 (1·1, 2·0)	2·0 (1·7, 2·4)	1·4 (1·2, 1·6)	
Aeromonas	2·5 (1·7, 3·6)		2·6 (1·7, 4·2)		5·1 (1·6, 16·1)		2·8 (1·9, 4·1)	1·5 (0·9, 2·6)
Campylobacter	1·6 (1·4, 1·7)	1·1 (0·8, 1·6)	1·9 (1·5, 2·5)		2·3 (0·9, 6·2)	1·2 (0·8, 1·6)	1·7 (1·4, 2·1)	5·1 (2·6, 10·0)
V. cholerae	7·3 (2·1, 25·3)		6·8 (1·4, 32·9)			1·4 (0·3, 6·1)	6·8 (1·4, 32·9)	54·1 (7·4, 393·5)
aEPEC	0·8 (0·8, 0·9)	1·0 (0·8, 1·1)	0·7 (0·7, 0·8)		0·7 (0·6, 0·9)	1·1 (0·9, 1·3)	0·8 (0·7, 0·8)	1·4 (1·0, 1·9)
tEPEC	1·2 (1·0, 1·3)	2·0 (1·0, 4·2)	1·1 (1·0, 1·3)		1·0 (0·8, 1·2)	2·0 (1·1, 3·6)	1·1 (1·0, 1·3)	0·5 (0·1, 2·1)
EPEC- unknown subgroup	1·5 (1·2, 1·8)	1·0 (0·4, 2·3)	3·3 (1·4, 7·7)		1·9 (0·2, 21·9)	1·5 (0·3, 7·6)	2·6 (1·6, 4·1)	2·0 (1·2, 3·4)
ST ETEC	1·8 (1·6, 2·0)	2·0 (0·4, 9·3)	2·2 (1·5, 3·0)	1·9 (0·9, 4·1)	1·4 (1·2, 1·7)	2·0 (0·4, 9·4)	1·8 (1·4, 2·2)	5·2 (2·3, 11·5)
LT ETEC	0·9 (0·8, 1·0)	1·0 (0·5, 2·2)	1·6 (1·2, 2·2)	1·3 (0·7, 2·6)	0·8 (0·6, 0·9)	0·4 (0·2, 1·0)	1·0 (0·7, 1·3)	1·2 (0·2, 6·3)
ETEC- unknown subgroup	1·6 (1·3, 2·0)	0·5 (0·1, 2·7)	1·4 (0·8, 2·4)	0·3 (0·1, 0·7)	3·1 (0·8, 12·7)	0·6 (0·2, 1·9)	1·3 (0·8, 2·1)	1·4 (0·1, 30·8)
Salmonella	1·5 (1·2, 1·8)	1·1 (0·5, 2·3)	1·1 (0·2, 6·4)			1·0 (0·6, 1·5)	1·4 (0·8, 2·4)	0·9 (0·6, 1·3)
Shigella	2·8 (2·4, 3·3)	6·7 (1·8, 25·1)	4·0 (1·7, 9·2)		5·6 (0·4, 77·9)	7·3 (3·5, 15·3)	3·3 (2·1, 5·2)	2·8 (1·5, 5·5)
Cryptosporidium	1·7 (1·5, 1·9)	2·5 (1·2, 5·2)	2·1 (0·9, 5·1)		1·1 (0·9, 1·3)	1·5 (0·8, 2·8)	0·9 (0·5, 1·7)	3·4 (1·2, 9·6)
E. histolytica	1·3 (1·1, 1·7)		1·8 (0·4, 7·3)			0·6 (0·2, 1·9)	1·7 (1·0, 2·9)	1·2 (0·6, 2·3)
Giardia lamblia	1·0 (0·9, 1·2)	0·4 (0·2, 0·6)	0·9 (0·1, 6·5)	0·2 (0·0, 0·9)		0·6 (0·4, 0·9)	0·9 (0·5, 1·7)	1·2 (0·6, 2·3)

[a] Unadjusted model results represent the crude summary estimate for a given pathogen.

[b] Adjusted models stratified by age and child mortality setting and adjusted for pathogen detection method (reference: conventional detection methods) and study design (reference: case-control study design).

[c] Pre-vaccine defined as a study that began prior to nationwide introduction of the rotavirus vaccine, post-vaccine defined as a study that began 1 or more years after nationwide introduction of the rotavirus vaccine. Rotavirus vaccine introduction status determined according to the WHO.

Blanks represent pathogens for which data were available in the analytic dataset but with too few effect estimates available to produce a summary OR.

CI, confidence interval; *E. histolytica*, *Entamoeba histolytica;* aEPEC, atypical enteropathogenic *E. coli;* tEPEC, typical enteropathogenic *E. coli;* ST ETEC, heat-stable enterotoxigenic *E. coli (*enterotoxigenic *E. coli* that harbor the ST gene regardless of LT gene status)*;* LT ETEC, heat-labile enterotoxigenic *E. coli (*enterotoxigenic *E. coli* that only harbor the LT gene and not the ST gene); OR, odds ratio; *V. cholerae*, *Vibrio cholerae.*

Table S10. Random effects meta-analysis results from unadjusted model and adjusted model stratified by age group and child mortality setting, excluding coinfections indicated by authors

	Unadjusted model [a]	Stratified and adjusted models [b]						
		0-1 years of age		2-4 years of age		0-4 years of age		≥5 years of age
		Very low/low child mortality	High child mortality	Very low/low child mortality	High child mortality	Very low/low child mortality	High child mortality	All child mortality levels
Enteropathogen	OR (95% CI)	OR (95% CI)	OR (95% CI)	OR (95% CI)	OR (95% CI)	OR (95% CI)	OR (95% CI)	OR (95% CI)
Adenovirus 40/41	2·6 (1·7, 4·0)	8·6 (2·4, 30·3)	1·3 (0·8, 2·2)			6·3 (3·3, 11·8)	1·3 (0·5, 3·0)	0·4 (0·0, 6·9)
Astrovirus	1·8 (1·4, 2·3)	1·5 (1·1, 1·9)	2·0 (0·6, 6·5)			1·9 (0·7, 5·3)	1·6 (0·8, 3·4)	2·0 (0·4, 11·1)
Norovirus	2·2 (1·8, 2·6)	2·5 (1·4, 4·7)	2·9 (1·3, 6·7)			4·4 (1·7, 11·1)	2·9 (1·5, 5·8)	3·2 (1·3, 7·6)
Rotavirus	7·9 (6·2, 9·9)	4·4 (2·5, 7·8)	13·4 (8·2, 21·9)		26·3 (8·9, 77·4)	7·3 (3·9, 13·9)	12·5 (6·9, 22·7)	2·9 (1·3, 6·5)
Pre-vaccine [c]	8·2 (6·5, 10·3)	4·9 (2·8, 8·8)	13·4 (8·2, 21·9)		26·3 (8·9, 77·4)	9·0 (5·2, 15·6)	11·2 (6·4, 19·7)	2·9 (1·3, 6·5)
Post-vaccine [c]	2·1 (1·3, 3·2)							
Sapovirus	2·4 (1·7, 3·3)	2·6 (1·4, 4·9)	4·2 (2·2, 8·1)			2·1 (1·5, 2·9)		
Aeromonas	2·6 (1·9, 3·6)		3·7 (3·0, 4·5)		5·1 (1·6, 16·1)		3·7 (3·2, 4·2)	1·5 (0·9, 2·6)
Campylobacter	1·8 (1·6, 2·1)	1·1 (0·8, 1·5)	1·8 (1·2, 2·7)		2·3 (0·9, 5·8)	1·2 (0·8, 1·7)	1·7 (1·3, 2·2)	5·1 (2·6, 10·0)
V. cholerae	7·3 (2·1, 25·3)		6·8 (1·4, 32·9)			1·4 (0·3, 6·1)	6·8 (1·4, 32·9)	54·1 (7·4, 393·5)
aEPEC	1·2 (1·0, 1·4)	0·9 (0·5, 1·7)				1·2 (0·9, 1·7)	0·9 (0·5, 1·7)	1·4 (1·0, 1·9)
tEPEC	2·2 (1·4, 3·6)	3·1 (1·3, 7·2)				2·6 (1·5, 4·7)	2·4 (1·7, 3·5)	0·5 (0·1, 2·1)
EPEC- unknown subgroup	1·4 (1·2, 1·7)	1·0 (0·6, 2·0)	3·3 (1·4, 7·7)	1·3 (0·4, 3·7)	1·9 (0·2, 21·9)	1·7 (0·4, 7·6)	2·6 (1·6, 4·1)	2·0 (1·2, 3·4)
ST ETEC	1·9 (1·7, 2·3)	2·0 (0·5, 9·3)	1·4 (0·7, 2·6)	1·9 (0·9, 4·1)		2·0 (0·4, 9·3)	1·6 (1·1, 2·4)	5·2 (2·3, 11·5)
LT ETEC	1·2 (1·0, 1·4)	1·3 (0·7, 2·5)	1·8 (1·2, 2·9)	1·3 (0·7, 2·6)		0·5 (0·2, 1·2)	1·1 (0·7, 1·6)	1·2 (0·2, 6·3)
ETEC- unknown subgroup	1·6 (1·4, 1·9)	0·4 (0·1, 1·6)	1·4 (0·8, 2·4)	0·3 (0·1, 0·7)	3·1 (0·8, 12·7)	0·5 (0·2, 1·1)	1·3 (0·9, 1·9)	1·4 (0·1, 30·8)
Salmonella	1·4 (1·2, 1·8)	1·1 (0·5, 2·3)	1·1 (0·2, 6·4)			1·0 (0·6, 1·5)	1·3 (0·7, 2·2)	0·9 (0·6, 1·3)
Shigella	3·0 (2·4, 3·9)	7·6 (3·2, 18·2)	4·0 (2·3, 6·9)			7·4 (3·6, 15·2)	3·7 (2·8, 5·1)	2·8 (1·5, 5·5)
Cryptosporidium	2·2 (1·8, 2·6)	2·5 (1·2, 5·2)	2·1 (0·7, 6·6)			1·5 (0·8, 2·8)	1·0 (0·5, 1·7)	3·4 (1·2, 9·6)
E. histolytica	1·3 (1·0, 1·6)		1·8 (0·4, 7·3)			0·6 (0·2, 1·9)	1·7 (1·0, 2·9)	1·2 (0·6, 2·3)
Giardia lamblia	1·0 (0·8, 1·2)	0·4 (0·2, 0·6)	0·9 (0·1, 6·5)	0·2 (0·0, 0·9)		0·6 (0·4, 0·9)	0·9 (0·5, 1·7)	1·2 (0·6, 2·3)

[a] Unadjusted model results represent the crude summary estimate for a given pathogen.
[b] Adjusted models stratified by age and child mortality setting and adjusted for pathogen detection method (reference: conventional detection methods) and study design (reference: case-control study design).
[c] Pre-vaccine defined as a study that began prior to nationwide introduction of the rotavirus vaccine, post-vaccine defined as a study that began 1 or more years after nationwide introduction of the rotavirus vaccine. Rotavirus vaccine introduction status determined according to the WHO.
Blanks represent pathogens for which data were available in the analytic dataset but with too few effect estimates available to produce a summary OR.
CI, confidence interval; *E. histolytica*, *Entamoeba histolytica;* aEPEC, atypical enteropathogenic *E. coli;* tEPEC, typical enteropathogenic *E. coli;* ST ETEC, heat-stable enterotoxigenic *E. coli (*enterotoxigenic *E. coli* that harbor the ST gene regardless of LT gene status)*;* LT ETEC, heat-labile enterotoxigenic *E. coli (*enterotoxigenic *E. coli* that only harbor the LT gene and not the ST gene); OR, odds ratio*; V. cholerae*, *Vibrio cholerae.*

Table S11. Random effects meta-analysis results from unadjusted model and adjusted model stratified by age group and child mortality setting, redefining child age categories [a]

	Unadjusted model [b]	Stratified and adjusted models [c]						
		0-1 years of age		2-4 years of age		0-4 years of age		≥5 years of age
		Very low/low child mortality	High child mortality	Very low/low child mortality	High child mortality	Very low/low child mortality	High child mortality	All child mortality levels
**Enteropathogen**	**OR (95% CI)**	**OR (95% CI)**	**OR (95% CI)**	**OR (95% CI)**	**OR (95% CI)**	**OR (95% CI)**	**OR (95% CI)**	**OR (95% CI)**
Adenovirus 40/41	1·7 (1·4, 2·1)	8·6 (2·1, 35·8)	1·2 (0·3, 5·0)		1·2 (0·9, 1·7)	8·6 (2·1, 35·8)	1·2 (0·3, 4·8)	0·4 (0, 6·9)
Astrovirus	1·5 (1·4, 1·7)	1·4 (1·1, 1·9)	3·4 (1·4, 8·4)		1·6 (1·1, 2·3)	1·4 (1·1, 1·9)	3·4 (1·4, 8·3)	2·0 (0·4, 11·1)
Norovirus	1·5 (1·4, 1·7)	1·5 (1·1, 2·0)	3·2 (1·0, 10·3)		1·1 (0·8, 1·5)	1·5 (1·1, 2·0)	3·2 (1·0, 10·1)	3·1 (1·6, 5·8)
Rotavirus	5·4 (4·5, 6·6)	3·9 (0·8, 18·3)	18·1 (9·2, 35·8)		2·7 (1·6, 4·4)	3·9 (0·8, 18·3)	16·3 (8·2, 32·4)	2·9 (1·3, 6·5)
Pre-vaccine [d]	5·5 (4·5, 6·8)	3·9 (0·7, 22·0)	18·1 (9·2, 35·8)		2·7 (1·6, 4·4)	3·9 (0·7, 22·0)	16·3 (8·2, 32·4)	2·9 (1·3, 6·5)
Post-vaccine [d]	2·3 (1·5, 3·4)	1·5 (0·2, 9·4)				1·5 (0·2, 9·4)		
Sapovirus	1·5 (1·4, 1·7)	2·0 (1·7, 2·4)	1·4 (1·2, 1·6)		1·5 (1·1, 2·0)	2·0 (1·7, 2·4)	1·4 (1·2, 1·6)	
Aeromonas	2·6 (1·9, 3·6)		2·2 (1·3, 3·9)		3·4 (1·3, 8·9)		2·5 (1·6, 4·0)	1·5 (0·9, 2·6)
Campylobacter	1·6 (1·4, 1·7)	1·3 (0·8, 2·2)	1·9 (1·4, 2·8)		1·6 (0·8, 3·4)	1·3 (0·8, 2·2)	1·9 (1·4, 2·6)	5·1 (2·6, 10·0)
V. cholerae	7·3 (2·1, 25·3)		6·8 (1·4, 32·9)				6·8 (1·4, 32·9)	54·1 (7·4, 393·5)
aEPEC	0·8 (0·8, 0·9)	0·9 (0·7, 1·2)	0·7 (0·7, 0·8)		0·7 (0·6, 0·9)	0·9 (0·7, 1·2)	0·8 (0·7, 0·8)	1·3 (0·9, 1·7)
tEPEC	1·2 (1·0, 1·3)	1·7 (0·8, 4·0)	1·1 (1·0, 1·3)		1·0 (0·8, 1·2)	1·7 (0·8, 4·0)	1·1 (1·0, 1·2)	0·5 (0·1, 2·1)
EPEC- unknown subgroup	1·4 (1·2, 1·7)	1·0 (0·3, 3·5)	2·3 (1·5, 3·5)	0·8 (0·4, 1·6)	2·9 (0·7, 11·5)	0·9 (0·3, 2·5)	2·3 (1·5, 3·5)	2·0 (1·1, 3·5)
ST ETEC	1·7 (1·6, 1·9)	2·0 (0·5, 8·1)	1·2 (0·8, 1·7)	2·9 (1·4, 6·2)	2·8 (0·9, 8·5)	2·0 (0·5, 8·0)	1·6 (1·0, 2·4)	5·2 (2·3, 11·5)
LT ETEC	0·9 (0·9, 1·0)	1·4 (0·5, 4·0)	1·8 (0·9, 3·6)	1·8 (1·0, 3·3)	0·4 (0·0, 4·8)	1·3 (0·7, 2·5)	1·6 (0·8, 3·1)	1·2 (0·2, 6·3)
ETEC- unknown subgroup	1·6 (1·4, 1·9)		1·1 (0·5, 2·4)	2·1 (1·2, 3·4)		0·7 (0·1, 5·2)	1·3 (0·7, 2·7)	1·4 (0·1, 27·0)
Salmonella	1·5 (1·2, 1·8)	1·0 (0·3, 3·2)	0·2 (0·0, 2·1)			1·0 (0·3, 3·2)	0·2 (0·0, 2·1)	0·9 (0·6, 1·3)
Shigella	2·8 (2·4, 3·3)	3·1 (0·2, 45·5)	3·5 (1·0, 12·0)		2·3 (0·3, 18·6)	3·1 (0·2, 45·5)	2·9 (1·0, 8·2)	2·8 (1·5, 5·5)
Cryptosporidium	1·7 (1·5, 1·9)	2·4 (1·5, 3·8)	1·4 (1·2, 1·6)		1·1 (0·9, 1·3)	2·4 (1·5, 3·8)	1·3 (1·2, 1·5)	2·9 (1·1, 7·5)
E. histolytica	1·3 (1·1, 1·6)							1·2 (0·6, 2·3)
Giardia lamblia	1·0 (0·9, 1·2)	0·6 (0·2, 1·8)	0·9 (0·5, 1·7)	0·2 (0·0, 0·9)		0·6 (0·2, 1·8)	0·9 (0·5, 1·7)	1·1 (0·6, 1·9)

[a] Restricting age categorizations such that they do not make any assumptions about age maximums among children. Studies for which the age maximum was 2 years were included in the 2-4 year age category. Studies for which the age maximum was 5 years were included in the ≥5-year age category. In other analyses within this meta-analysis, studies indicating 2- or 5-year age maximums were included in the younger age group (0-1 and 2-4, respectively).
[b] Unadjusted model results represent the crude summary estimate for a given pathogen.
[c] Adjusted models stratified by age and child mortality setting and adjusted for pathogen detection method (reference: conventional detection methods) and study design (reference: case-control study design).
[d] Pre-vaccine defined as a study that began prior to nationwide introduction of the rotavirus vaccine, post-vaccine defined as a study that began 1 or more years after nationwide introduction of the rotavirus vaccine. Rotavirus vaccine introduction status determined according to the WHO.
Blanks represent pathogens for which data were available in the analytic dataset but with too few effect estimates available to produce a summary OR.

CI, confidence interval; *E. histolytica*, *Entamoeba histolytica;* aEPEC, atypical enteropathogenic *E. coli;* tEPEC, typical enteropathogenic *E. coli;* ST ETEC, heat-stable enterotoxigenic *E. coli (*enterotoxigenic *E. coli* that harbor the ST gene regardless of LT gene status)*;* LT ETEC, heat-labile enterotoxigenic *E. coli (*enterotoxigenic *E. coli* that only harbor the LT gene and not the ST gene); OR, odds ratio*; V. cholerae*, *Vibrio cholerae.*

Table S12. Random effects meta-analysis results from adjusted models for children 0-4 years of age stratified by region.

	Stratified and adjusted models [a]	
	**African Region [b]**	**Asian Region [c]**
**Enteropathogen**	**OR (95% CI)**	**OR (95% CI)**
Adenovirus 40/41	1·3 (0·8, 2·2)	1·7 (1·1, 2·5)
Astrovirus	1·7 (1, 2·8)	1·7 (1·5, 1·9)
Norovirus	2·9 (1·6, 5·5)	1·1 (0·9, 1·3)
Rotavirus	12·1 (5·9, 25·0)	13·4 (5·9, 30·5)
Pre-vaccine [d]	11·3 (5·9, 21·8)	13·4 (5·9, 30·5)
Post-vaccine [d]		
Sapovirus	1·2 (1·0, 1·4)	1·7 (1·4, 2·1)
Aeromonas	2·1 (1·0, 4·4)	2·8 (1·9, 4·1)
Campylobacter	1·5 (1·0, 2·3)	1·5 (1·1, 2·0)
V. cholerae		6·8 (1·4, 32·9)
aEPEC	0·7 (0·6, 0·8)	0·7 (0·6, 0·9)
tEPEC	1·2 (1·1, 1·4)	1·0 (0·9, 1·3)
EPEC- unknown subgroup	1·0 (0·3, 3·8)	2·9 (1·8, 4·7)
ST ETEC	1·4 (0·7, 2·6)	3·8 (2·7, 5·5)
LT ETEC	0·8 (0·7, 0·9)	1·5 (1·1, 2·0)
ETEC- unknown subgroup	1·0 (0·7, 1·3)	1·6 (0·6, 4·4)
Salmonella	1·1 (0·6, 2·0)	
Shigella	2·9 (1·7, 5·2)	3·6 (1·3, 9·8)
Cryptosporidium	1·4 (0·4, 5·0)	0·6 (0·2, 1·3)
E. histolytica	1·5 (0·7, 3·2)	1·6 (0·8, 3·0)
Giardia lamblia	1·2 (0·6, 2·3)	0·8 (0·2, 2·4)

[a] Models stratified by region and adjusted for pathogen detection method (reference: conventional detection methods) and study design (reference: case-control study design).
[b] African region defined as countries included in the WHO Regional Office of Africa.
[c] South/Southeast Asian region defined as countries included in the WHO Regional Office for South-East Asia.
[d] Pre-vaccine defined as a study that began prior to nationwide introduction of the rotavirus vaccine, post-vaccine defined as a study that began 1 or more years after nationwide introduction of the rotavirus vaccine. Rotavirus vaccine introduction status determined according to the World Health Organization.
Blanks represent pathogens for which data were available in the analytic dataset but with too few effect estimates available to produce a summary OR.
CI, confidence interval; *E. histolytica*, *Entamoeba histolytica;* aEPEC, atypical enteropathogenic *E. coli;* tEPEC, typical enteropathogenic *E. coli;* ST ETEC, heat-stable enterotoxigenic *E. coli (*enterotoxigenic *E. coli* that harbor the ST gene regardless of LT gene status)*;* LT ETEC, heat-labile enterotoxigenic *E. coli (*enterotoxigenic *E. coli* that only harbor the LT gene and not the ST gene); OR, odds ratio; PCR, polymerase chain reaction; *V. cholerae*, *Vibrio cholerae*

References

1 Aboderin AO, Smith SI, Oyelese AO, Onipede AO, Zailani SB, Coker AO. Role of Campylobacter jejuni/coli in diarrhoea in Ile-Ife, Nigeria. *East Afr Med J* 2002; **79**: 423–6.

2 Abu-Elyazeed R, Wierzba TF, Mourad AS, *et al.* Epidemiology of enterotoxigenic Escherichia coli diarrhea in a pediatric cohort in a periurban area of lower Egypt. *J Infect Dis* 1999; **179**: 382–9.

3 Acosta GJ, Vigo NI, Durand D, *et al.* Diarrheagenic Escherichia coli: Prevalence and Pathotype Distribution in Children from Peruvian Rural Communities. *Am J Trop Med Hyg* 2016; **95**: 574–9.

4 Afset JE, Bevanger L, Romundstad P, Bergh K. Association of atypical enteropathogenic Escherichia coli (EPEC) with prolonged diarrhoea. *J Med Microbiol* 2004; **53**: 1137–44.

5 Albert MJ, Faruque AS, Faruque SM, Sack RB, Mahalanabis D. Case-control study of enteropathogens associated with childhood diarrhea in Dhaka, Bangladesh. *J Clin Microbiol* 1999; **37**: 3458–64.

6 Aminu M, Page NA, Ahmad AA, Umoh JU, Dewar J, Steele AD. Diversity of rotavirus VP7 and VP4 genotypes in Northwestern Nigeria. *J Infect Dis* 2010; **202 Suppl**: S198-204.

7 Apelt N, Hartberger C, Campe H, Löscher T. The Prevalence of Norovirus in returning international travelers with diarrhea. *BMC Infect Dis* 2010; **10**: 131.

8 Arthur JL, Higgins GD, Davidson GP, Givney RC, Ratcliff RM. A novel bocavirus associated with acute gastroenteritis in Australian children. *PLoS Pathog* 2009; **5**: e1000391.

9 Ashie GK, Mutocheluh M, Owusu M, *et al.* Microbial pathogens associated with acute childhood diarrhoea in Kumasi, Ghana. *BMC Res Notes* 2017; **10**: 264.

10 Ballard S-B, Reaves EJ, Luna CG, *et al.* Epidemiology and Genetic Characterization of Noroviruses among Adults in an Endemic Setting, Peruvian Amazon Basin, 2004-2011. *PloS One* 2015; **10**: e0131646.

11 Becker SL, Chatigre JK, Gohou J-P, *et al.* Combined stool-based multiplex PCR and microscopy for enhanced pathogen detection in patients with persistent diarrhoea and asymptomatic controls from Côte d'Ivoire. *Clin Microbiol Infect Off Publ Eur Soc Clin Microbiol Infect Dis* 2015; **21**: 591.e1-10.

12 Becker-Dreps S, Bucardo F, Vilchez S, *et al.* Etiology of childhood diarrhea after rotavirus vaccine introduction: a prospective, population-based study in Nicaragua. *Pediatr Infect Dis J* 2014; **33**: 1156–63.

13 Bhandari N, Bahl R, Dua T, Kumar R, Srivastava R. Role of protozoa as risk factors for persistent diarrhea. *Indian J Pediatr* 1999; **66**: 21–6.

14 Bodhidatta L, McDaniel P, Sornsakrin S, Srijan A, Serichantalergs O, Mason CJ. Case-control study of diarrheal disease etiology in a remote rural area in Western Thailand. *Am J Trop Med Hyg* 2010; **83**: 1106–9.

15 Bodhidatta L, Anuras S, Sornsakrin S, *et al.* Epidemiology and etiology of Traveler's diarrhea in Bangkok, Thailand, a case-control study. *Trop Dis Travel Med Vaccines* 2019; **5**: 9.

16 Bona M, Medeiros PH, Santos AK, *et al.* Virulence-related genes are associated with clinical and nutritional outcomes of Shigella/Enteroinvasive Escherichia coli pathotype infection in children from Brazilian semiarid region: A community case-control study. *Int J Med Microbiol IJMM* 2019; **309**: 151–8.

17 Boru WG, Kikuvi G, Omollo J, *et al.* Aetiology and factors associated with bacterial diarrhoeal diseases amongst urban refugee children in Eastleigh, Kenya: A case control study. *Afr J Lab Med* 2013; **2**: 63.

18 Bravo L, Fernández A, Núñez FÁ, *et al.* [Aeromonas spp asociated to acute diarrheic disease in Cuba: case-control study]. *Rev Chil Infectologia Organo Of Soc Chil Infectologia* 2012; **29**: 44–8.

19 Breurec S, Vanel N, Bata P, *et al.* Etiology and Epidemiology of Diarrhea in Hospitalized Children from Low Income Country: A Matched Case-Control Study in Central African Republic. *PLoS Negl Trop Dis* 2016; **10**: e0004283.

20 Brink A-K, Mahé C, Watera C, *et al.* Diarrhea, CD4 counts and enteric infections in a community-based cohort of HIV-infected adults in Uganda. *J Infect* 2002; **45**: 99–106.

21 Bruijnesteijn van Coppenraet LES, Dullaert-de Boer M, Ruijs GJHM, *et al.* Case-control comparison of bacterial and protozoan microorganisms associated with gastroenteritis: application of molecular detection. *Clin Microbiol Infect Off Publ Eur Soc Clin Microbiol Infect Dis* 2015; **21**: 592.e9-19.

22 Brunser O, Espinoza J, Figueroa G, *et al.* Field trial of an infant formula containing anti-rotavirus and anti-Escherichia coli milk antibodies from hyperimmunized cows. *J Pediatr Gastroenterol Nutr* 1992; **15**: 63–72.

23 Bueris V, Sircili MP, Taddei CR, *et al.* Detection of diarrheagenic Escherichia coli from children with and without diarrhea in Salvador, Bahia, Brazil. *Mem Inst Oswaldo Cruz* 2007; **102**: 839–44.

24 Cárcamo C, Hooton T, Wener MH, *et al.* Etiologies and manifestations of persistent diarrhea in adults with HIV-1 infection: a case-control study in Lima, Peru. *J Infect Dis* 2005; **191**: 11–9.

25 Cardemil CV, Sherchand JB, Shrestha L, *et al.* Pathogen-Specific Burden of Outpatient Diarrhea in Infants in Nepal: A Multisite Prospective Case-Control Study. *J Pediatr Infect Dis Soc* 2017; **6**: e75–85.

26 Cennimo D, Abbas A, Huang DB, Chiang T. The prevalence and virulence characteristics of enteroaggregative Escherichia coli at an urgent-care clinic in the USA: a case-control study. *J Med Microbiol* 2009; **58**: 403–7.

27 Chang H, Zhang L, Ge Y, *et al.* A Hospital-based Case-control Study of Diarrhea in Children in Shanghai. *Pediatr Infect Dis J* 2017; **36**: 1057–63.

28 Chhin S, Harwell JI, Bell JD, *et al.* Etiology of chronic diarrhea in antiretroviral-naive patients with HIV infection admitted to Norodom Sihanouk Hospital, Phnom Penh, Cambodia. *Clin Infect Dis Off Publ Infect Dis Soc Am* 2006; **43**: 925–32.

29 Clemens J, Savarino S, Abu-Elyazeed R, *et al.* Development of pathogenicity-driven definitions of outcomes for a field trial of a killed oral vaccine against enterotoxigenic Escherichia coli in Egypt: application of an evidence-based method. *J Infect Dis* 2004; **189**: 2299–307.

30 Cohen D, Shoham O, Orr N, Muhsen K. An inverse and independent association between Helicobacter pylori infection and the incidence of shigellosis and other diarrheal diseases. *Clin Infect Dis Off Publ Infect Dis Soc Am* 2012; **54**: e35-42.

31 Contreras CA, Ochoa TJ, Lacher DW, *et al.* Allelic variability of critical virulence genes (eae, bfpA and perA) in typical and atypical enteropathogenic Escherichia coli in Peruvian children. *J Med Microbiol* 2010; **59**: 25–31.

32 Cranendonk RJ, Kodde CJ, Chipeta D, Zijlstra EE, Sluiters JF. Cryptosporidium parvum and Isospora belli infections among patients with and without diarrhoea. *East Afr Med J* 2003; **80**: 398–401.

33 da Silva Quetz J, Lima IFN, Havt A, *et al.* Campylobacter jejuni and Campylobacter coli in children from communities in Northeastern Brazil: molecular detection and relation to nutritional status. *Diagn Microbiol Infect Dis* 2010; **67**: 220–7.

34 de Wit MA, Koopmans MP, Kortbeek LM, van Leeuwen NJ, Vinjé J, van Duynhoven YT. Etiology of gastroenteritis in sentinel general practices in the netherlands. *Clin Infect Dis Off Publ Infect Dis Soc Am* 2001; **33**: 280–8.

35 Denno DM, Shaikh N, Stapp JR, *et al.* Diarrhea etiology in a pediatric emergency department: a case control study. *Clin Infect Dis Off Publ Infect Dis Soc Am* 2012; **55**: 897–904.

36 Do TT, Bui TTH, Mølbak K, Phung DC, Dalsgaard A. Epidemiology and aetiology of diarrhoeal diseases in adults engaged in wastewater-fed agriculture and aquaculture in Hanoi, Vietnam. *Trop Med Int Health TM IH* 2007; **12 Suppl 2**: 23–33.

37 Dutta SR, Khalfan SA, Baig BH, Philipose L, Fulayfil R. Epidemiology of rotavirus diarrhoea in children under five years in Bahrain. *Int J Epidemiol* 1990; **19**: 722–7.

38 Dwivedi KK, Prasad G, Saini S, Mahajan S, Lal S, Baveja UK. Enteric opportunistic parasites among HIV infected individuals: associated risk factors and immune status. *Jpn J Infect Dis* 2007; **60**: 76–81.

39 Eibach D, Krumkamp R, Hahn A, *et al.* Application of a multiplex PCR assay for the detection of gastrointestinal pathogens in a rural African setting. *BMC Infect Dis* 2016; **16**: 150.

40 El-Hakim MA, El-Sahn A. Association of parasites and diarrhoea among children less than five years of age in a rural area in Egypt. *J Egypt Public Health Assoc* 1996; **71**: 439–63.

41 El-Shabrawi M, Salem M, Abou-Zekri M, *et al.* The burden of different pathogens in acute diarrhoeal episodes among a cohort of Egyptian children less than five years old. *Przeglad Gastroenterol* 2015; **10**: 173–80.

42 Espinoza F, Paniagua M, Hallander H, Svensson L, Strannegård O. Rotavirus infections in young Nicaraguan children. *Pediatr Infect Dis J* 1997; **16**: 564–71.

43 Fang GD, Lima AA, Martins CV, Nataro JP, Guerrant RL. Etiology and epidemiology of persistent diarrhea in northeastern Brazil: a hospital-based, prospective, case-control study. *J Pediatr Gastroenterol Nutr* 1995; **21**: 137–44.

44 Farfán-García AE, Zhang C, Imdad A, *et al.* Case-Control Pilot Study on Acute Diarrheal Disease in a Geographically Defined Pediatric Population in a Middle Income Country. *Int J Pediatr* 2017; **2017**: 6357597.

45 Fathy MM, Abdelrazek NM, Hassan FA, El-Badry AA. Molecular copro-prevalence of Cryptosporidium in Egyptian children and evaluation of three diagnostic methods. *Indian Pediatr* 2014; **51**: 727–9.

46 Firdu T, Abunna F, Girma M. Intestinal Protozoal Parasites in Diarrheal Children and Associated Risk Factors at Yirgalem Hospital, Ethiopia: A Case-Control Study. *Int Sch Res Not* 2014; **2014**: 357126.

47 François R, Yori PP, Rouhani S, *et al.* The other Campylobacters: Not innocent bystanders in endemic diarrhea and dysentery in children in low-income settings. *PLoS Negl Trop Dis* 2018; **12**: e0006200.

48 Fraser D, Dagan R, Naggan L, *et al.* Natural history of Giardia lamblia and Cryptosporidium infections in a cohort of Israeli Bedouin infants: a study of a population in transition. *Am J Trop Med Hyg* 1997; **57**: 544–9.

49 Fraser D, Dagan R, Porat N, *et al.* Persistent diarrhea in a cohort of Israeli Bedouin infants: role of enteric pathogens and family and environmental factors. *J Infect Dis* 1998; **178**: 1081–8.

50 Gascón J, Vargas M, Quintó L, Corachán M, Jimenez de Anta MT, Vila J. Enteroaggregative Escherichia coli strains as a cause of traveler's diarrhea: a case-control study. *J Infect Dis* 1998; **177**: 1409–12.

51 Gascón J, Vargas M, Schellenberg D, *et al.* Diarrhea in children under 5 years of age from Ifakara, Tanzania: a case-control study. *J Clin Microbiol* 2000; **38**: 4459–62.

52 Gassama A, Sow PS, Fall F, *et al.* Ordinary and opportunistic enteropathogens associated with diarrhea in Senegalese adults in relation to human immunodeficiency virus serostatus. *Int J Infect Dis IJID Off Publ Int Soc Infect Dis* 2001; **5**: 192–8.

53 Georges-Courbot MC, Cassel-Beraud AM, Gouandjika I, Monges J, Georges AJ. A cohort study of enteric campylobacter infection in children from birth to two years in Bangui (Central African Republic). *Trans R Soc Trop Med Hyg* 1990; **84**: 122–5.

54 Gomez-Duarte OG, Romero-Herazo YC, Paez-Canro CZ, Eslava-Schmalbach JH, Arzuza O. Enterotoxigenic Escherichia coli associated with childhood diarrhoea in Colombia, South America. *J Infect Dev Ctries* 2013; **7**: 372–81.

55 Haque R, Mondal D, Karim A, *et al.* Prospective case-control study of the association between common enteric protozoal parasites and diarrhea in Bangladesh. *Clin Infect Dis Off Publ Infect Dis Soc Am* 2009; **48**: 1191–7.

56 Hien BTT, Trang DT, Scheutz F, Cam PD, Mølbak K, Dalsgaard A. Diarrhoeagenic Escherichia coli and other causes of childhood diarrhoea: a case-control study in children living in a wastewater-use area in Hanoi, Vietnam. *J Med Microbiol* 2007; **56**: 1086–96.

57 Hien BTT, Scheutz F, Cam PD, *et al.* Diarrheagenic Escherichia coli and Shigella strains isolated from children in a hospital case-control study in Hanoi, Vietnam. *J Clin Microbiol* 2008; **46**: 996–1004.

58 Hoge CW, Shlim DR, Echeverria P, Rajah R, Herrmann JE, Cross JH. Epidemiology of diarrhea among expatriate residents living in a highly endemic environment. *JAMA* 1996; **275**: 533–8.

59 Holtz LR, Bauer IK, Rajendran P, Kang G, Wang D. Astrovirus MLB1 is not associated with diarrhea in a cohort of Indian children. *PloS One* 2011; **6**: e28647.

60 Isenbarger DW, Hien BT, Ha HT, *et al.* Prospective study of the incidence of diarrhoea and prevalence of bacterial pathogens in a cohort of Vietnamese children along the Red River. *Epidemiol Infect* 2001; **127**: 229–36.

61 Iturriza-Gómara M, Jere KC, Hungerford D, *et al.* Etiology of Diarrhea Among Hospitalized Children in Blantyre, Malawi, Following Rotavirus Vaccine Introduction: A Case-Control Study. *J Infect Dis* 2019; **220**: 213–8.

62 Jain D, Sinha S, Prasad KN, Pandey CM. Campylobacter species and drug resistance in a north Indian rural community. *Trans R Soc Trop Med Hyg* 2005; **99**: 207–14.

63 Krumkamp R, Sarpong N, Schwarz NG, *et al.* Gastrointestinal infections and diarrheal disease in Ghanaian infants and children: an outpatient case-control study. *PLoS Negl Trop Dis* 2015; **9**: e0003568.

64 Lee G, Pan W, Peñataro Yori P, *et al.* Symptomatic and asymptomatic Campylobacter infections associated with reduced growth in Peruvian children. *PLoS Negl Trop Dis* 2013; **7**: e2036.

65 Levine MM, Ferreccio C, Prado V, *et al.* Epidemiologic studies of Escherichia coli diarrheal infections in a low socioeconomic level peri-urban community in Santiago, Chile. *Am J Epidemiol* 1993; **138**: 849–69.

66 Li LL, Liu N, Humphries EM, *et al.* Aetiology of diarrhoeal disease and evaluation of viral-bacterial coinfection in children under 5 years old in China: a matched case-control study. *Clin Microbiol Infect Off Publ Eur Soc Clin Microbiol Infect Dis* 2016; **22**: 381.e9-381.e16.

67 Lima AAM, Oliveira DB, Quetz JS, *et al.* Etiology and severity of diarrheal diseases in infants at the semiarid region of Brazil: A case-control study. *PLoS Negl Trop Dis* 2019; **13**: e0007154.

68 Liu X, Jahuira H, Gilman RH, *et al.* Etiological Role and Repeated Infections of Sapovirus among Children Aged Less than 2 Years in a Cohort Study in a Peri-urban Community of Peru. *J Clin Microbiol* 2016; **54**: 1598–604.

69 Lule JR, Mermin J, Awor A, *et al.* Aetiology of diarrhoea among persons with HIV and their family members in rural Uganda: a community-based study. *East Afr Med J* 2009; **86**: 422–9.

70 Maldonado Y, Cantwell M, Old M, *et al.* Population-based prevalence of symptomatic and asymptomatic astrovirus infection in rural Mayan infants. *J Infect Dis* 1998; **178**: 334–9.

71 Mansour AM, Abd Elkhalek R, Shaheen HI, *et al.* Burden of Aeromonas hydrophila-associated diarrhea among children younger than 2 years in rural Egyptian community. *J Infect Dev Ctries* 2012; **6**: 842–6.

72 Mansour AM, Mohammady HE, Shabrawi ME, *et al.* Modifiable diarrhoea risk factors in Egyptian children aged <5 years. *Epidemiol Infect* 2013; **141**: 2547–59.

73 Mansour A, Shaheen HI, Amine M, *et al.* Diarrhea burden due to natural infection with enterotoxigenic Escherichia coli in a birth cohort in a rural Egyptian community. *J Clin Microbiol* 2014; **52**: 2595–603.

74 Mansour A, Shaheen HI, Amine M, *et al.* Pathogenicity and phenotypic characterization of enterotoxigenic Escherichia coli isolates from a birth cohort of children in rural Egypt. *J Clin Microbiol* 2014; **52**: 587–91.

75 Mason CJ, Sornsakrin S, Seidman JC, *et al.* Antibiotic resistance in Campylobacter and other diarrheal pathogens isolated from US military personnel deployed to Thailand in 2002-2004: a case-control study. *Trop Dis Travel Med Vaccines* 2017; **3**: 13.

76 Mégraud F, Boudraa G, Bessaoud K, *et al.* Incidence of Campylobacter infection in infants in western Algeria and the possible protective role of breast feeding. *Epidemiol Infect* 1990; **105**: 73–8.

77 Mercado EH, Ochoa TJ, Ecker L, *et al.* Fecal leukocytes in children infected with diarrheagenic Escherichia coli. *J Clin Microbiol* 2011; **49**: 1376–81.

78 Ming ZF, Xi ZD, Dong CS, *et al.* Diarrhoeal disease in children less than one year of age at a children's hospital in Guangzhou, People's Republic of China. *Trans R Soc Trop Med Hyg* 1991; **85**: 667–9.

79 Mitra S, Mukherjee A, Khanra D, Bhowmik A, Roy K, Talukdar A. Enteric Parasitic Infection Among Antiretroviral Therapy Naïve HIV-Seropositive People: Infection Begets Infection-Experience from Eastern India. *J Glob Infect Dis* 2016; **8**: 82–6.

80 Mota-Hernández F, Calva JJ, Gutiérrez-Camacho C, *et al.* Rotavirus diarrhea severity is related to the VP4 type in Mexican children. *J Clin Microbiol* 2003; **41**: 3158–62.

81 Moyo SJ, Hanevik K, Blomberg B, *et al.* Prevalence and molecular characterisation of human adenovirus in diarrhoeic children in Tanzania; a case control study. *BMC Infect Dis* 2014; **14**: 666.

82 Muhsen K, Cohen D, Levine MM. Can Giardia lamblia infection lower the risk of acute diarrhea among preschool children? *J Trop Pediatr* 2014; **60**: 99–103.

83 Mullick S, Mukherjee A, Ghosh S, *et al.* Community based case-control study of rotavirus gastroenteritis among young children during 2008-2010 reveals vast genetic diversity and increased prevalence of G9 strains in Kolkata. *PloS One* 2014; **9**: e112970.

84 My PVT, Thompson C, Phuc HL, *et al.* Endemic norovirus infections in children, Ho Chi Minh City, Vietnam, 2009-2010. *Emerg Infect Dis* 2013; **19**: 977–80.

85 Nelson MI, Mahfuz M, Chhabra P, *et al.* Genetic Diversity of Noroviruses Circulating in a Pediatric Cohort in Bangladesh. *J Infect Dis* 2018; **218**: 1937–42.

86 Newman RD, Sears CL, Moore SR, *et al.* Longitudinal study of Cryptosporidium infection in children in northeastern Brazil. *J Infect Dis* 1999; **180**: 167–75.

87 Newman RD, Moore SR, Lima AA, Nataro JP, Guerrant RL, Sears CL. A longitudinal study of Giardia lamblia infection in north-east Brazilian children. *Trop Med Int Health TM IH* 2001; **6**: 624–34.

88 Nimri LF, Hijazi SS. Cryptosporidium. A cause of gastroenteritis in preschool children in Jordan. *J Clin Gastroenterol* 1994; **19**: 288–91.

89 Nimri LF, Hijazi S. Rotavirus-associated diarrhoea in children in a refugee camp in Jordan. *J Diarrhoeal Dis Res* 1996; **14**: 1–4.

90 O'Ryan ML, Lucero Y, Prado V, *et al.* Symptomatic and asymptomatic rotavirus and norovirus infections during infancy in a Chilean birth cohort. *Pediatr Infect Dis J* 2009; **28**: 879–84.

91 Ochoa TJ, Ruiz J, Molina M, *et al.* High frequency of antimicrobial drug resistance of diarrheagenic Escherichia coli in infants in Peru. *Am J Trop Med Hyg* 2009; **81**: 296–301.

92 Oketcho R, Nyaruhucha CNM, Taybalip S, Karimuribo ED. Influence of enteric bacteria, parasite infections and nutritional status on diarrhoea occurrence among 6-60 months old children admitted at a Regional Hospital in Morogoro, Tanzania. *Tanzan J Health Res* 2012; **14**: 104–14.

93 Olesen B, Neimann J, Böttiger B, *et al.* Etiology of diarrhea in young children in Denmark: a case-control study. *J Clin Microbiol* 2005; **43**: 3636–41.

94 Pandey P, Bodhidatta L, Lewis M, *et al.* Travelers' diarrhea in Nepal: an update on the pathogens and antibiotic resistance. *J Travel Med* 2011; **18**: 102–8.

95 Paniagua M, Espinoza F, Ringman M, Reizenstein E, Svennerholm AM, Hallander H. Analysis of incidence of infection with enterotoxigenic Escherichia coli in a prospective cohort study of infant diarrhea in Nicaragua. *J Clin Microbiol* 1997; **35**: 1404–10.

96 Pavie J, Menotti J, Porcher R, *et al.* Prevalence of opportunistic intestinal parasitic infections among HIV-infected patients with low CD4 cells counts in France in the combination antiretroviral therapy era. *Int J Infect Dis IJID Off Publ Int Soc Infect Dis* 2012; **16**: e677-679.

97 Pazzaglia G, Bourgeois AL, el Diwany K, Nour N, Badran N, Hablas R. Campylobacter diarrhoea and an association of recent disease with asymptomatic shedding in Egyptian children. *Epidemiol Infect* 1991; **106**: 77–82.

98 Liu J, Platts-Mills JA, Juma J, *et al.* Use of quantitative molecular diagnostic methods to identify causes of diarrhoea in children: a reanalysis of the GEMS case-control study. *The Lancet* 2016; **388**: 1291–301.

99 Platts-Mills JA, Liu J, Rogawski ET, *et al.* Use of quantitative molecular diagnostic methods to assess the aetiology, burden, and clinical characteristics of diarrhoea in children in low-resource settings: a reanalysis of the MAL-ED cohort study. *Lancet Glob Health* 2018; **6**: e1309–18.

100 Qadri F, Saha A, Ahmed T, Al Tarique A, Begum YA, Svennerholm A-M. Disease burden due to enterotoxigenic Escherichia coli in the first 2 years of life in an urban community in Bangladesh. *Infect Immun* 2007; **75**: 3961–8.

101 Randremanana R, Randrianirina F, Gousseff M, *et al.* Case-control study of the etiology of infant diarrheal disease in 14 districts in Madagascar. *PloS One* 2012; **7**: e44533.

102 Randremanana RV, Randrianirina F, Sabatier P, *et al.* Campylobacter infection in a cohort of rural children in Moramanga, Madagascar. *BMC Infect Dis* 2014; **14**: 372.

103 Randremanana RV, Razafindratsimandresy R, Andriatahina T, *et al.* Etiologies, Risk Factors and Impact of Severe Diarrhea in the Under-Fives in Moramanga and Antananarivo, Madagascar. *PloS One* 2016; **11**: e0158862.

104 Rao MR, Naficy AB, Savarino SJ, *et al.* Pathogenicity and convalescent excretion of Campylobacter in rural Egyptian children. *Am J Epidemiol* 2001; **154**: 166–73.

105 Reyes D, Vilchez S, Paniagua M, *et al.* Diversity of intestinal Escherichia coli populations in Nicaraguan children with and without diarrhoea. *J Med Microbiol* 2009; **58**: 1593–600.

106 Saito M, Goel-Apaza S, Espetia S, *et al.* Multiple norovirus infections in a birth cohort in a Peruvian Periurban community. *Clin Infect Dis Off Publ Infect Dis Soc Am* 2014; **58**: 483–91.

107 Sallon S, el Showwa R, el Masri M, Khalil M, Blundell N, Hart CA. Cryptosporidiosis in children in Gaza. *Ann Trop Paediatr* 1991; **11**: 277–81.

108 Scaletsky IC, Pedroso MZ, Morais MB, *et al.* [Association of patterns of Escherichia coli adherence to HEp-2 cells with acute and persistent diarrhea]. *Arq Gastroenterol* 1999; **36**: 54–60.

109 Scaletsky ICA, Fabbricotti SH, Carvalho RLB, *et al.* Diffusely adherent Escherichia coli as a cause of acute diarrhea in young children in Northeast Brazil: a case-control study. *J Clin Microbiol* 2002; **40**: 645–8.

110 Schorling JB, Wanke CA, Schorling SK, McAuliffe JF, de Souza MA, Guerrant RL. A prospective study of persistent diarrhea among children in an urban Brazilian slum. Patterns of occurrence and etiologic agents. *Am J Epidemiol* 1990; **132**: 144–56.

111 Schultsz C, van den Ende J, Cobelens F, *et al.* Diarrheagenic Escherichia coli and acute and persistent diarrhea in returned travelers. *J Clin Microbiol* 2000; **38**: 3550–4.

112 Serichantalergs O, Ruekit S, Pandey P, *et al.* Incidence of Campylobacter concisus and C. ureolyticus in traveler's diarrhea cases and asymptomatic controls in Nepal and Thailand. *Gut Pathog* 2017; **9**: 47.

113 Shen X-X, Qiu F-Z, Li G-X, *et al.* A case control study on the prevalence of enterovirus in children samples and its association with diarrhea. *Arch Virol* 2019; **164**: 63–8.

114 Soltan Dallal MM, Moezardalan K. Aeromonas spp associated with children's diarrhoea in Tehran: a case-control study. *Ann Trop Paediatr* 2004; **24**: 45–51.

115 Sow D, Dogue F, Edouard S, *et al.* Acquisition of enteric pathogens by pilgrims during the 2016 Hajj pilgrimage: A prospective cohort study. *Travel Med Infect Dis* 2018; **25**: 26–30.

116 Steiner KL, Ahmed S, Gilchrist CA, *et al.* Species of Cryptosporidia Causing Subclinical Infection Associated With Growth Faltering in Rural and Urban Bangladesh: A Birth Cohort Study. *Clin Infect Dis Off Publ Infect Dis Soc Am* 2018; **67**: 1347–55.

117 Steinsland H, Valentiner-Branth P, Perch M, *et al.* Enterotoxigenic Escherichia coli infections and diarrhea in a cohort of young children in Guinea-Bissau. *J Infect Dis* 2002; **186**: 1740–7.

118 Swierczewski BE, Odundo EA, Koech MC, *et al.* Surveillance for enteric pathogens in a case-control study of acute diarrhea in Western Kenya. *Trans R Soc Trop Med Hyg* 2013; **107**: 83–90.

119 Taniuchi M, Sobuz SU, Begum S, *et al.* Etiology of diarrhea in Bangladeshi infants in the first year of life analyzed using molecular methods. *J Infect Dis* 2013; **208**: 1794–802.

120 Tellevik MG, Moyo SJ, Blomberg B, *et al.* Prevalence of Cryptosporidium parvum/hominis, Entamoeba histolytica and Giardia lamblia among Young Children with and without Diarrhea in Dar es Salaam, Tanzania. *PLoS Negl Trop Dis* 2015; **9**: e0004125.

121 Tumwine JK, Kekitiinwa A, Nabukeera N, *et al.* Cryptosporidium parvum in children with diarrhea in Mulago Hospital, Kampala, Uganda. *Am J Trop Med Hyg* 2003; **68**: 710–5.

122 Valentiner-Branth P, Steinsland H, Fischer TK, *et al.* Cohort study of Guinean children: incidence, pathogenicity, conferred protection, and attributable risk for enteropathogens during the first 2 years of life. *J Clin Microbiol* 2003; **41**: 4238–45.

123 Vasco G, Trueba G, Atherton R, *et al.* Identifying etiological agents causing diarrhea in low income Ecuadorian communities. *Am J Trop Med Hyg* 2014; **91**: 563–9.

124 Vernacchio L, Vezina RM, Mitchell AA, Lesko SM, Plaut AG, Acheson DWK. Characteristics of persistent diarrhea in a community-based cohort of young US children. *J Pediatr Gastroenterol Nutr* 2006; **43**: 52–8.

125 Vernacchio L, Vezina RM, Mitchell AA, Lesko SM, Plaut AG, Acheson DWK. Diarrhea in American infants and young children in the community setting: incidence, clinical presentation and microbiology. *Pediatr Infect Dis J* 2006; **25**: 2–7.

126 Vethanayagam RR, Ananda Babu M, Nagalaxmi KS, *et al.* Possible role of neonatal infection with the asymptomatic reassortant rotavirus (RV) strain I321 in the decrease in hospital admissions for RV diarrhea, Bangalore, India, 1988-1999. *J Infect Dis* 2004; **189**: 2282–9.

127 Viboud GI, Jouve MJ, Binsztein N, *et al.* Prospective cohort study of enterotoxigenic Escherichia coli infections in Argentinean children. *J Clin Microbiol* 1999; **37**: 2829–33.

128 Vieira N, Bates SJ, Solberg OD, *et al.* High prevalence of enteroinvasive Escherichia coli isolated in a remote region of northern coastal Ecuador. *Am J Trop Med Hyg* 2007; **76**: 528–33.

129 Yori PP, Schwab K, Gilman RH, *et al.* Norovirus highly prevalent cause of endemic acute diarrhea in children in the peruvian Amazon. *Pediatr Infect Dis J* 2009; **28**: 844–7.

130 Zhang S-X, Yang C-L, Gu W-P, *et al.* Case-control study of diarrheal disease etiology in individuals over 5 years in southwest China. *Gut Pathog* 2016; **8**: 58.
